# RNAi-mediated knockdown of *daf-12* in the model parasitic nematode *Strongyloides ratti*

**DOI:** 10.1371/journal.ppat.1007705

**Published:** 2019-03-29

**Authors:** Alex Dulovic, Adrian Streit

**Affiliations:** Department of Integrative Evolutionary Biology, Max Planck Institute for Developmental Biology, Tübingen, Baden-Württemberg, Germany; University of Pennsylvania, UNITED STATES

## Abstract

The gene *daf-12* has long shown to be involved in the dauer pathway in *Caenorhabditis elegans (C*. *elegans)*. Due to the similarities of the dauer larvae of *C*. *elegans* and infective larvae of certain parasitic nematodes such as *Strongyloides* spp., this gene has also been suspected to be involved in the development of infective larvae. Previous research has shown that the application of dafachronic acid, the steroid hormone ligand of DAF-12 in *C*. *elegans*, affects the development of infective larvae and metabolism in *Strongyloides*. However, a lack of tools for either forward or reverse genetics within *Strongyloides* has limited studies of gene function within these important parasites. After determining whether *Strongyloides* had the requisite proteins for RNAi, we developed and report here the first successful RNAi by soaking protocol for *Strongyloides ratti (S*. *ratti)* and use this protocol to study the functions of *daf-12* within *S*. *ratti*. Suppression of *daf-12* in *S*. *ratti* severely impairs the formation of infective larvae of the direct cycle and redirects development towards the non-infective (non-dauer) free-living life cycle. Further, *daf-12(RNAi) S*. *ratti* produce slightly but significantly fewer offspring and these offspring are developmentally delayed or incapable of completing their development to infective larvae (L3i). Whilst the successful *daf-12(RNAi)* L3i are still able to infect a new host, the resulting infection is less productive and shorter lived. Further, *daf-12* knockdown affects metabolism in *S*. *ratti* resulting in a shift from aerobic towards anaerobic fat metabolism. Finally, *daf-12(RNAi) S*. *ratti* have reduced tolerance of temperature stress.

## Author summary

*Strongyloides ratti* is a model parasitic nematode of interest for its use in understanding basic biology and the development of novel helminth therapies. However a lack of genetic tools has stymied progress, although CRISPR/Cas9 has recently been reported. After determining whether RNAi might work in *S*. *ratti* by profiling the RNAi pathway proteins, we developed a successful RNAi protocol, which was used to study the gene *daf-12*. In *Caenorhabditis elegans*, *daf-12* is involved in various developmental and metabolic processes, including the formation of long-living dauer larvae which are considered to be similar to the infective larvae of *Strongyloides*. Based on the external application of dafachronic acid (the ligand of DAF-12 in *C*. *elegans*) and gene expression studies, it was proposed that *daf-12* has conserved functions in *Strongyloides*. Using our RNAi method, we provide the first proof of successful gene knockdown within *S*. *ratti* and demonstrate that *daf-12* in *S*. *ratti* is involved in the same processes as *C*. *elegans*. This supports that *daf-12* functions are conserved in distantly related nematodes and that *daf-12* is an important target for the development of novel antihelminthics.

## Introduction

In order to survive, nematodes have to be able to adapt to their surrounding conditions. In the well-studied model organism *Caenorhabditis elegans*, worms are able to switch between rapid development through a third stage larvae and the development of long lived dauer third stage larvae, which enable them to survive unsatisfactory environmental conditions [1]. Similarly, certain parasitic nematodes also may alter their life cycle development based on their surrounding conditions. The progeny of parasitic *Strongyloides* spp. are able to switch between a dauer-like pathway, which results in the formation of infective third larvae (L3i) and the infection of a new host (direct cycle), and the formation of a single free-living generation through a different, rapidly developing third stage larvae (indirect cycle) [2]. For most characterized *Strongyloides* species (such as *S*. *ratti*), all progeny of the free-living adults develop into L3i, however for the cat-specific species (*Strongyloides planiceps*) [3], successive free-living generations are possible. For further details on the life cycle of *Strongyloides* we refer the reader to [4]. The L3i stage is believed to be homologous to the *C*. *elegans* dauer [4, 5]. Compared with the *C*. *elegans* dauer larvae, the mechanisms that control the formation of the *Strongyloides* spp. L3i is currently rather poorly understood.

Parasitic nematodes currently constitute a great threat to humankind, from causing wide scale economic loss to having direct implications on human health. One of the more dangerous of these nematodes is the parasitic roundworm *Strongyloides stercoralis*, which infects more than 350 million people [6]. Due to the occurrence of consecutive autoinfective cycles, chronic *S*. *stercoralis* infections can sustain themselves for years. While individuals infected with chronic strongyloidiasis are often asymptomatic or experience mild gastro-intestinal symptoms because they control the infection at a very low level, in immuno-compromised patients the auto infective cycles may self-enhance, a condition known as hyperinfection syndrome followed by disseminated strongyloidiasis, which is normally fatal [7]. While chronic or early stages of hyperinfecting *Strongyloides* can currently be treated with ivermectin or, to some extent, other antihelminthics [8], disseminated strongyloidiasis is often fatal in spite of successful killing of the worms because the organ damage was already too great and/or the masses of dying migrating larvae cause additional damage [8–10]. In addition, resistance towards the current antihelminthics used has been identified in animals and humans, meaning new treatment options to treat strongyloidiasis are necessary [11]. Of particular interest in developing new treatments are the molecular mechanisms associated with the formation of infective larvae, because prevention of the formation of autoinfective larvae could break the continuous autoinfective cycle responsible for the severe pathology of *S*. *stercoralis*.

In *C*. *elegans*, the nuclear hormone receptor DAF-12 is known to be involved as a key regulator in the dauer pathway [12] and to act as a convergence of the pathways regulating larval diapause, developmental age and adult longevity [13, 14]. The ligand for DAF-12 in *C*. *elegans* is dafachronic acid (DA) [15]. In its name giving function (daf: dauer formation defective), the receptor ligand interaction appears phenotypically inhibitory because the effects of mutating the receptor or exogenous application of DA are identical, namely inhibition of dauer formation [15]. However, DAF-12 has functions in both developmental pathways and at various time points during the ontogeny of a worm [1,13]. In addition to ligand binding, DAF-12 has been shown to interact with partners such as *daf-16* [16–18] and *din-1* [19, 20] to control developmental events and with *let-7* micro RNAs to mediate the immune response [21]. DAF-12 has also been implicated in several metabolic processes [14] such as the production of cytosolic NADPH [17] or fat metabolism [16]. Multiple genes involved in post-embryonic development have been shown to be regulated by DAF-12. In some cases, the same gene can be either repressed or activated, depending on developmental stage [15]. Further, different mutant alleles of *daf-12* within *C*. *elegans* have been found to effect both fertility and response to heat stress [22].

Pharmacological experiments using exogenous DA in *Strongyloides papillosus (S*. *papillosus)* and *S*. *stercoralis* suggested that a conserved endocrine module might regulate dauer/L3i formation and metabolism in *Strongyloides* spp. [5, 16, 23–26]. In particular, DA prevented the progeny of the parasitic generation from developing into L3i directly and redirected them to form free-living stages [5, 23, 26, 27]. In *S*. *papillosus*, DA application caused the progeny of the free-living generation to undergo an extra free-living generation [5]. DA was also shown to induce the activation of infective L3i in *S*. *stercoralis* [26] and to upregulate genes involved in aerobic and downregulate genes involved in anaerobic fatty acid metabolism [16]. In all these studies the pharmacological action of DA was suggested to occur through *Strongyloides*-DAF-12, which has been identified in *S*. *stercoralis* [27]. However, involvement of DAF-12 has never been directly demonstrated and, given that other nuclear hormone receptors exist and the similarity of the ligand binding domains in the *C*. *elegans* and the *Strongyloides* proteins is limited, action through DAF-12 could not be safely assumed.

*Strongyloides* spp. is not only of interest as a pathogen but, in part thanks to its free-living generation, it is also an attractive model system to study a number of biological questions, as has been outlined in a recent special issue of the journal "Parasitology" [28, 29]. A number of techniques for the molecular genetic analysis have been developed over the years, such as chemical mutagenesis [30, 31], genetic mapping [32] and transgenesis [33]. The full genome sequences of four species of *Strongyloides* have also been published along with transcriptomic information [34]. However for a long time, attempts to develop methods for reverse genetic knock out or knock down of gene function in *Strongyloides* spp. failed. Very recently, successful targeted mutagenesis using the CRISPR/Cas9 system has been reported for *S*. *stercoralis* but the same approach worked poorly by comparison in *S*. *ratti* [35], the species considered the prime non-human-pathogenic model species of *Strongyloides* [36]. RNA mediated interference (RNAi), an already a widely used technique in other nematodes [37, 38] would be a highly desirable technique because it would not require the establishment of mutant lines in hosts. While RNAi has been reported and successfully used for years within many plant parasitic nematodes (such as *Meloidogyne incognita* [39], *Heterodera glycine* [40] or *Globodera pallida* [40]), entomopathogenic parasitic nematodes (such as *Heterorhabditis bacteriophora* [41] or *Steinernema carpocapsae* [42]) and more recently within animal parasitic nematodes (such as *Haemonchus contortus* [43] *Ascaris suum* [44] or *Brugia malayi* [45]), it has yet to be successful within any *Strongyloides* species. The majority of the above cited papers involved the application of dsRNA which had been previously tested in *Strongyloides* spp. leading some authors to conclude that *Strongyloides* was refractive to RNAi [46]. However recent developments have involved the use of siRNAs for generating RNAi in animal parasitic nematodes [45, 47], as this bypasses the processing steps for dsRNA which several animal parasitic nematodes lack [48].

In this study, we report the development of a reliable method for RNAi in *S*. *ratti*, based on the protocol published earlier for *B*. *malayi* [45]. We decided to use the nuclear hormone receptor DAF-12 as a test case because the relatively numerous pharmacological studies with DA mentioned above provided us with a number of testable phenotypic expectations and made this gene a highly interesting target for knock down analysis. Our results are in full agreement with the hypothesis that the pharmacological effects seen with DA involved DAF-12, and that DAF-12 is a central part of a conserved endocrine module that controls multiple processes as different as dauer/L3i formation, fat metabolism and stress tolerance.

## Results

### Presence and absence of homologs of known *C*. *elegans* RNAi pathway genes in *Strongyloides* spp

To determine whether RNAi was likely to work within *Strongyloides*, we asked if the key proteins of the RNAi machinery in *C*. *elegans* (as described in [48]), are present in the *Strongyloides* spp. genomes. Since in a recently published comparison of the Argonaute family genes it had been found that members of the Argonaute families believed to be required for RNAi are present in Strongyloididae [49], this gene family was not analyzed again. For each of the selected *C*. *elegans* genes, we determined if homologues existed in *Strongyloides* spp. following the approach described by [48], which considers sequence similarity and domain architecture and if they were likely one to one orthologs or existed in a one (in *C*. *elegans* to many (two or more in *Strongyloides*)), or a many to one or a many to many relationship. A gene was only considered to be an ortholog if it contained all of the functional domains present in the *C*. *elegans* protein. As can be seen in Table 1, *Strongyloides* spp. have genes in all functional classes according to [48]. In each of the four *Strongyloides* species *(S*. *ratti*, *S*. *papillosus*, *S*. *stercoralis* and *Strongyloides venezuelensis (S*. *venezuelensis))*, we found orthologs for between 29 and 31 of the individual 49 *C*. *elegans* genes searched for. Orthologs of 28 genes are present in all four species. This is similar to the numbers described for other parasitic nematode species [48]. For the 3 proteins, *rsd-3*, *sid-1* and *sid-2* which are described in *C*. *elegans* as being vital for RNA uptake and spreading [50, 51], *Strongyloides* spp., like other parasitic species in which RNAi works, only have *rsd-3* present [48]. All four genes (*tsn-1*, *ain-1*, *vig-1* and *ain-2*) known to be present in the RISC machinery [48] which is required for siRNA function, are present within all of the *Strongyloides* genomes tested suggesting that the genus as whole may be susceptible to RNAi. *Strongyloides* spp. also appear to have lost most of the *mut* family of genes with *mut-2* only present in *S*. *stercoralis*. The loss of these genes, particularly *mut-16* may explain why the 26G RNAs have not been observed within *Strongyloides* [49, 52]. Full sequences for all of the RNAi proteins in *Strongyloides* can be found in S1 Table and S1 Fig.

**Table 1:** Presence of orthologs of genes known to be involved in RNAi in *C*. *elegans* in *Strongyloides* spp.

	Small RNA biosynthetic proteins									dsRNA uptake and spreading and siRNA amplification effectors										
	*drh-3*	*drsh-1*	*xpo-1*	*xpo-2*	*dcr-1*	*drh-1*	*pash-1*	*rde-4*	*xpo-3*	*smg-2*	*smg-6*	*ego-1*	*rrf-3*	*rrf-1*	*rsd-2*	*rsd-3*	*sid-1*	*sid-2*	*rsd-6*	*smg-5*
**SRAE**	X	W	W	W	W	X	W	W		W	W	Y	W	Y		W				
**SPAL**	X	W	W	W	W	X	W	W		W	W	Y	W	Y		W				
**SSTP**	X	W	W	W	W	X	W	W		W	W	W	W	W		W				
**SVE**	X	W	W	W	W	X	W	W		W	W	W	W	W		W				
	**Nuclear RNAi effectors**														
	*mut-7*	*cid-1*	*ekl1*	*afl-1*	*mes-2*	*ekl-4*	*mes-6*	*rha-1*	*ekl-6*	*zfp-1*	*mut-2*	*ekl-5*	*mes-3*	*mut-16*	*rde-2*
**SRAE**			W	Y	W		W	W	W	W					
**SPAL**			W	Y	Y	W	W	W	W	W					
**SSTP**			W	Y	Y	W	W	W	W	W	W				
**SVE**			W	W	W	W	W	W	W	W					
	**RISC proteins**				**RNAi inhibitors**									
	*tsn-1*	*ain-1*	*vig-1*	*ain-2*	*eri-1*	*xrn-2*	*adr-2*	*xrn-1*	*adr-1*	*lin-15b*	*eri-5*	*eri-7*	*eri-3*	*eri-6*
**SRAE**	W	Z	W	Z	W	Z		Z				W		
**SPAL**	W	Z	W	Z	W	W		W			W	W		
**SSTP**	W	Z	W	Z	W	Z		Z				W		
**SVE**	W	Z	W	Z	W	Z		Z				W		

Proteins are split into 5 groups based on function as in [48]. W—likely one to one ortholog with the *C*. *elegans gene*. X—many to one ortholog with multiple *C*. *elegans* genes having a single ortholog in *Strongyloides*. Y—one to many ortholog with a single *C*. *elegans* gene having multiple orthologs in *Strongyloides*. Z—many to many ortholog between *C*. *elegans* and *Strongyloides*. Species key–SRAE (*S*. *ratti)*, SPAL (*S*. *papillosus*), SSTP (*S*. *stercoralis)* and SVE (*S*. *venezuelensis)*. The full amino acid sequences for each of the DAF-12 proteins can be found in S1 Fig.

### *Strongyloides* spp. have a *daf-12* one to one ortholog

**Fig 1:**
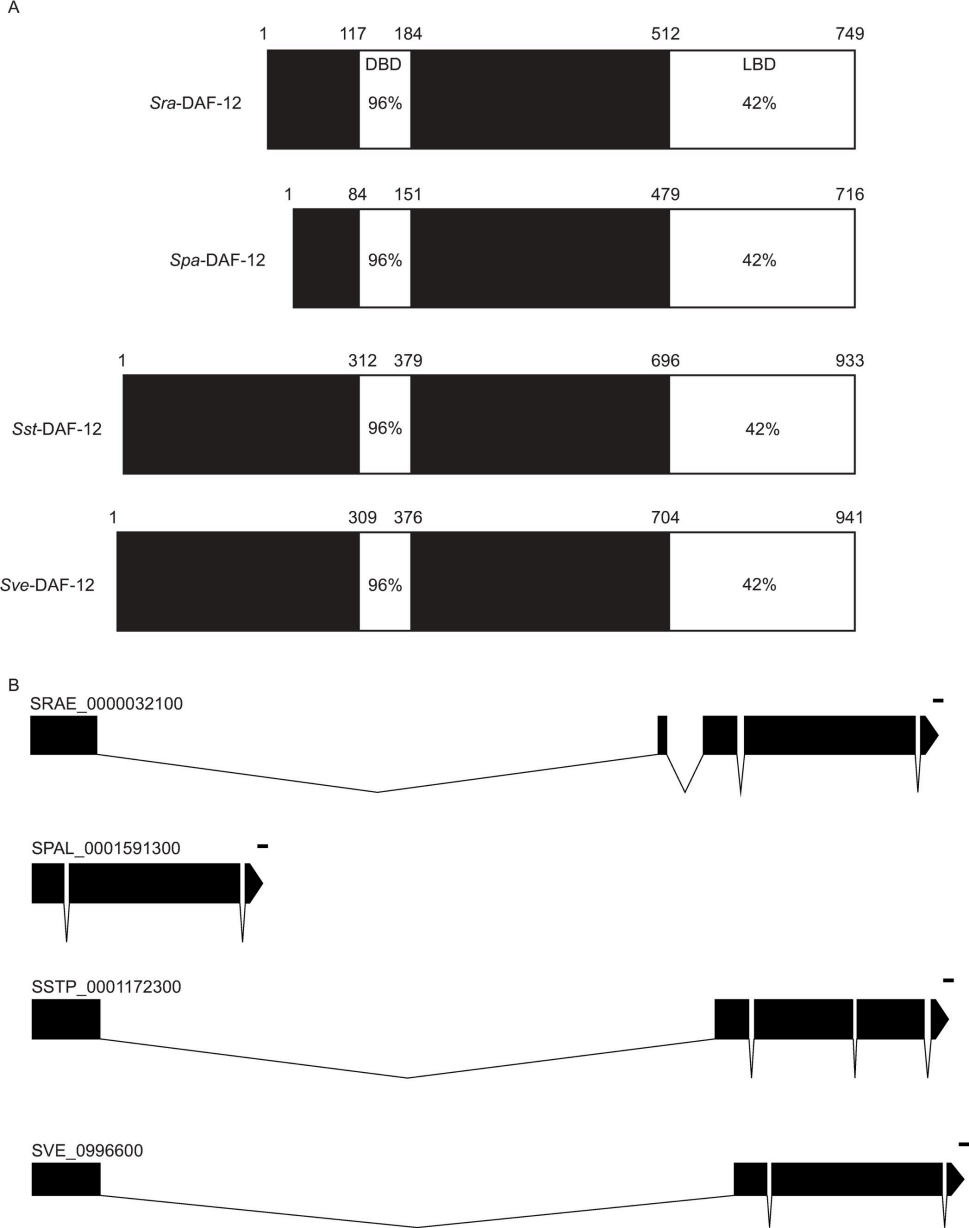
*daf-12* homologs in four species of *Strongyloides*. A) Schematic representation of the DAF-12 proteins. Amino acid identities with *C*. *elegans* are given for the DNA binding domain (DBD) and for the ligand binding domain (LBD). The species prefixes are *Sra S*. *ratti*, *Spa S*. *papillosus*, *Sst S*. *stercoralis* and *Sve S*. *venezuelensis*. B) Gene models of the *daf-12* homologs in *Strongyloides*. spp. Notice that, *daf-12* within *S*. *ratti*, *S*. *stercoralis* and *S*. *venezuelensis* all have a large intron between their first and second exons unlike *S*. *papillosus* which lacks this feature. However the size of the *S*. *papillosus* protein (719 amino acids) is still similar to that of *S*. *ratti* (749 amino acids). The labels are the gene identifiers according to WormBase ParaSite (https://parasite.wormbase.org/index.html) containing the species codes SRAE *S*. *ratti*, SPAL *S*. *papillosus*, SSTP *S*. *stercoralis* and SVE *S*. *venezuelensis*. Scale bar = 100bp.

We used both the whole *C*. *elegans daf-12* gene, and the heavily conserved DNA-binding domain [27, 53, 54] for tblastn/blastp searching the available *Strongyloides* spp. genomes [34]. In each of the four species we identified exactly one gene containing both the DNA binding domain (DBD) and the ligand binding domain (LBD) (SRAE_0000032100 [*S*. *ratti*] (e-value 2.1E-99 compared to 5.4E-30 for 2^nd^ best gene), SPAL_0001591300 [*S*. *papillosus*] (e-value 9.0E-97), SSTP_0001172300 [*S*. *stercoralis*] (e-value 4.2E-95) and SVE_0996600 [*S*. *venezuelensis*] (e-value 4.6E-93) (full sequences in S2 Fig). In reverse BLAST searches all these sequences identified *daf-12* as their closest *C*. *elegans* homolog with a large margin (i.e. 6.3E-73 versus 1.1E-29 for the second best gene for *S*. *ratti*). The *S*. *stercoralis* gene identified is the gene previously described by [27]. In the DBD, the amino acid sequences of the four *Strongyloides* spp. genes are extremely highly conserved among themselves (100% identity) and compared with *C*. *elegans* (96% identity). The LBDs of the *Strongyloides* genes are also very similar (>99% identity) but, the conservation compared with *C*. *elegans* is lower (42% identity) (Fig 1A). From this we concluded that the *Strongyloides* genes and *C*. *elegans daf-12* are most likely one to one orthologs and we proceeded to knock down SRAE_0000032100 in *S*. *ratti*. From here on, we will refer to this gene as *Sra-daf-12*.

### RNAi mediated suppression of genes within *S*. *ratti*

In order to study the function of *daf-12* natively within *S*. *ratti*, *Sra-daf-12* was disrupted by RNAi soaking. To this end, first a protocol had to be devised in order to get RNAi to work within *S*. *ratti*. Based upon prior knowledge gained from profiling the RNAi pathway proteins, we developed a protocol for RNAi by soaking using siRNAs starting from a published protocol for *Brugia malayi [45]*, another parasitic but phylogenetically distant nematode. By systematically varying the soaking medium, duration of soaking, addition of pharyngeal pump-inducing compounds, age of larvae used in experiment and soaking temperature, we developed the two protocols (“Early-stage” and “Late-stage”) described in Materials and Methods.

**Fig 2:**
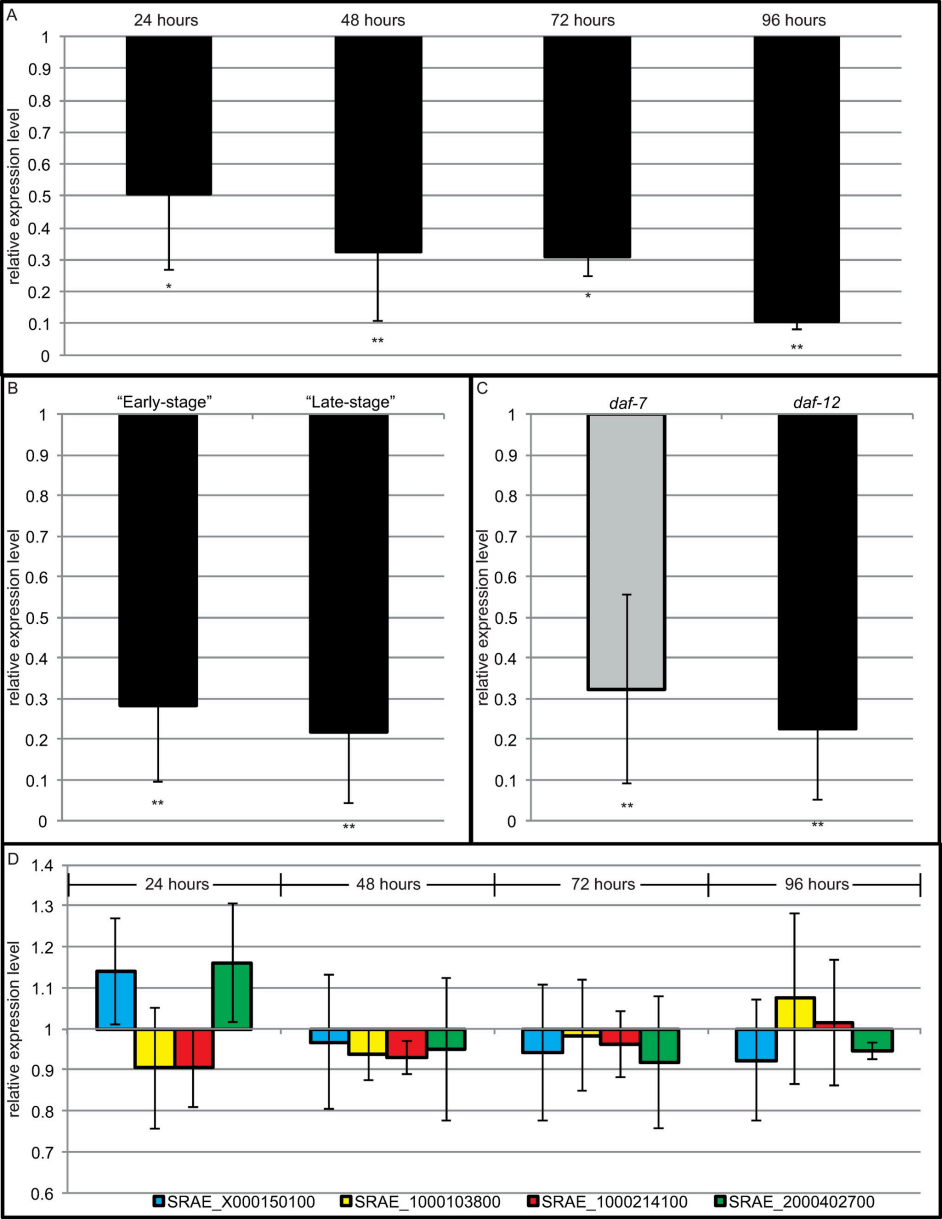
RNAi-mediated suppression within *Strongyloides ratti*. RNAi reduces expression of *daf-12* with statistically significantly increased reduction in *daf-*12 as length of soaking increases (relative gene expression of 0.51 ± 0.24 after 24 hours (p = 0.01), 0.32 ± 0.22 after 48 hours (p<0.001), 0.31 ± 0.06 after 72 hours (p = 0.01) and 0.11 ± 0.03 after 96 hours (p<0.001) (A), with both the “Early-stage” (relative gene expression 0.28 ± 0.19 (p<0.001)) and “Late-stage” soaking protocols (relative gene expression 0.22 ± 0.18 (p<0.001)) (B) resulting in a reduction following 48 hours of soaking. The “Late-stage” technique can be to achieve a statistically significant reduction in expression in both *daf-7* (in grey) (relative gene expression 0.32 ± 0.23 (p<0.001)) and *daf-12* (in black) (0.23 ± 0.17 (p<0.001)) (C), with minimal off-target effects detected across five genes with a similarity to an siRNA targeting *daf-12* in *S*. *ratti* over different time points (relative expression of SRAE_X000150100 was 1.14 ± 0.13 after 24 hours, 0.97 ± 0.16 after 48 hours, 0.94 ± 0.17 after 72 hours and 0.92 ± 0.15 after 96 hours. Relative expression of SRAE_1000103800 was 0.90 ± 0.15 after 24 hours, 0.94 ± 0.06 after 48 hours, 0.98 ± 0.14 after 72 hours and 1.07 ± 0.21 after 96 hours. Relative expression of SRAE_1000214100 was 0.94 ± 0.17 after 24 hours, 0.98 ± 0.14 after 48 hours, 0.96 ± 0.08 after 72 hours and 0.92 ± 0.16 after 96 hours. Relative expression of SRAE_2000402700 was 0.92 ± 0.15 after 24 hours, 1.07 ± 0.21 after 48 hours, 1.02 ± 0.15 after 72 hours and 0.95 ± 0.02 after 96 hours (no statistically significant reduction in any of genes at any of the time points)) (D). *S*. *ratti* larvae were soaked using either the “Early-stage” protocol (B) or “Late-stage” protocol (A-D) and then the relative expression level of the targeted gene was determined using qRT-PCR compared to 3 control genes with a scrambled siRNA used as a control. Error bars represent standard deviation. These figures show the mean value comprised of a minimum of 3 biological replicates with 3 technical replicates per biological replicate. Mann Whitney U analysis was performed to determine statistical significance. * indicates a statistically significant difference (p-value ≤0.01 - ≥0.001), ** indicates a highly statistically significant difference (p-value ≤0.001).

To determine if soaking length had a significant effect upon RNAi, larvae were soaked using the “Late-stage” protocol for upto 4 days, with the relative expression of *daf-12* determined every 24 hours. As seen in Fig 2A, 48 hours is sufficient time to achieve a statistically significant reduction of over two-thirds in expression (relative expression level 0.31 ± 0.06 (p<0.0001)). As a result, all experiments in this paper used a minimum soaking time of 48 hours and upto a maximum of 96 hours.

As seen in Fig 2B, expression of *daf-12* could be reduced strongly using both the “Early-stage” (0.28 ± 0.19 (p<0.0001)) and “Late-stage” (0.22 ± 0.18 (p<0.0001)) soaking protocols. Decreased expression of *daf-12* in RNAi treated worms compared with worms treated with a scrambled siRNA (control) was determined by qRT-PCR. This suggests that the method is effective across different life stages meaning genes of interest can be studied across the entire free living part of the life cycle.

Following successful RNAi of *daf-12*, to further evaluate the method we knocked down a further two genes (*msp*[major sperm protein] and *daf-7*) using the “Late-stage” soaking protocol and compared by qRT-PCR the respective mRNA in soaked larvae treated with either a targeted siRNA or a scrambled siRNA (control). MSP is actually encoded by several very similar genes, all of which contain the targeted sequence element. For normalization we used the reference genes *tbb-1*, *rpl-37* and *gpd-2* [55]. The list of siRNA sequences used in this study can be found in Table 2 and the list of primers used can be found in S2 Table. As seen in Fig 2C, expression of the targeted gene was consistently decreased in both *daf-12* (0.22 ± 0.17 (p<0.0001)) and *daf-7* (0.31 ± 0.23 (p<0.001)). Expression could not be determined for *msp* because of the complete death of all worms and offspring treated with a *msp*-targeting siRNA. Based on *C*. *elegans msp* loss of function this lethality was actually the expected phenotype [56]. The fact that our method worked for three out of three cases tested confirms that the method is robust and functions across a wider-range of genes.

**Table 2:** List of siRNA sequences used in this study.

Targeted Gene		Sequence (5’ to 3’)
*daf-12*	sense	(UU) AGTTGATGGTCATTCACAA
	antisense	(UU) UUGUGAAUGACCAUCAACU
*msp*	sense	(UU) GACCUUCAGACGUGAAUGG
	antisense	(UU) CCAUUCACGUCUGAAGGUC
*daf-7*	sense	(UU) UGAAAUGGUACAGACAAAU
	antisense	(UU) AUUUGUCUGUACCAUUUCA
Scrambled siRNA (negative control)	sense	(UU) AGGUAGUGUAAUCGCCUUG
	antisense	(UU) CAAGGCGAUUACACUACCU

Finally, to determine off-target effects, all genes containing a sequence of 13 nucleotides or more identical to the siRNA had their relative expression level determined (Fig 2D). As seen in Fig 2D, minimal inconsistent changes in the mRNA levels of these genes were seen with either of the siRNAs. This suggests that the knock down was specific and that the phenotypic changes seen in the treated worms are unlikely to be the result of off-target effects but rather are the consequence of the knockdown of the gene of interest.

This flexibility in soaking stage, lack of off-target effects and the ability of study a wide variety of genes, makes RNAi potentially a highly suitable and effective tool for research in *S*. *ratti*.

### Suppression of *daf-12* prevents direct development to L3i

**Fig 3:**
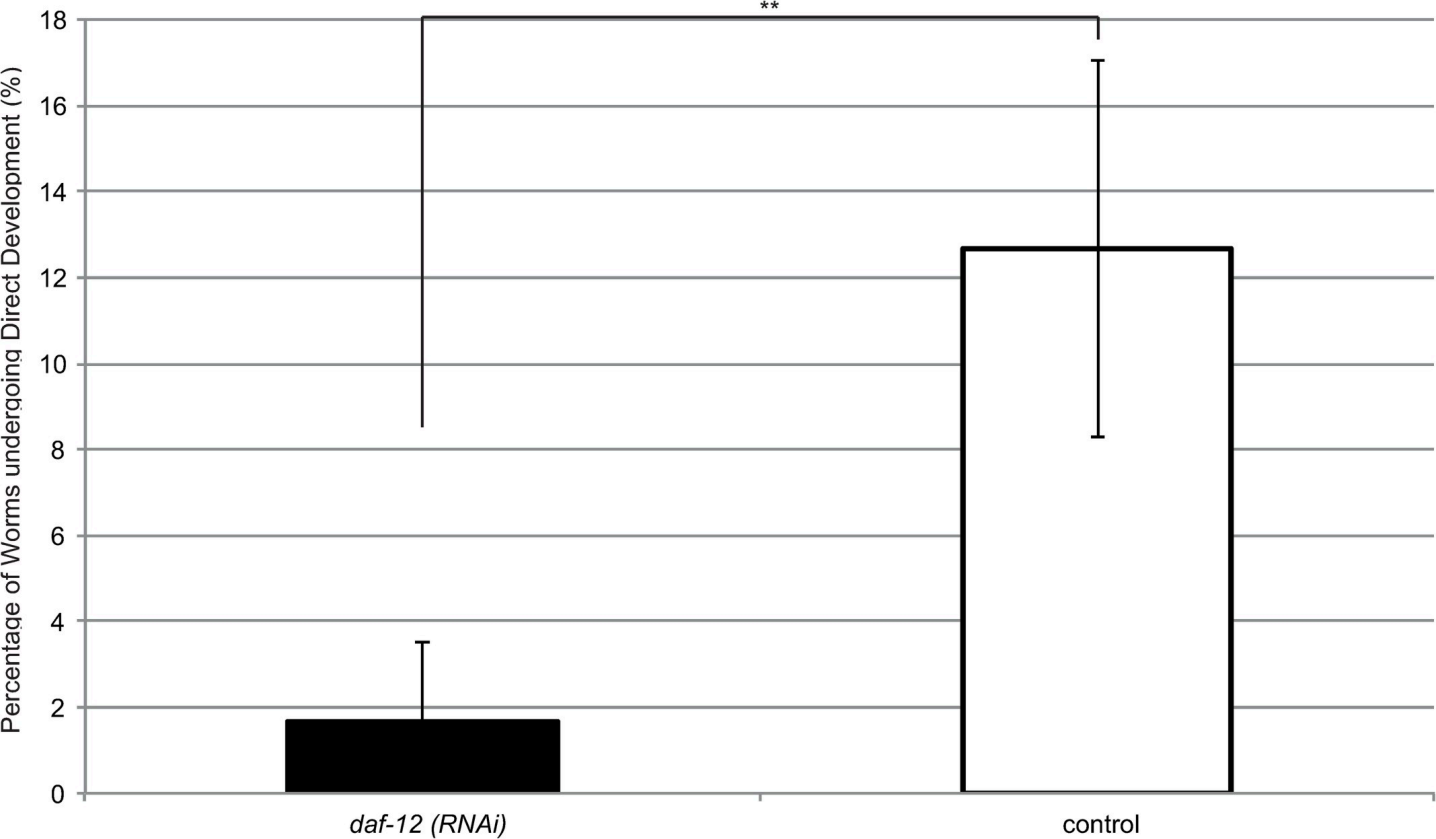
Suppression of *daf-12* significantly reduces the ability of worms to undergo direct development to infective larvae. Young worms were soaked with an siRNA targeting *daf-12 (daf-12(RNAi))* or a scrambled siRNA (control), and the proportion of females which developed to infective larvae after 3 days was determined. Males were excluded from this analysis as they cannot undergo direct development. Error bars represent standard deviation. This figure shows the mean value comprised from 3 biological replicates with 4 technical replicates per biological replicate. Mann Whitney U analysis was performed to determine statistical analysis. ** indicates a highly statistically significant p-value of less than 0.001 (p<0.0001).

As outlined in the introduction, when female larvae exit the host in *Strongyloides*, they have a choice between either undergoing direct development and developing into L3i, or alternatively undergoing indirect development with the aforementioned sexual reproduction outside of the host. The exact proportion of larvae which undergo direct development depends on both the strain and species of *Strongyloides* as well as environmental factors such as the host immune status and external conditions. For two species of *Strongyloides* (*S*. *stercoralis* [27] and *S*. *papillosus* [5]) treatment with DA prevented the formation of L3i and re-directed the development towards the indirect cycle. If DA indeed acted by inactivating the L3i promoting action of *Sra*-DAF-12, knocking down *Sra-daf-12* should result in the suppression of L3i formation. To test this young L1 larvae were treated with siRNA ("Early-stage", full details in methods) and the number which then underwent direct development were counted and compared to those which underwent normal development. *daf-12(RNAi)* larvae were highly statistically significantly impaired in their ability to form direct L3i (Fig 3) with only 1.67% ± 0.94 undergoing direct development compared to 12.67% ± 2.19 in the control (p<0.0001).

### Suppression of *daf-12* has a minor effect on fecundity in *S*. *ratti*

To determine whether *Sra-daf-12* would affect fecundity, as is the case in *C*. *elegans* [19, 22], we knocked down the gene following the "Late-stage" procedure (see above and Materials and Methods).

**Fig 4:**
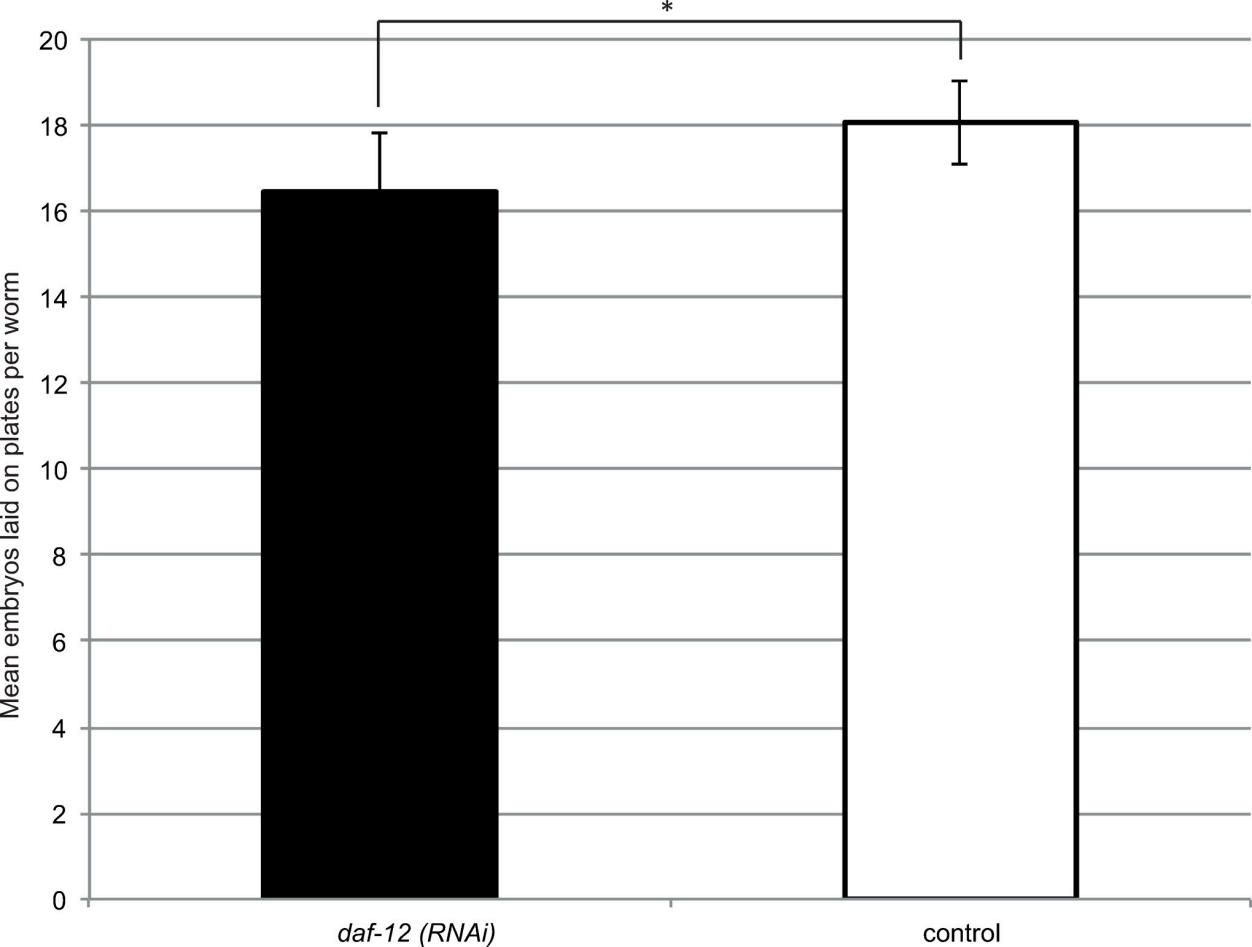
*daf-12(RNAi)* free-living adults lay slightly but significantly less embryos than control adults. Following soaking (“Late-stage” protocol) with either a scrambled siRNA (control) or *daf-12* targeting siRNA *(daf-12(RNAi))*, free living adults were recovered on plates and the number of embryos they laid counted. Error bars represent standard deviation. This experiment was repeated three times. The bars represent a mean calculated from all three biological replicates. Student’s t-test was performed to determine statistical significance. * indicates a statistically significant p-value between 0.01 and 0.001 (p = 0.004).

Adults which had been soaked in *daf-12* targeting siRNAs (here on referred to as “*daf-12(RNAi)*”) had a small but statistically significant reduction in number of eggs laid (18.04 ± 1.40 per worm in control to 16.45 ± 0.96 in *daf-12(RNAi*), p = 0.004) as seen in Fig 4. This suggests that *daf-12* in *S*. *ratti* may be involved in either reproductive development or alternatively in the actual laying procedure. Examination of the vulva of 10 random individuals from both treatments using high magnification (100X) DIC microscopy revealed no structural defects in the egg laying machinery. Further, we found no difference in the number of eggs still present in the worm (7.90 ± 1.10 in *daf-12(RNAi)* versus 7.50 ± 1.27 in control, p = 0.46), suggesting that the observed difference was due to a slightly smaller number of eggs produced rather than problems with egg laying.

### *daf-12(RNAi)* progeny of free-living worms are still able to develop into L3i albeit more slowly and in reduced numbers

As DA interfered with the development of the progeny of free-living adults to L3i in two other species of *Strongyloides* [5, 26, 27], we asked if this was also the case in *daf-12(RNAi)*. The development of progeny of "Late-stage" adults (the larvae remained in RNA solution throughout development) was recorded every 24 hours over a period of 5 days post-treatment. As can be seen in Table 3, *daf-12(RNAi)* larvae were still able to develop into L3i but did so more slowly with an apparent 24 hour delay in development compared to control worms. It took 3 days post-laying until the majority of the *daf-12(RNAi)* larvae that did develop all the way through to L3i had reached this developmental stage while more than 65% of the control larvae that developed to L3i had done so after two days. Further, statistically significantly less *daf-12(RNAi)* larvae compared to control larvae successfully completed the developed to L3i (75.36% ± 13.09 versus 91.55% ± 5.59, p<0.0001). Taken together these results suggest that *daf-12* does have a role in the development of the progeny of the free-living generation to infective larvae within *S*. *ratti*.

**Table 3:** *daf-12(RNAi)* L3i develop more slowly and in fewer numbers than control L3i.

	Day 1	Day 2	Day 3	Day 4	Day 5
***daf-12 (RNAi)* L3i on each day (%)**	7.41 ± 3.97	6.17 ± 3.48	39.37 ± 11.84	19.91 ± 5.76	2.49 ± 2.00
***daf-12 (RNAi)* L3i cumulative (%)**	7.41 ± 3.97 (ns)	13.58 ± 6.28 (**)	52.95 ± 5.15 (**)	72.87 ± 12.78 (**)	75.36 ± 13.09 (**)
**Control L3i on each day (%)**	10.13 ± 1.77	54.75 ± 5.15	17.77 ± 4.72	7.69 ± 3.46	1.21 ± 1.50
**Control L3i cumulative (%)**	10.13 ± 1.77	64.88 ± 4.90	82.65 ± 7.73	90.33 ± 5.80	91.55 ± 5.59

% of larvae, which had developed to L3i at the day indicated. The numbers shown are the means of three independent replicates plus/minus the standard deviation. Per replicate at least 180 individual larvae were followed. Statistical analysis (Mann-Whitney U) to compare *daf-12 (RNAi)* and control larvae cumulatively every 24 hours was performed. ns indicates a not statistically significant value (p-value >0.01), * indicates a statistically significantly value (p-value between 0.01 and 0.001), ** indicates a highly statistically significant value (p-value <0.001)

### *daf-12(RNAi)* infective larvae are still able to infect rats

**Fig 5:**
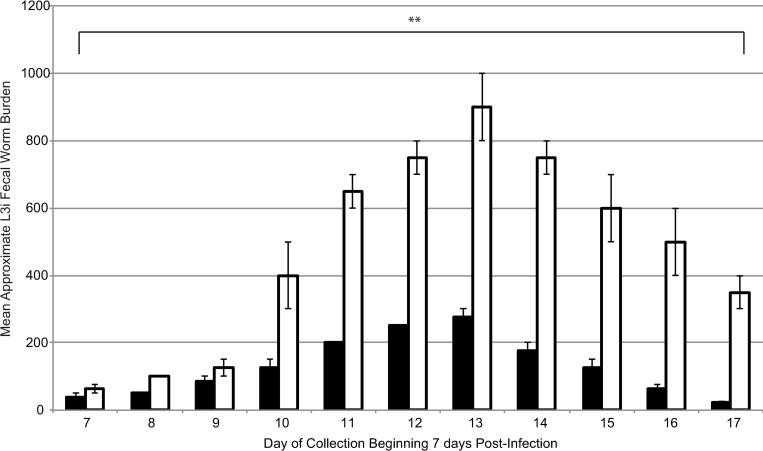
The *daf-12(RNAi)* larvae that do develop into L3i (black) are able to infect rats when injected subcutaneously but cause significantly less productive infections than control (scrambled siRNA) L3i (white). Approximate worm burden in the feces (as a proxi for the capacity of causing a new infection) was determined by counting the L3i present in fecal cultures after 7 days incubation at 19°C. Fecal cultures were collected daily starting from 7 days post-infection. Error bars represent standard deviation. This experiment was carried out twice. Student’s t-test was performed to determine statistical significance. ** indicates a highly statistically significant difference (p-value <0.001) between the *daf-12(RNAi)* and control infections (p<0.0001).

In order to test if the *daf-12(RNAi)* larvae that were successful in developing into morphologically normal looking L3i were functional and able to establish an infection in a new host, 100 *daf-12(RNAi)* L3i were injected subcutaneously into a rat and the infection allowed to develop for 7 days, after which the feces was collected daily and incubated at 19°C for 7 days to allow for new L3i to develop. These L3i were counted and used as a measure for the productivity of the infection. As a control, 100 L3i which had been treated using a scrambled siRNA were also injected subcutaneously into a separate rat and the same procedures followed (full details in methods, two independent replicates were done). As can be seen in Fig 5, in two independent experiments *daf-12(RNAi)* L3i (shown in black) were still able to develop an infection within a rat, however one with a statistically significantly reduced productivity compared to the control L3i (p<0.0001). Knockdown of *daf-12* caused a reduced peak in worm production (average of 275 larvae after 7 days compared to 900 in the control) and the length of infection was also slightly reduced, with no worms being seen in fecal cultures after 17 days post-infection compared to control larvae infection in which worms could still be seen on 19 days post-infection.

### Suppression of *daf-12* affects metabolism within *S*. *ratti*

**Fig 6:**
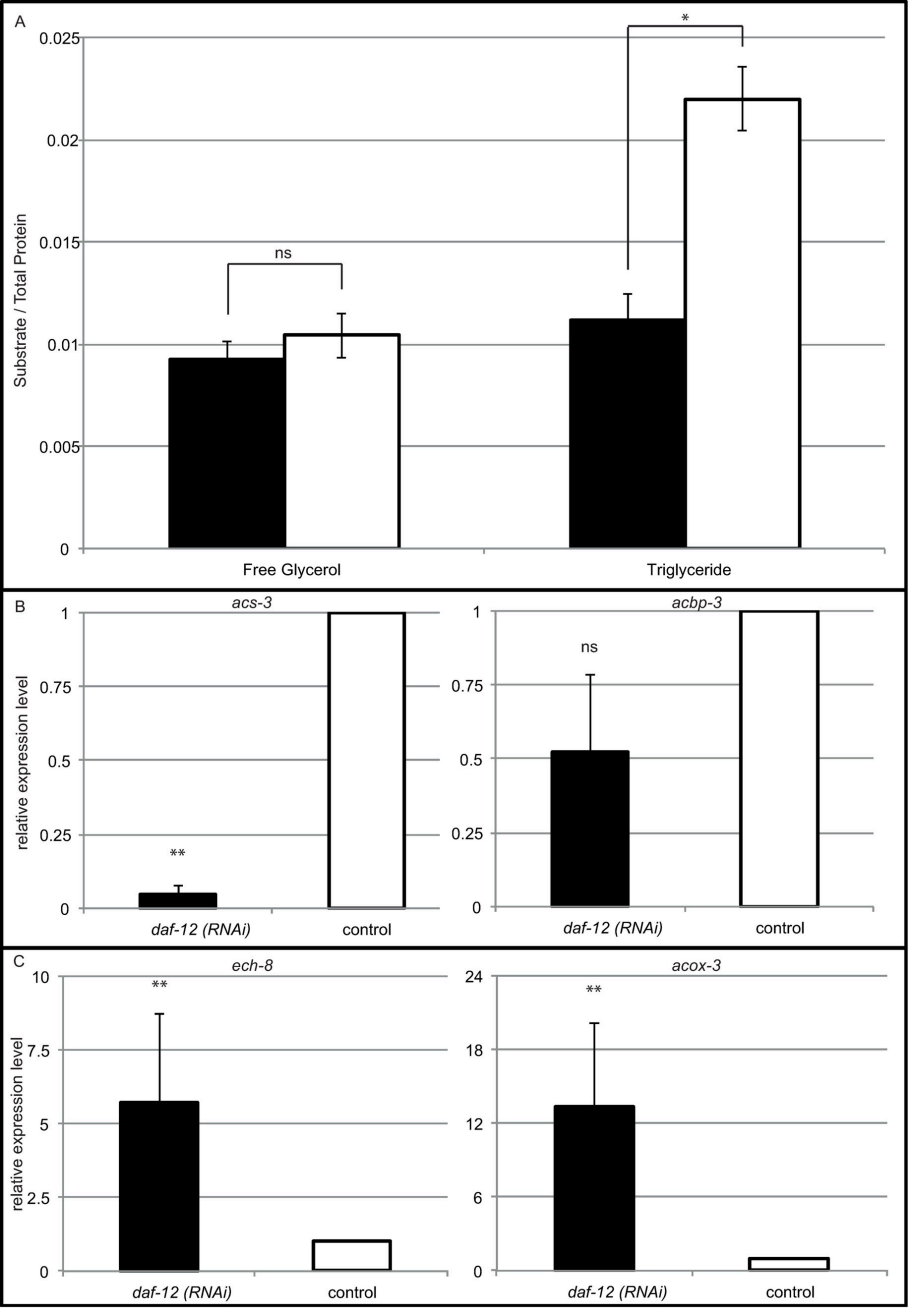
Suppression of *daf-12* within *Strongyloides ratti* results in multiple changes in metabolism. Triglyceride content is statistically significantly reduced in *daf-12(RNAi)* larvae (black) than control larvae (white) following “Late-stage” soaking (A) (p-value <0.001). Similarly, the aerobic metabolism genes *acs-3* (p<0.001) and *acbp-3* (p = 0.057) have decreased expression in *daf-12 (RNAi)* larvae (B), while the anaerobic metabolism genes *ech-8* (p = 0.001) and *acox-3* (p<0.0001) are upregulated in *daf-12 (RNAi)* (C). To determine fatty acid stores, total triglyceride and free glycerol were measured from worm lysates and are plotted against total protein in the sample. This experiment was performed twice. To determine changes in gene expression, control larvae expression was normalized as 1 and fold change was calculated using the ΔΔCt method (see Materials and Methods) to determine change in expression of the genes of interest. Fold change shown is the mean fold change of the gene of interest compared to 3 control genes *(tbb-1*, *gpd-2*, *rpl-37* [55]) taken from at least 3 biological replicates with each biological replicate consisting of 3 technical replicates. Error bars represent standard deviation. Mann Whitney U analysis was performed to determine statistical significance. ns indicates a not statistically significant difference (p-value >0.01), * indicates a statistically significant difference (p-value ≤0.01 - ≥0.001), ** indicates a highly statistically significant difference (p-value ≤0.001).

In *C*. *elegans*, DA application leads to increased aerobic fat metabolism [16]. In the same publication the authors also demonstrated that exogenous DA lead to an increase in expression of genes involved in aerobic triglyceride metabolism and a lower expression of genes involved in anaerobic metabolism in *S*. *stercoralis*. To determine whether *Sra-daf-12* is involved in fat utilization, levels of triglycerides stored within the worms were determined and compared to levels of free glycerol (Fig 6A). Whilst free glycerol levels were similar between both *daf-12(RNAi)* and control larvae, there was a statistically significant reduction in triglycerides stored in *daf-12(RNAi)* larvae (0.011 ± 0.001 compared to 0.022 ± 0.002 in control, p<0.0001). To further examine this potential shift between aerobic and anaerobic metabolism, two genes known to be involved in aerobic metabolism (*acs-3* and *acbp-3*) and two genes known to be involved in anaerobic metabolism (*ech-8* and *acox-3*) had their relative expression levels determined by qRT-PCR (Fig 6B and 6C). *acs-3* was strongly statistically significantly downregulated (to 0.047 ± 0.029 compared to the control, p<0.001). For *acbp-3* we measured a reduction to 0.523 ± 0.259, but this was not statistically significant (p = 0.057). Similarly, both anaerobic metabolism genes were highly significantly upregulated in *daf-12(RNAi)* larvae (Fig 6C) compared to the control (5.719 ± 3.006 to 1 enrichment in *ech-8* (p = 0.001) and 13.349 ± 6.776 to 1 in *acox-3* (p<0.001)). These results, together with [16] strongly suggest that in *Strongyloides* spp., as in *C*. *elegans*, ligand bound DAF-12 promotes aerobic and suppresses anaerobic metabolism.

### *daf-12(RNAi)* are less tolerant to heat stress

**Fig 7:**
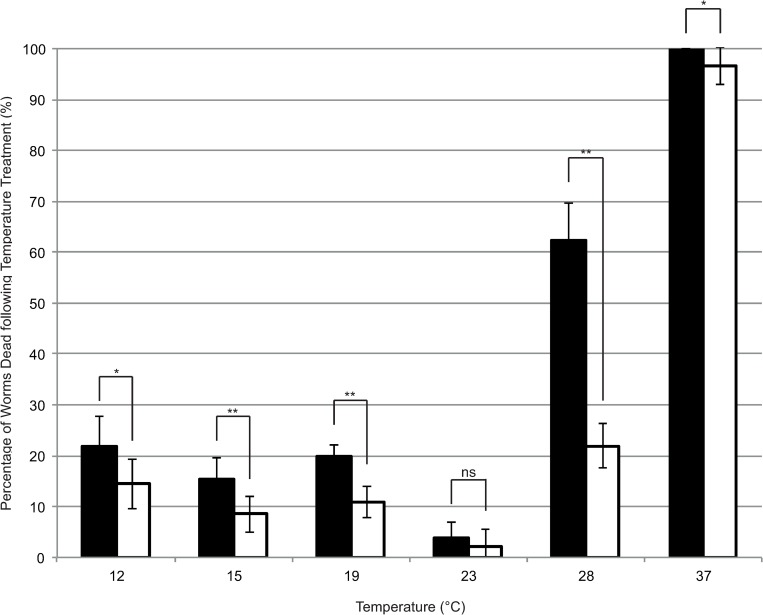
*daf-12(RNAi)* worms (black) have decreased tolerance to temperature stress compared to control worms (white). L1 larvae were soaked with a scrambled siRNA (control) or one targeting *daf-12 (daf-12(RNAi))* and were then exposed to different temperatures for 4 hours, after which their survival was determined. Worms maintained at 12 and 37°C both had a statistically significant increase in death following temperature stress (p = 0.003 and p = 0.005). Worms maintained at 15, 19 and 28°C had a highly statistically significant increase in death following temperature stress (all p<0.001) whereas worms maintained at 23°C had no statistically significant difference in death (p = 0.083). Error bars represent standard deviation. The bars represent the mean percentage of worms dead post-heat shock, calculated from three biological replicates, each containing four technical replicates. Mann Whitney U analysis was performed to determine statistical significance. ns indicates a non-statistically significant difference (p-value >0.01), * indicates a statistically significant difference (p-value ≤0.01 - ≥0.001), ** indicates a highly statistically significant difference (p-value ≤0.001).

Different *daf-12* mutations are known to result in different responses to heat stress in *C*. *elegans* [22]. To test whether *daf-12* also played a role in thermotolerance in *S*. *ratti*, *daf-12(RNAi)* and control worms were subjected to different temperatures between 12°C and 37°C 48 hours after initiating soaking at 19°C (“Early-stage” Protocol). The percentage that perished was used as an indicator of their tolerance to these temperatures (Fig 7). *daf-12(RNAi)* worms were significantly less tolerant to all temperatures outside of their optimum (23°C) compared to control. Also at colder temperatures there was a statistically significantly increase in nematode death between *daf-12(RNAi)* and control (21.83% ± 6.00 in *daf-12(RNAi)* at 12°C compared to 14.50% ± 4.91 (p = 0.003)). Interestingly at 19°C, the survival was lower than at 23°C despite the fact that *S*. *ratti* isolate used had normally been maintained at 19°C and that the soaking experiment had been initiated at 19°C. At 28°C *daf-12(RNAi)* worms had a statistically highly significantly reduced survival rate (62.33% ± 7.38 dead in *daf-12(RNAi)* versus 21.92% ± 4.40 in control (p<0.001)). At 37°C, the heat treatment was nearly completely lethal for both nematode populations, yet still a few control worms (3.33%) but no *daf-12(RNAi)* worms survived. These results suggest that *daf-12* is involved in thermotolerance within *S*. *ratti* and might be involved in reacting to changes in their environment.

## Discussion

### RNAi in *S*. *ratti*

We found *Strongyloides* spp. to have homologs of many but not all *C*. *elegans* genes known to be involved in RNAi as listed by [48]. The fact that we found essentially the same reduced set of genes in all four species of *Strongyloides* examined suggests that the reduction in gene number is real and not the consequence of incompleteness of the *Strongyloides* spp. genome assemblies. The set of putative RNAi genes present in *Strongyloides* spp. is similar to those reported in [48] for other parasitic nematodes in which RNAi works. The presence of all of the RISC protein genes (*tsn-1*, *ain-1*, *vig-1 and ain-2*) and the absence of genes such as *rsd-6*, *sid-1* or *sid-2*, all of which are known to be involved in dsRNA uptake, may explain why, for successful RNAi, we had to apply siRNAs rather than long double stranded RNAs as it is common in *C*. *elegans*.

Of interest is the lack of *eri*-family genes within *Strongyloides* spp. with only *eri-1* and *eri-7* present in all four genomes. These genes are known to be enhancers of RNAi [57]. The loss of *mut* genes, in particular *mut-16* in *Strongyloides* spp. may explain why the 26G RNAs were not observed within Strongyloididae [49, 52].

Based on protocols described for *B*. *malayi*, we developed RNAi for *S*. *ratti* and demonstrated that it works for three different genes and across several different life stages. Given that three separate genes have been successfully suppressed with minimal off-target effects, we are convinced that with this we can add a robust procedure for gene knock down to the current toolbox in *S*. *ratti*. Although this toolbox has recently grown considerably [34, 35] we think our addition is substantial. Obviously true mutations (for example those generated by CRISPR/Cas9) do have advantages and are in many cases desirable. However, having the option of knocking down gene functions by RNAi is very useful, in particular in *S*. *ratti*, where CRISPR/Cas9 works much less efficiently than in *S*. *stercoralis* (35). *S*. *ratti* is an important model species, which, contrary to *S*. *stercoralis*, can be maintained easily in the laboratory in a natural rodent host. Further, every mutant line must be maintained and this, for an obligatory parasite like *S*. *ratti*, requires a substantial number of laboratory animals and is laborious and expensive. We showed that our protocol allows the analysis of gene functions at different stages of the part of the life cycle that occurs outside of the host without the need of a host passage. How long the RNAi effect persists in larvae after the infection of a host remains to be determined.

### Phenotypic analysis of *daf-12(RNAi)* in *S*. *ratti*

*daf-12* knockdown severely inhibited the formation of L3i of the direct cycle as has been described for DA in *S*. *papillosus* [5] and *S*. *stercoralis* [27]. However, the suppression of *daf-12* did not cause the progeny of the free-living generation to form a second free-living generation as it had been observed upon DA addition in *S*. *papillosus* [5]. Our results are more comparable with the DA results in *S*. *stercoralis* where the switch of the developmental trajectory was less complete. We found, that *daf-12(RNAi)* progeny of the free-living generation develop slower and in part never complete their differentiation to L3i and those that do reach this stage, although still capable of infecting a host, cause less productive infections. These findings may, but not necessarily do, reflect a partial redirection of development towards non-parasitic L3. It is likely that it is much more difficult to redirect progeny of the free-living generation, which normally all develop into L3i, towards free-living than the progeny of the parasitic generation for which the switch between the two life cycles is normal. As expected, we found *Sra-daf-12* to be involved in the switch between aerobic and anaerobic metabolism. It should be noticed that the effect of knocking down *daf-12* was the same as DA treatment as far as the direct—indirect life cycle switch is concerned but the opposite with respect to the control of genes associated with aerobic to anaerobic metabolism. This confirms that the promotion of the dauer/L3i development is a function of the ligand free- DAF-12 and is inhibited by ligand binding and hence, exogenous DA [5, 27] or depletion of DAF-12 lead to the absence of L3i. On the other hand, promoting aerobic fat usage is achieved by the ligand bound receptor and is therefore enhanced by DA addition [16] but inhibited by *daf-12(RNAi)*. The loss of thermotolerance is also of high importance as these larvae are required by their life cycle to survive and reproduce within the wild at a wide variety of temperatures.

With the RNAi protocol presented, we added a new tool to study gene function in *S*. *ratti*. Using this tool we showed that *daf-12* is an important gene in *S*. *ratti* for the control of essentially the same various developmental and metabolic processes as in *C*. *elegans*. Our results are in full agreement with the hypothesis that the pharmacological effects caused by DA in *Strongyloides* spp. described before [5, 16, 26, 27] involved DAF-12, as suspected but not demonstrated by the authors of these publications and argue for a highly conserved function of *daf-12* in various, only distantly related nematodes. This supports that indeed *daf-12* is a viable target for the development of new target specific antihelminthic drugs. The experiments with DA [23, 27] showed that it is possible to change the developmental pathway of *Strongyloide*s spp. larvae away from differentiating into infective larvae by the administration of small organic molecules (dafachronic acid) both, in and outside of the host. Based on the knowledge of the structure of *S*. *stercoralis* DAF-12, other inhibitory molecules with optimized pharmacological properties can now be developed.

## Materials and methods

### Ethics statement

Animal care and use adhered to the German Animal Protection Law (Tierschutzgesetz), the German Animal Protection Laboratory Animal Ordinance (Tierschutz-Versuchstierverordnung) and EU Directive 2010/63/EU on the protection of animals used for scientific purposes. The procedures for animal maintenance and experiments were ethically and administratively approved by the local govermental authorities in charge (Regierungspräsidium Tübingen), who also issued the necessary permits (AZ35/9185.82–5). The animals were kept in an in-house facility, which is regularly inspected by the local veterinary authorities (Veterinäramt Tübingen). No experiments on human subjects were conducted in this study.

### Maintenance of *Strongyloides ratti*

The laboratory strain of *Strongyloides ratti* strain ED321 [58] was used for these experiments. *S*. *ratti* was kept in our in-house animal facility in female Wistar rats (Charles River Inc). Four week old female rats were injected subcutaneously with around 800 infective larvae each. 7 days post-infection, their feces was collected by placing the infected animals overnight in a cage with a metal grid bottom, lined with wetted paper at 23°C. The following morning, the feces were collected and cultured in watch glasses in an incubator at 19°C as described in [59]. 2 days post-collection, the feces were removed from the incubator and the worms were isolated from the cultures using a Baermann funnel as described in [60]. Briefly, fecal materials was wrapped in Linsoft paper and placed in a funnel closed at the bottom with a clamp and filled with tap water, and placed back into the incubator for 2 hours. Following this, the worms that had accumulated at the bottom were removed by briefly opening the clamp and then cleaned. For the experiments that required the use of first or second stage larvae, feces were used within 12 hours of being collected and the worms then isolated the same way.

### BLAST analysis of the *Strongyloides* spp. RNAi pathway

Proteins known to be involved in RNAi in *Caenorhabditis elegans* [48] were retrieved from WormBase Parasite (wormbase.org: Version WBPS11) and used as queries for protein BLASTs (BLASTp) and translated nucleotide BLASTs (tBLASTn) against the *Strongyloides* genomes currently listed on WormBase Parasite (parasitewormbase.org: release WBPS11 (WS265): *Strongyloides ratti* (PRJEB125), *Strongyloides papillosus* (PRJEB525), *Strongyloides stercoralis* (PRJEB528), *Strongyloides venezuelensis* (PRJEB530)). To determine whether a protein was present in each Strongyloididae genome, a similar strategy to that in [48] was implemented. After aligning with BLASTp and tBLASTn against the protein of interest, only proteins with an E-value of less than 0.0001 were carried forward. The protein was then searched for in the *C*. *elegans* genome using tBLASTn and for proteins with a minimal alignment score of 40 bits and an E-value of less than 0.0001, domain structure was analyzed using InterProScan (https://www.ebi.ac.uk/interpro/). If a protein had the same domains as the original *C*. *elegans* protein, then it was considered to be the reciprocal protein. The full list of proteins in *Strongyloides* genomes can be found in S1 Table and their sequences can be found in S1 Fig.

### Identification of the *daf-12* homolog in *S*. *ratti* and other species of *Strongyloides*

The *daf-12* homolog in *S*. *ratti* (SRAE_0000032100) was identified through protein BLAST (BLASTp) and translated nucleotide BLAST (tBLASTn) against the whole *C*. *elegans daf-12* protein and DNA-binding domain (DBD) specifically. All genes with a e-value of 0.0001 were carried forward and reverse searched for in the *C*. *elegans* genome by tBLASTn. Only those which produced *daf-12* as a likely hit were advanced forward and their domains were then determined using InterProScan. Only genes which contained the same domains as the *C*. *elegans daf-12* were carried forward. This revealed a single gene (SRAE_0000032100), which is considered to be the 1 to 1 ortholog of *daf-12* within *S*. *ratti*. The *daf-12* homologs of *S*. *stercoralis*, *S*. *papillosus* and *S*. *venezuelensis* were identified following the same strategy.

### siRNA design and synthesis

Each exon of Sr-*daf-12* was examined both manually and with the siDESIGN center (Dharmacon). Only sequences with a level of predicted on-target activity (>85%) and with no required modifications were considered. All potential sequences were then searched within the *S*. *ratti* genome using both nucleotide BLAST (BLASTn) and translated nucleotide BLAST (BLASTx) under normal parameters. Sequences with multiple alignments (>3 alignments) greater than 12 amino acids in length were discarded. The remain sequences with the highest predicted on-target activity were then ordered from and synthesized by Eurofins Genomics using the siRNAmax system. This system involves the use of a 19 nucleotide duplex with a 2 UU overhang on the 5’ end of both the sense and antisense strands. The list of siRNAs ordered can be found in Table 2.

### “Early-stage” Soaking of *S*. *ratti* with siRNAs

For “Early-stage” soaking experiments, L1 larvae were isolated from fecal culture by Baermann funnel and cleaned repeatedly with water. Worms were then transferred into RNAi culture medium (DMEM (Gibco), Octopamine 20mM (Sigma Aldrich), RNase OUT (Invitrogen) 40U/100μl) in a sterile Eppendorf tube, to which 10mM siRNA was added. The tubes were then incubated at 19°C for upto 3 days, following which the tubes were removed from the Incubator and briefly centrifuged at 1000g for 3 minutes to collect the worms at the bottom of the tube. The worms were then transferred onto a dry V12 agar plate [60] without any bacteria and examined for any visual phenotypic changes. Half the larvae were transferred into TRIzol (ThermoFisher) for RNA extraction, whilst the other half remained on the plate for use in further experiments.

### “Late-stage” Soaking of S. ratti with siRNAs

For the majority of experiments, 2 day old larvae (containing a mix of L3, L4 and free-living adults) were extracted from fecal culture by Baermann funnel and cleaned repeatedly with water. Worms were then transferred onto dry NGM [61] or V12 agar [60] without any bacteria. A modified protocol from [45] was developed to generate RNAi in S. ratti. Worms were then picked into the RNAi culture medium (DMEM (Gibco), Octopamine 20mM (Sigma Aldrich), RNase OUT (Invitrogen) 40U/100μl) in a clean Eppendorf tube, to which 10mM siRNA was added. The tubes were incubated at 19°C for upto 4 days, following which the tubes were removed from the Incubator and briefly centrifuged at 1000g for 3 minutes to collect the worms at the bottom of the tube. The worms were then transferred onto a dry V12 agar plate [60] without any bacteria and examined for any visual phenotypic changes. Half the larvae were transferred into TRIzol (ThermoFisher) for RNA extraction, whilst the other half remained on the plate for use in further experiments.

### RNA extraction, cDNA Synthesis and qRT-PCR

Nematodes (minimum of 10, maximum of 20) were isolated from plates post-soaking and transferred into TRIzol and instantly frozen in liquid nitrogen. The TRIzol/nematode mix was then frozen and thawed three times (with vortexing between freezing) using liquid nitrogen to ensure the cuticle of the worm was disrupted. After the addition of chloroform and centrifugation, the aqueous layer was transferred into a sterile Eppendorf tube and the RNA extracted from this layer using the RNA Clean & Concentrator-25 Kit (Zymo Research). RNA concentration and purity was then assessed using NanoDrop and the RNA then frozen at -80°C until needed. cDNA synthesis was then carried out using qScript XLT (Quantabio) as per the manufacturer’s protocol. As this kit has the reverse transcriptase already mixed into the buffer, half the RNA sample was retained for use as a negative control against DNA contamination. Following cDNA synthesis, expression level of *daf-12* along with three reference genes *(tbb-1*, *gpd-2*, *rpl-37* [55]) was determined by qRT-PCR using the Light Cycler 480 SYBR Green I Master Mix (Roche) on a LightCycler 480 II (Roche) machine. Briefly, 5μl of 2x Buffer was mixed with 1ul of each primer, 1μl of cDNA (diluted 1:10 in 1x TE buffer) and 2μl H_2_O and run according to the following protocol: Pre-incubation 95°C for 5 minutes, Amplification 95°C for 10 seconds, 57°C for 30 seconds, 72°C for 20 seconds, 40 cycles, Melting Curve 95°C for 5 seconds, 65°C for 1 minute, 97°C continuous, Cooling 40°C for 30 seconds. Amplification efficiency was evaluated for each primer pair by running dilution series in six technical replicates (S2 Table). All efficiencies were very close to the expected doubling per round of PCR which translates into a Ct difference of 3.32 for a 10 fold increase. Given this result and that we only compared the same amplicons in different treatments and made not quantitative comparisons of different genes (amplicons) we found it acceptable to use the 2^-ΔΔCt^ method [62]. The relative expressions and fold changes between treatments were calculated separately for each of the three reference genes. The fold changes shown in the figures are the mean values of the three measurements. The full list of primers used in this study can be found in S3 Table.

### Fecundity of soaked larvae

L3 worms were isolated as per the “Late-stage” soaking procedure above. After transferring the worms to a dry V12 agar plate without bacteria [60], females were then picked in groups of 10 onto V12 plates with a lawn of HB101. Every 24 hours for the next 96 hours post-transfer, the number of embryos laid was counted with embryos removed from the plate to avoid being counted twice.

### Developmental timing and infectivity of soaked larvae

L3/L4 worms were isolated and treated as per the “Late-stage” soaking procedure above. Following 48 hours of soaking, worms were transferred to a V12 agar plate [60] with a lawn of HB101 bacteria. Every 24 hours, all hatched larvae were examined and the total number of L3i was counted. Whether a larva had become a L3i was based upon morphology and whether the worm appeared to have ceased pumping. The L3i were then picked from the plate and stored in PBS at 19°C.

To test whether *daf-12(RNAi)* L3i were still able to infect rats, 100 *daf-12(RNAi)* L3i (also generated using the “Late-stage” soaking procedure) were injected subcutaneously into a rat. As a control, 100 scrambled siRNA treated L3i were injected subcutaneously into a separate rat. After 7 days incubation, the rat feces was collected daily overnight and incubated at 19°C as described above. The infective larvae were then collected from the water surrounding the feces and counted manually.

### *daf-12* RNAi effect upon direct development within *S*. *ratti*

L1 larvae were isolated from freshly collected rat feces and cleaned. Worms were then incubated using the “Early-stage” soaking procedure. Following treatment, the worm pellet was spun down in a centrifuge and pipetted onto a dry V12 agar plate without bacteria [60]. The total number of worms that had then undergone direct development to L3i and the number that had undergone indirect development to free-living adults were counted. Males were discarded from the analysis as it is not possible for them to undergo the direct development cycle.

### Measurement of fatty acid storage in *S*. *ratti*

L3/L4 larvae were isolated from rat feces and isolated using the Baermann funnel technique. Following cleaning with PBS, worms were soaked using the “Late-stage” soaking procedure. Following 48 hours soaking, the infective larvae produced were isolated into PBS and concentrated into a small volume by centrifugation. Once at a volume of 20μL or less, the worm pellet was frozen in liquid nitrogen and then sonicated using a Banderon Sonorex 100RH for 45 minutes. Following sonication, free glycerol and triglyceride levels were determined using the Serum Triglyceride Determination Kit (Sigma) according to the manufacturers protocol. To determine total protein concentration as a standard, protein concentration was calculated using DotBlot [63] against a BSA standard.

### Differential expression of Aerobic and Anaerobic Metabolism Genes by qRT-PCR

L3/L4 larvae were isolated from rat feces and isolated using the Baermann funnel technique and then treated using the “Late-stage” soaking procedure. Following 48 hours soaking, the infective larvae produced were isolated into PBS and concentrated into a small volume by centrifugation. The worm pellet was transferred into TRIzol and frozen in liquid nitrogen. RNA extraction, cDNA synthesis and qRT-PCR were performed as explained above. To measure aerobic metabolism, *acs-3* and *acbp-3* expression levels were measured. To measure anaerobic metabolism, *ech-8* and *acox-3* expression levels were measured. As a reference, expression of *tbb-1*, *rpl-37* and *gpd-2* [55] was measured. To determine the difference in expression between daf-12(RNAi) and control larvae, fold change for each reference gene was calculated as according [62]. The mean fold change of these three reference genes was then calculated. To account for DNA contamination, raw RNA was included in the qRT-PCR.

### Heat Treatment of soaked larvae

L1 larvae were isolated from rat feces using the Baermann funnel technique and then treated using the “Early-stage” soaking procedure. Following 3 days soaking, the worm pellet was collected by briefly centrifuging and then transferred to a V12 plate [60] with a lawn of HB101. Free-living adults were then picked in groups of 20 onto new plates. Plates were then incubated at either 12, 15, 19, 23, 28 or 37°C for 4 hours. After 4 hours, the plates were examined and the number of alive larvae counted. For each temperature, 3 technical replicates and 3 biological replicates were carried out.

### Data analysis

For all experiments, statistical analysis (student’s t-test or Mann-Whitney U) was carried out and figures were generated using Excel and Adobe Illustrator. Which test was used for each experiment is indicated in the corresponding figure legend. Statistical probabilities were considered significant once below 0.01.

## Supporting information

S1 Table Table of genes involved in RNAi in Strongyloididae based known *C. elegans* RNAi protein machinery. (PDF)

S2 Table Amplification efficiency calculations used for qRT-PCR. (DOCX)

S3 Table List of primer sequences used for qRT-PCR. (DOCX)

S1 Fig Protein sequences for known *C. elegans* RNAi proteins present within *Strongyloides ratti, Strongyloides papillosus, Strongyloides stercoralis* and *Strongyloides venezuelensis*. (PDF)

S2 Fig Protein sequences for daf-12 within *S. ratti, S. papillosus, S. stercoralis* and *S. venezuelensis*. (PDF)

S1 File Data File for all graphical information, means and statistical analysis used in this manuscript. (XLSX)
