# Correction: RNAi-mediated knockdown of *daf-12* in the model parasitic nematode *Strongyloides ratti*

**DOI:** 10.1371/journal.ppat.1008936

**Published:** 2020-09-11

**Authors:** Alex Dulovic, Adrian Streit

In the Materials and Methods section, the indicated concentration of the siRNAs in the working dilution was incorrect. It should read 10 μM (not mM).

A step-by-step protocol has been made available at the following link: https://www.sommerlab.org/fileadmin/uploads/RNAi_soaking_Alex.pdf
