# Supplementary material for: RNAi-mediated knockdown of daf-12 in the model parasitic nematode Strongyloides ratti
Source: PLoS Pathog. 2019 Mar 29;15(3):e1007705. doi: 10.1371/journal.ppat.1007705 (PMC6457571; doi:10.1371/journal.ppat.1007705)
Supplement: S1 Table — (PDF) [file ppat.1007705.s001.pdf]

Gene (C. elegans)	***S. ratti***	***S. papillosus***	***S. stercoralis***	***S. venezuelensis***
drh-3	SRAE_1000075900	SPAL_0000744900	SSTP_0001190100	SVE_1020200
drsh-1	SRAE_1000152900	SPAL_0001578300	SSTP_0000926600	SVE_0983200
xpo-1	SRAE_1000173900	SPAL_0001361000	SSTP_0001274200	SVE_0950800
xpo-2	SRAE_2000331500	SPAL_0000216400	SSTP_0000618100	SVE_1733500
dcr-1	SRAE_1000263400	SPAL_0001443300	SSTP_0000596200	SVE_0155200
drh-1	SRAE_1000075900	SPAL_0000744900	SSTP_0001190100	SVE_1020200
pash-1	SRAE_2000341100	SPAL_0000226300	SSTP_0000132700	SVE_1743700
rde-4	SRAE_1000315300	SPAL_0000492100	SSTP_0000623600	SVE_1949600
smg-2	SRAE_2000115100	SPAL_0001636400	SSTP_0000176800	SVE_0430100
smg-6	SRAE_2000102200	SPAL_0000476000	SSTP_0000727200	SVE_0454900
ego-1	SRAE_1000097100<br>SRAE_2000026400	SPAL_0001017300<br>SPAL_0001386500	SSTP_0000010600	SVE_0276300
rrf-3	SRAE_1000029300	SPAL_0000637300	SSTP_0000447900	SVE_0019900
rrf-1	SRAE_1000097100<br>SRAE_2000026400	SPAL_0001017300<br>SPAL_0001386500	SSTP_0000821100	SVE_0507500
rsd-3	SRAE_X000072500	SPAL_0001557000	SSTP_0000262800	SVE_0055100
tsn-1	SRAE_X000148500	SPAL_0001731600	SSTP_0000862900	SVE_0099900
ain-1	SRAE_2000380100<br>SRAE_X000088200	SPAL_0000585900<br>SPAL_0001296900	SSTP_0000418700<br>SSTP_0000495400	SVE_0052100<br>SVE_1648100
vig-1	SRAE_X000171800	SPAL_0000359300	SSTP_0000432100	SVE_0663200
ain-2	SRAE_2000380100<br>SRAE_X000088200	SPAL_0000585900<br>SPAL_0001296900	SSTP_0000418700<br>SSTP_0000495400	SVE_0052100<br>SVE_1648100
eri-1	SRAE_2000336900	SPAL_0000221600	SSTP_0000128100	SVE_1739000
xrn-2	SRAE_2000328200<br>SRAE_2000328700	SPAL_0001232100	SSTP_0000047400<br>SSTP_0000048000	SVE_1730200<br>SVE_1730800
xrn-1	SRAE_2000328200<br>SRAE_2000328700	SPAL_0000213400	SSTP_0000047400<br>SSTP_0000048000	SVE_1730200<br>SVE_1730800
eri-5		SPAL_0000160500		
eri-7	SRAE_1000168200	SPAL_0001494200	SSTP_0000434500	SVE_0739600
ekl-1	SRAE_1000250800	SPAL_0000859000	SSTP_0000307900	SVE_1049900
gfl-1	SRAE_1000024600<br>SRAE_2000130500	SPAL_0000188000<br>SPAL_0000813300	SSTP_0000293400<br>SSTP_0000808200	SVE_1504600
mes-2	SRAE_X000042600	SPAL_0000128500<br>SPAL_0000469700	SSTP_0000236300<br>SSTP_0001130100	SVE_1976300
ekl-4		SPAL_0000213200	SSTP_0000048200	SVE_1730000
mes-6	SRAE_X000134400	SPAL_0000145200	SSTP_0001231600	SVE_0902800
rha-1	SRAE_2000046600	SPAL_0000809400	SSTP_0000804700	SVE_1961700
ekl-6	SRAE_2000277100	SPAL_0001409900	SSTP_0000768300	SVE_1725500
zfp-1	SRAE_2000197500	SPAL_0001039400	SSTP_0000000500	SVE_0549100
mut-2			SSTP_0000186700	
