# Supplementary material for: RNAi-mediated knockdown of daf-12 in the model parasitic nematode Strongyloides ratti
Source: PLoS Pathog. 2019 Mar 29;15(3):e1007705. doi: 10.1371/journal.ppat.1007705 (PMC6457571; doi:10.1371/journal.ppat.1007705)
Supplement: S2 Table — (DOCX) [file ppat.1007705.s002.docx]

| *daf-12* | | | | | | | | | |
| --- | --- | --- | --- | --- | --- | --- | --- | --- | --- |
| **cDNA Dilution** | **Ct 1** | **Ct 2** | **Ct 3** | **Ct 4** | **Ct 5** | **Ct 6** | **Mean Ct** | **Slope** | **Efficiency (%)** |
| Undiluted | 22.10 | 22.31 | 21.89 | 22.00 | 22.08 | 22.04 | 22.07 | -3.41 | 96.45 |
| 0.1 | 25.35 | 25.24 | 25.29 | 25.61 | 25.39 | 25.49 | 25.40 |  |  |
| 0.01 | 28.52 | 28.72 | 28.70 | 28.67 | 27.90 | 28.50 | 28.50 |  |  |
| 0.001 | 32.44 | 32.28 | 32.32 | 32.32 | 32.49 | 32.46 | 32.39 |  |  |
| *tbb-1* | | | | | | | | | |
| **cDNA Dilution** | **Ct 1** | **Ct 2** | **Ct 3** | **Ct 4** | **Ct 5** | **Ct 6** | **Mean Ct** | **Slope** | **Efficiency (%)** |
| Undiluted | 19.02 | 19.12 | 19.11 | 19.09 | 19.33 | 19.24 | 19.15 | -3.30 | 100.92 |
| 0.1 | 22.41 | 22.47 | 22.67 | 22.49 | 22.50 | 22.51 | 22.51 |  |  |
| 0.01 | 25.58 | 25.61 | 25.62 | 25.59 | 25.79 | 25.36 | 25.59 |  |  |
| 0.001 | 29.10 | 29.20 | 29.02 | 29.11 | 29.14 | 29.19 | 29.13 |  |  |
| *gpd-2* | | | | | | | | | |
| **cDNA Dilution** | **Ct 1** | **Ct 2** | **Ct 3** | **Ct 4** | **Ct 5** | **Ct 6** | **Mean Ct** | **Slope** | **Efficiency (%)** |
| Undiluted | 21.01 | 20.99 | 21.04 | 21.02 | 20.88 | 21.08 | 21.00 | -3.24 | 103.54 |
| 0.1 | 24.02 | 24.13 | 24.09 | 24.18 | 24.20 | 24.12 | 24.12 |  |  |
| 0.01 | 27.43 | 27.37 | 27.40 | 27.41 | 27.35 | 27.50 | 27.41 |  |  |
| 0.001 | 30.64 | 30.71 | 30.71 | 30.77 | 30.72 | 30.69 | 30.71 |  |  |
| *rpl-37* | | | | | | | | | |
| **cDNA Dilution** | **Ct 1** | **Ct 2** | **Ct 3** | **Ct 4** | **Ct 5** | **Ct 6** | **Mean Ct** | **Slope** | **Efficiency (%)** |
| Undiluted | 18.46 | 18.55 | 18.50 | 18.47 | 18.45 | 18.52 | 18.49 | -3.26 | 102.65 |
| 0.1 | 21.87 | 21.92 | 21.88 | 22.01 | 21.97 | 22.03 | 21.95 |  |  |
| 0.01 | 25.03 | 25.04 | 25.06 | 25.00 | 25.03 | 25.12 | 25.05 |  |  |
| 0.001 | 28.28 | 28.30 | 28.45 | 28.29 | 28.33 | 28.34 | 28.33 |  |  |
| *daf-7* | | | | | | | | | |
| **cDNA Dilution** | **Ct 1** | **Ct 2** | **Ct 3** | **Ct 4** | **Ct 5** | **Ct 6** | **Mean Ct** | **Slope** | **Efficiency (%)** |
| Undiluted | 25.42 | 25.44 | 25.31 | 25.49 | 25.44 | 25.59 | 25.45 | -3.21 | 104.89% |
| 0.1 | 28.74 | 28.81 | 28.62 | 28.77 | 28.51 | 28.72 | 28.70 |  |  |
| 0.01 | 31.94 | 32.01 | 31.88 | 32.05 | 31.98 | 31.97 | 31.97 |  |  |
| 0.001 | 35.12 | 34.99 | 35.02 | 35.05 | 35.12 | 35.08 | 35.06 |  |  |
| *SRAE_X000150100* | | | | | | | | | |
| **cDNA Dilution** | **Ct 1** | **Ct 2** | **Ct 3** | **Ct 4** | **Ct 5** | **Ct 6** | **Mean Ct** | **Slope** | **Efficiency (%)** |
| Undiluted | 27.44 | 27.51 | 27.49 | 27.48 | 27.44 | 27.54 | 27.48 | -3.45 | 94.92% |
| 0.1 | 30.98 | 30.56 | 30.77 | 30.79 | 30.62 | 30.55 | 30.71 |  |  |
| 0.01 | 34.02 | 34.11 | 34.16 | 34.19 | 34.22 | 34.28 | 34.16 |  |  |
| 0.001 | 37.69 | 37.79 | 37.99 | 37.85 | 37.81 | 37.77 | 37.82 |  |  |
| *SRAE_1000103800* | | | | | | | | | |
| **cDNA Dilution** | **Ct 1** | **Ct 2** | **Ct 3** | **Ct 4** | **Ct 5** | **Ct 6** | **Mean Ct** | **Slope** | **Efficiency (%)** |
| Undiluted | 30.66 | 30.64 | 30.59 | 30.42 | 30.66 | 30.69 | 30.61 | -3.26 | 102.65% |
| 0.1 | 33.94 | 33.61 | 33.60 | 33.71 | 33.78 | 33.59 | 33.71 |  |  |
| 0.01 | 37.12 | 37.21 | 37.09 | 37.16 | 37.08 | 37.12 | 37.13 |  |  |
| 0.001 | n/a | n/a | n/a | n/a | n/a | n/a | n/a |  |  |
| *SRAE_1000214100* | | | | | | | | | |
| **cDNA Dilution** | **Ct 1** | **Ct 2** | **Ct 3** | **Ct 4** | **Ct 5** | **Ct 6** | **Mean Ct** | **Slope** | **Efficiency (%)** |
| Undiluted | 23.11 | 23.08 | 23.12 | 23.20 | 23.09 | 23.13 | 23.12 | -3.32 | 100.09% |
| 0.126.45 | 26.42 | 26.44 | 26.50 | 26.71 | 26.53 | 26.45 | 26.51 |  |  |
| 0.01 | 29.78 | 29.66 | 29.80 | 29.79 | 29.77 | 29.88 | 29.78 |  |  |
| 0.001 | 33.01 | 33.10 | 3.07 | 33.02 | 32.97 | 33.41 | 33.10 |  |  |
| *SRAE_2000402700* | | | | | | | | | |
| **cDNA Dilution** | **Ct 1** | **Ct 2** | **Ct 3** | **Ct 4** | **Ct 5** | **Ct 6** | **Mean Ct** | **Slope** | **Efficiency (%)** |
| Undiluted | 31.65 | 31.57 | 31.60 | 31.78 | 31.55 | 31.61 | 31.63 | -3.41 | 96.45% |
| 0.1 | 34.98 | 35.01 | 35.02 | 35.11 | 35.07 | 35.02 | 35.04 |  |  |
| 0.01 | 38.34 | 38.41 | 38.44 | 38.30 | 38.49 | 38.66 | 38.44 |  |  |
| 0.001 | n/a | n/a | n/a | n/a | n/a | n/a | n/a |  |  |
| *acs-3* | | | | | | | | | |
| **cDNA Dilution** | **Ct 1** | **Ct 2** | **Ct 3** | **Ct 4** | **Ct 5** | **Ct 6** | **Mean Ct** | **Slope** | **Efficiency (%)** |
| Undiluted | 21.62 | 21.57 | 21.55 | 21.49 | 21.88 | 21.76 | 21.65 | -3.36 | 98.44% |
| 0.1 | 24.98 | 25.03 | 25.11 | 25.07 | 25.32 | 25.02 | 25.09 |  |  |
| 0.01 | 28.12 | 28.20 | 28.22 | 28.31 | 28.41 | 28.36 | 28.27 |  |  |
| 0.001 | 31.78 | 31.80 | 31.68 | 31.72 | 31.74 | 31.62 | 31.72 |  |  |
| *acbp-3* | | | | | | | | | |
| **cDNA Dilution** | **Ct 1** | **Ct 2** | **Ct 3** | **Ct 4** | **Ct 5** | **Ct 6** | **Mean Ct** | **Slope** | **Efficiency (%)** |
| Undiluted | 22.55 | 22.67 | 22.61 | 22.68 | 22.71 | 22.73 | 22.66 | -3.27 | 102.21% |
| 0.1 | 26.01 | 26.09 | 25.87 | 26.00 | 26.03 | 26.11 | 26.02 |  |  |
| 0.01 | 29.33 | 29.50 | 29.36 | 29.41 | 29.38 | 29.30 | 29.38 |  |  |
| 0.001 | 32.69 | 32.88 | 32.81 | 32.80 | 32.79 | 32.66 | 32.77 |  |  |
| *ech-8* | | | | | | | | | |
| **cDNA Dilution** | **Ct 1** | **Ct 2** | **Ct 3** | **Ct 4** | **Ct 5** | **Ct 6** | **Mean Ct** | **Slope** | **Efficiency (%)** |
| Undiluted | 27.64 | 27.60 | 27.54 | 27.50 | 27.60 | 27.58 | 27.58 | -3.37 | 98.03% |
| 0.1 | 30.96 | 30.77 | 30.69 | 30.79 | 30.81 | 30.88 | 30.82 |  |  |
| 0.01 | 34.01 | 34.07 | 34.10 | 34.22 | 34.07 | 34.08 | 34.09 |  |  |
| 0.001 | 37.50 | 37.32 | 37.35 | 37.41 | 37.29 | 37.33 | 37.37 |  |  |
| *acox-3* | | | | | | | | | |
| **cDNA Dilution** | **Ct 1** | **Ct 2** | **Ct 3** | **Ct 4** | **Ct 5** | **Ct 6** | **Mean Ct** | **Slope** | **Efficiency (%)** |
| Undiluted | 28.55 | 28.51 | 28.40 | 28.49 | 28.60 | 28.52 | 28.51 | -3.34 | 99.25% |
| 0.1 | 31.90 | 32.12 | 32.11 | 32.07 | 32.30 | 32.01 | 32.09 |  |  |
| 0.01 | 35.24 | 35.33 | 35.34 | 35.28 | 35.61 | 35.51 | 35.40 |  |  |
| 0.001 | 38.70 | 38.67 | 38.57 | 38.56 | 38.57 | 38.61 | 38.61 |  |  |

n/a signifies this was beyond the limit of the detector.
