# Supplementary material for: RNAi-mediated knockdown of daf-12 in the model parasitic nematode Strongyloides ratti
Source: PLoS Pathog. 2019 Mar 29;15(3):e1007705. doi: 10.1371/journal.ppat.1007705 (PMC6457571; doi:10.1371/journal.ppat.1007705)
Supplement: S3 Table — (DOCX) [file ppat.1007705.s003.docx]

| Gene | | Sequence (5-3’) |
| --- | --- | --- |
| daf-12 | sense | CAGTTGAGAAACTGCATCGAA |
|  | antisense | GCAACCAAAGCTTTTGGAATTG |
| daf-7 | sense | TCTCTCAACATCCATGGAACTT |
|  | antisense | ACCAAGGTCATCAGTTGCAT |
| tbb-1 | sense | CCAATGCGGTAACCAAATC |
|  | antisense | TTCCACCATTTGCTTCATTG |
| gpd-2 | sense | GGATCTAATTTAATTGTACAACAAGATGGA |
|  | antisense | AGCACCCCATTGAATCTCAG |
| rpl-37 | sense | GGAGCTTCCCTTCGTAAACA |
|  | antisense | GTTCTGGTGCATTTTGAGCA |
| SRAE_X000150100 | sense | AGTTGTTCAAACCGATAGCATGA |
|  | antisense | TCAACATTAGGGGTTTGCGA |
| SRAE_1000103800 | sense | GCTGAGATTAAGAGACTTGAGCA |
|  | antisense | GTGTTAACTCATTCTTTTGGGCA |
| SRAE_1000214100 | sense | GGTAAACGTAAAGCTAACCAACA |
|  | antisense | TCTAAACCAGCAGCTTCTCCT |
| SRAE_2000402700 | sense | AGAGGCTGTATCAAGTTGGGT |
|  | antisense | TCCTATGCCTGCTAGAATTGC |
| acs-3 | sense | TCAGCAGAGGTTCTTGAAACT |
|  | antisense | TTACATGCAGCAGGACTTCC |
| acbp-3 | sense | AGCAAGTGGTCCATTAACAACA |
|  | antisense | GCCATTCACCAACATTAATTCCT |
| ech-8 | sense | AGGAGTCAGTGTTACAGTTGGA |
|  | antisense | TCCTGGACATGTCTTAACCAAT |
| acox-3 | sense | TGATACTCAAACTTTGGCTACCC |
|  | antisense | GGATTGGAGGTTTCGACTTCA |
