# Supplementary material for: RNAi-mediated knockdown of daf-12 in the model parasitic nematode Strongyloides ratti
Source: PLoS Pathog. 2019 Mar 29;15(3):e1007705. doi: 10.1371/journal.ppat.1007705 (PMC6457571; doi:10.1371/journal.ppat.1007705)
Supplement: S1 Fig — (PDF) [file ppat.1007705.s004.pdf]

C. elegans drh-3

> SRAE_1000075900

```
MFDPYHPVWRLVWKNNFLKKISNEKVYITMGLKKKNLLDSYNEFSKFHGENFKTFEETFY
AWAEMYFPEYFTEAIDTLKKDTANSDYFLSGISREMMKNVNLLFKKVKNMDGEFLILELL
MNNLQISDVLEYYISLNQDEYKYIVDVIKKLYDKFSNITRLSNGGYIKCLIFCERYIKKN
PVTVWEDFLKMLFLLEDKLGNILDILDPIKASIFRREKEDGDDKIKELIYSFDINMCNPV
KLEKFRNQYDYADYFYKNGQPQFLEIRNTYILRNYQVELIKYAIRGQNSIIMAPTGSGKT
LCAVEIIRSHILDNSSNGKGYRCLFIAPTGPLVLQQSQVLKAYLGDLYTVTILNKNSSEE
DVLSQFLAHDVIVCTPQLFLNNLLNNDKSKQLYFSDLSLIIFDECHHCNASHPYKQIMNL
FHGAIGDYQKPQIVGLTASIGTGKTITYEGCYNYYLEMAVNLHAKRICSVYKNRDSLIKV
MKLPVDEIIEIDVDQSLLWKNFNDTIKHLVNNLLKVDPTNNLNEHLNDYPISDVTKPGYF
SKISKLATFKAFVKDPEVFQWIDNCVKAFIHINLVKEVSQLLPISYVNIYCQDYLENIDS
YMKPSVSRDVIYKHLTNMKDTITNVIGQSKNSFPQYTPIFEKLLVICREQIKSLPDSRII
IFVPTRFIAVTLCHSIESINKVKNLNMQPGYIIANGNTRMLKQSPAEQNKTLENFRKGNI
NIIIATTIAEEGLDIDECNLVIKYNIAGNEKTLIQRRGRARALNAKSILLTCNNNYTSQE
VANLQKEKIMFEISETINCMSHSAFTKEVERKRAEMKSIADEKYKNEIERRKLLYGKVYD
VCCKYCKKSLIKSSAIKQLDENYIVLDEKIFKKTIIDIEKVPNPGLNFSRLANSHCKNCY
KNDISSAKIKIGEIIYIEDHFYFRIILKNISFLNVIDGDTYEERSRWDGVKNTLFLIPNV
TIGELIDYNSNFEKRDNKTYVALMECKEELKFQNNHLRIWKKAEEKEIKNLERFME
```

> SPAL_0000744900

```
MFDPYHKVWRLIWKNYFLEKISKEIGYITECLKRRNLLDSYNDFARLQSKNFSSFEEAFY
TWAERYFPEYFIEAIDLLKKEIPNSDYFLSGVSNEMMGNVNLLFKKVKEIDGEPLIIELL
MSNLHIGDVLDYYTNLNQEEYSYVVDVVKKLYEKFSNVRRLSNGGFIKCLIFCERFINRN
PNLIWEDFLKMLFLLEGKLGRIVDILDPVKASIIRREKEDRDAKIRELIYTVDVNINCPI
ELKSFRQQYDYVEHFNKNGHPKFNDIRNSYTLRDYQVELVTYAIRGENVIIMAPTGSGKT
LCAVEIMRLHMLENSSEGKGYRCLFIAPTGPLVLQQCQVLKNYLCDLYTVTTLSKNASED
EDVLKKILAHDVIVCTPQLFINQLMSNKESQKLYFSDFTLIVFDECHHCNSSHPYKQIMN
LFYRAEGDYEKPQIVGLTASIGTGKTVTHEGCYNYYLEMAVNLAAKRICGVYKNRETLNK
VMKLPVDEIIEFDVYQNDLIKYLDETTQLIVNELLRIDGASNLHEHLKSHPIGGVEKAGF
FSQVLKLSTFKAFVKNPEVLQWIDNAVKAFTHIYLVREASQILPILYVNLYCVDYLENVN
SVMRHSISRDILVGYLTDMKNKINNEIARNRSSFKTPPIYERLLKICNDQIKKLPNSRII
IFVPTRFVATTLCDSIVRICKAKGSDMRPGYIIANGNTKMTRQSPQDQKFTLDSFRSGDI
NIIIATTIAEEGLDIEECNLVVKYNVGGNEKSLIQRRGRARAQNAKSILLTCNNTYTSAE
ITNIQRERIMAEIAEKINSMSISKFTKEIEFKKMEMKRIADAKYENETERRRELYGKVYK
VCCRNCKEMITLSNTIRRFNTHHVVLDLKIFDKISIDTGRMANPGPTFSVVADGVCKNCC
HENEDGSIEKVKLGEIIYIDDHFYFKIILRNISFINNIDGDAYEERNRWDAVGNTLFLVP
NASFDEVDYYNLNFEKGTNKYYVELMECKEDLRIQNNNITIWKKMEEKELKELEKLIA
```

> SSTP_0001190100

```
MFNPYHKVWRLIWKNNFLEKISNEKSYIAKCLKEKNLLESYNEFAKLHGENFNSFDELFY
TWAERYFPEYFDEAINYLKKDIPNSDYFLYGTSKEMMVYVNLLFKKVKDMDGEPLILELL
MSNLQITDVLEYYKSLNQDEYEYVISVLKKLYDKFSNVTRLSTGGYIKCLIFCERFIKKN
PTTIWEDFLKMLFLLEHKIGNIVDILDPVRASIIRQEKEDIDNKIKELIFSVDINISNPI
ELKKFRIQSDYADYFNKNGQPKYLEVKNTYILRNYQVELIMYAIRGQNCILMAPTGSGKT
LCAVEIMRSHMFDKSIKGEGYRCLFLAPTGPLVLQQFQVLKNYLGDLYTVTTLSKSSSDD
EDVLSKILAHDVIVCTPQLFLNNLLYNDQSKQLYFSDLSLIIFDECHHCNSSHQYKQIMN
LFHRAEGNFSKPQIVGLTASIGTGKTVTYEGCYDYYLEMAINLDAKRICAVYKNRDTLIK
VMKLPVDEIIEVDVNLNILWENLNNTIQIIIDNLLEIDPTSSLNEHLKDFPISDVTKPGY
FSKISKLATFKAFIKNPELSQWIDNAVKAFIHIHLVKEVSQLLPISYVDVYCQKYLEDID
SVMKASVSRDIICKHLTNMRNAINDTITNNDNSISKCTPIFEKLLIVCREQIKKLSNSRI
IIFVPTRFIAVALYHSIELANKAKNLDMRSGYIIANGNSKIIKQSIADQKVTLEQFRKGT
INIIIATTIAEEGLDISECNLVVKYNVGGNEKTLIQRRGRARAQNAKSILLTCNNTYTMA
EITNIQKEKIMTEIAQTINCMSHSAFTKELEKKKIDMKKKADEKYENEIERKKQLYGKVY
NVCCRQCKNILTKSTTIRKFNTQHIVLDEHIFEKIFIDTQRMANPGLYFSVVADGNCKVC
NSNDVELTNKVKLGEIINIDDHFYFKIILRNISFISNVNGDTYEERSKWDAVGNTLFLVP
NVSIGELTDYNSNLQKSSKGKYSEFMECKEKLRIQNTNLAIWKKIEEKETKQLEKWID
```

> SVE_1020200

MFDPYHKVWRLVWKNYFLEKISKEKGYITECLIRKNLLDSYNDFARLQSKNFGSFEEVFY
TWAERYFPEYFIEAIDLLKEEIPNSDYFLSGSSNEMMQNVNFLFKKVKEIDGEPLVIEIL
MSNLQIRDVLDYYTNLNQEEYSYVVDVVKKLYEKFSNVRRLSNGGFIKCLIFCERFINRN
PNLIWEDFLKMLFLLEDKLGKIVDILDPVKASIIRREKEDTDAKIRELIYTVDVNINCPI
ELKSFRQQYDYVEHFNKNGHPKLNDIRNSYTLRDYQVELITFAVRGQNVIIMAPTGSGKT
LCAVEIMRLHMLENSSKGKGYRSLFIAPTGPLVLQQFQVLKNYLSDLYTVTTLSKNASED
EDVLMKILAHDVIVCTPQLFINQLMSSKESQKLYFSDFTLIVFDECHHCNSSHPYKQIMN
LFHRAEGDYEKPQIVGLTASIGTGKKNTHEGCFNFYLEMAINLAAKRICGVYKNRETLNK
VMKLPVDEIIEFDVYQNELIKYLNETTQFIVNELLKIDGASDLREHLKNHPIGGVEKAGF
FPQILKLSTFKAFVKNSEVLQWIDNAVKAFTHIYLVREASQILPILYVTLYCVDYLENVN
SVMRHSTSRDILVGYLMDMKNRISDEIGRNRSSFKPPPIYEKLLKICNDQIKKLPNSRII
IFVPTRFVATTLCDSIVRISKVKGSNMRPGYIIANGNTRMTKQSPAEQKLTLDDFRSGNV
NIIIATTIAEEGLDIEECNLVIKYNVGGNEKSLIQRRGRARAQNAKSILLTCNNTFTSAE
ITNIQKERIMAEISEKINSMSISKFTKEIELKKIEMKKIADAKYENEIERRQKLYGKVYK
VCCRNCKEIITLSHTIRRFNTHHVVLDLEIFDKISIDTGRMANTVSYFSVVADGICKKCW
YVNEDGSVEKTKIGEIIHIDDHFYFKIILRNISFINNIDGDMYEERTKWDAVGNTLFLVP
NASFDEIADYNLNFEKVTNKYYVKLMECKEDLRIQNNNITIWKKMEEKELKQLEKLMV

C. elegans drsh-1

> SRAE_1000152900

MSGKLLFLVVCAIILIIFTLFNISLNESTFSIKQLVPSSCNIENPSFEKLSDFLYTNVSI
FCLIHSSPKYKNKRAIHQKATWLKRCNNYIFVSSDEDKSLPAIKGGKEDGYQFSNERIRY
GLTYVVYENFGNKYDWFLKGDDDNYIIMENLRTFLLLRDSSIDQYYGFKLKLNHEYMSGAG
FILSKSALEKMVTIAFKNNTICSDKPNIPEDVEFGNCLKNINILPMDSRDSDNKHMFVPS
SFDEFSSMIKNSHFDGFVKMSPYEIKKGYTSLSKYPISFHYVTGDMFYGLEYLFYHASVI
GKESEVYKYDKYSYKNDFSISFLRLKEFIKKFYKFDANILDNNLYI

> SPAL_0001578300

MYSKKREYSSSSLGSIILSNNLKERQAKKPKFTEAEDISSDSDVSSNERSNSELSNTKTK
PKLISPKETDFNFSTESESESEVEDDDVPLSIFKEYTPLPITEKDILRSLELKGIDYKPD
QPPLMAQDVSTFKCIDDGKFYIKDRKNPHKLLQSDNLKSLVESFNEKIIYKLTNERALQP
ETKPFQSDNNTCDCESDSDDDHELVRLNQREKKSKKNIKKVSSKREQIATYEVLRKRSYS
AGMNKEIVFNEAGQKNNGAFCYCSKRHSSSGMRHGYYPNEKPIPKCNFASNEVDKLHHYI
LKIKTQLHDSLHNQTIISYNNNQFCFEGFSLFFHQEVPKNVTQCFEPVILEDGTIVEYEL
VSAQNDIMYTVGELEKFYQYFFMGIMELYDLHWYPKSCSFPDTENISCKIVHVLPRFICD
NHILPMAYVVKWMTDTFKPICTPETAVAVQTNVHALTKLRRNNIGRIAVNFNLRPCAIRV
DDISTVDIDDKKSNRYPKIYHRTTKIPEYSHIHAIEYQETRERANKLQYKFDRRGILSEG
EKYELKQLNEILKDYKNLSGRKKCREVSMSSYDYFCTNICSDMIEHAVVLISVVSLVRFN
NSLDTLEKEKLKYTFKDRRLLKLSLTHVSKFHHMGTNSDHIKNVLKNLGYKRQKSRNEYI
NSREKRRERDIAMQMNRHPNNERLEYLGDTVLEVIVTHHLFLTLPDIQEGGMATFRSAMV
QNRNLASLASKLMLDHYMLYAHGPDLCNETDFRHAMANTFEAVIGAMHLDGGTRECKNII
GSCMFDDCPVSLKLWNSPPLYNLQAKYKDGDRHLCEKYPYLNSLQNFEKEINIYFKNIRI
LAQVFCRTSLCKNALTEGDNERLEFLGDAVLQLVITEYLYQNFPDRDEGYMSQLRTCLVS
NKTQACVCDDLGLQRYIMPSKPNEDVCLIQMKMKDKADLVEALIGALFIDRGLDMCRLFI
CEMFMSRLHYFKDDMTWKDPKSYLQQFCLSKHLKPDGSSTGLLPKYKVLKKDIYNKTHFH
LVAVYFNDTRIGRGLGSNIVDAAQAASSAALEYLERKDVQAKVFRFDPNRDYSQV

> SSTP_0000926600

MNKEIVFNEAGQKNNGAFCYCSKRHNSSGMRHGYYPNEKPIPKCNFISNEFNKLHHYILK
IKTQVYGSVHNRTIINYNNTQFFFEGFSMLFHQKIPKNVTQCFEPVILEDGTVIEYELVS
TKNDITYTVGELEKFYHYFFVGIMEMYDLHWFPKSCAFPNTENISCKIVHIFPRFINNGT
ILPMANIIKWMTDTFKPVCTPEIAVAAQTNVHALTKLRRNNIGKIVVNFNLRPCAIRVDD
ISTIDTDDKKSNKYPKIYHRTTKIPEYSHVHAIEYQETKERANKLQNKLDRRGIISEGEK
YELKQLNEVLKDYKNLSGRKKCREVSMSSNNYFCTNICSDMIEHAVVLISVVSLVRFNNS
LDTLEKEKLKYTFKDRRLLKLSLTHVSKFHHMGTNSDHIKNVLRNLGYKRQKNKNDFINS

REKRRERDIALQINRHPNNERLEYLGDTVLEVIVTHHLFLTLPDIQEGGMATFRSAMVQN
RNLASLASKLMLDHYMLYAHGPDLCNETDFRHAMANTFEAVIGAMHLDGGTRECKNIIGS
CIFDDCEISLKLWNSPPLYNLQAKYKDGDRHLCEKYPYLNSLQKFEQEINIFFKNIRILA
QVFCRTSLCKNALTEGDNERLEFLGDAVLQLVITEYLYQNFPDRDEGYMSQLRTCLVSNK
TQACVCDDLGLQRYIMPSKPNEDVCSIQMKMKDKADLVEALIGALFIDRGIDICRLFIRE
MFMSRLHYFKDDMTWKDPKSYLQQYCLSKHLKPDGSSTGLLPKYKVLKKDIYNKTHYHLV
AVYFNDIRVGRGLGSNIVDAAQAASSAALEYLERKDVQAKVFKFDPNRDYSQT

> SVE_0983200

MYSKKREYSSSSLGSIILSNDLKERQIKKPRFTEAEDISSDSDVSSNERSNSELSNTKTK
LKLISPKEIDFNFSTESESESESEVEDDDVPLSIFKEYTPLPITEKDILRSLELKGIDYKPD
QPPLMTQDVSTFKCIDDGKFYIKDRKNPHKLLQSDNLKSLVESFNEKIIYKLTNERALQP
ETKPFQSDNNTCDCESDSDDDHELVRLNQREKKSKKNLKKVSSKREQIATYEVLRKRSYS
AGMNKEIIFNEAGQKNNGAFCYCSKRHSSSGMRHGYYPNEKPIPKCNFVSNEIDKLHHYI
LKIKTQLHDSLHNQTIISYNNNQFCFEGFSLFFHQEVPKNVTQCFEPVILEDGTIVEYEL
VSAQNDIMYTVGELEKFYQYFFMGIMELYDLHWYPKSCSFPDTENISCKIVHVLPRFISD
NYILPMAYVIKWMTDTFKPICTPEAAVAVQTNVHALTKLRRNNIGRIAVNFNLRPCAIRV
DDISTVDIDDKKSNRYPKIYHRTTKIPEYSHIHAIEYQETRERANKLQYKFDRRGILSEG
EKYELKQLNEILKDYKNLSGRKKCREVSMSSYDYFCTNICSDMIEHAVVLISVVSLVRFN
NSLDTLEKEKLKYTFKDRRLLKLSLTHVSKFHHMGTNSDHIKNVLKNLGYKRQKSRNEYI
NSREKRRERDIAMQMNRHPNNERLEYLGDTVLEVIVTHHLFLTLPDIQEGGMATFRSAMV
QNRNLASLASKLMLDHYMLYAHGPDLCNETDFRHAMANTFEAVIGAMHLDGGTRECKNII
GSCMFDDCPVSLKLWNSPPLYNLQAKYKDGDRHLCEKYPYLNSLQNFEKEINIYFKNIRI
LAQVFCRTSLCKNALTEGDNERLEFLGDAVLQLVITEYLYQNFPNRDEGYMSQLRTCLVS
NKTQACVCDDLGLQRYIMPSKPNEDVCLIQMKMKDKADLVEALIGSLFIDRGLDMCRLFI
CEMFMSRLHYFKDDMTWKDPKSYLQQFCLSKHLKPDGSSTGLLPKYKVLKKDIYNKTHYH
LVAVYFNDTRIGRGLGSNIVDAAQAASSAALEYLERKDVQAKVFRFDPNRDYSQV

C. elegans xpo-1

> SRAE_1000173900

MSINKETLLKAEAVLRSGDKFNVEALDVVVKAMSTGTGETSKLAMKIMTDLKADPDSWNK
VDVVLSKSEIPEAKFFALQILEDLITTRWKLLPREQCDGIKSFIVGLVLTVCNEVNTLDK
NHPAKAYLKKLNWVLVNIVKQEWPTYWPTFISEIVDSSRSKLFEFGHDITAKKQAHMKNQ
FCNEFEAVFKLCYEILSDSKSIPLIASTLETLNRFLQWIPVGYVFETEIVVFLVDKFLPH
PIFRSTAISCLAEIAGIKVSGTPEYEERIKYMMVNVIKIMSIQISLDIDFNSCYMNGKDE
DQKFISNFTKFLTTFLRQHSKILVIFKENENENTMPIKEAHMLALQYLLKISTIDDTEIF
KVCLDFWAELVTDLFRESPFSKSDSQIRLYAFDSRSSPNRTYYSEVISNLRSLMISKMAK
PEEVIVVVNENNEAVRESVKDTDAISLYKTMRETLVYLTHLDPNDTERQMTEKLQKQVDG
SEWSWNNLSRLCWAIGSISGAMHEDDERRFLVTVIRDLLALCEQKRGKDNKAVIASNIMY
VVGQYPRFLRAFWRFLKTVINKLFEFMHESHDGVQDMACDTFIKIVMKTKRHFIFVQNGE
EGPFIEEIIDNIGNIICDLSKAQVHVFYEALGIIVGSANDLALQKSWLHDMFKLPNDMWQ
DIINNVEKDPESFLEFKVLESIINVLKTYVSSCKSIGHPFVNVLNERFMDMMHMYTLSSK
YLDQGLKEIGESVCHQKVFKLLRSVKREILLLLSAWISHSKDKSIVLNNYMDLIIKNILN
DYFNCKHVVREPKVLSLMSIVAIHLEKQMNAYAATTFDCLFMSTLDMIKDDMITFPEHRV
NFYKLLQALFATCEQSIFEFPADRMETIIQAIVWGAQHQLRDVAETAIDLIACILRSAEF
LEPHQKVQFFQTHYMTILEHILGIVTDRNQVQFIGLSKLTVALCEAFSIPEYKYKGSLDP
ENAATNNVEYIYNYVYNLFKVHFTMLTDKQLEINIKGFFSYNLSPDKMREHIRDFLIQIK
TACGEDTEDLFLEERKREVEEAQARKNAIPGILNPNDVEDDMTDS

> SPAL_0001361000

MSISKETLLEAEAVLKNGEKFNVDALDVVVKAMSTGTGEISKLAMKIMTDLKSDPDSWNK
VDVVLSKSEVPEAKFFALQILEDLITTRWKLLPREQCDGIKNFIVGLVLTVCNEVNTLDR
NHPAKAYLKKLNWVLVNIVKQEWPTYWPTFISEIVDSSRSSESLCANNLYILILLSEELF
EFGHDITAKKQSHMKNQFCNEFEAVFKLCYEILSDSKSIPLIASTLETLNRFLQWIPIGY
VFETEIVVFLVDKFLPHPNFRCTAMSCLAEIAGIKVNGTPEYEEKIKYMMVNVIKIMSVQ
ISLDIDFNSCYMNGKDEDQKFLSNFTKFLTTFLRQHSKILVIFKENENENTRPIKEAHML
ALQYLLKISTIDDTEIFKVCLDFWAELVTDLFRESPFSKSDSQIRLYAFDSRNSPNRTYY
SEVISNLRSLMISKMAKPEEVVVVVNENNEAVRESVKDTDAISLYKTMRETLVYLTHLDP

NDTERQMAEKLQKQVDGSEWSWNNLSRLCWAIGSISGAMHEDDERRFLVTVIRDLLALCE
QKRGKDNKAVIASNIIAFWRFLKTVINKLFEFMHESHDGVQDMACDTFIKIVMKTKRHFV
IVQNGEEGPFIEEIINNIGNIICDLSKPQVHVFYEALGIIVGSVSDPELQRSLLIDMFRL
PNDMWQDIITNVEREPESFLEPKVLDSIINVLKTYVSSCKSIGHPFVNILNERFLDMMHM
YTLSSNYLDQGLKEIGESVCNQKIFKLLRSVKREILLLLSAWISHSKDKSIVLNNYMDLI
IENILKDYLNCKHILREPKVLSLMSIVAIHLEKDMNAYATVTFNCLFMSTLDMIKDDMVT
FPEHRVNFYKLLQAMFATCEQSIFGFSVDKMETIIQAIVWGAQHQLRDVAETAIDLISCI
LRSAEFLEPHQKMQFFQAHYMTILEHILGIVTDRNQVQFIGLSKLTIALCEAFSIPEYKY
KGSLDPENSATNNVEYIYNYVYNLFKVHFTMLTDKQLEVNIKGFFSYNLSPDKMRDHIRD
FLIQIKTACGEDTEDLFLEERKREVEEAQARKNAIPGIQNPNEIEDEMNDF

> SSTP_0001274200

MSNNKETLLKAEAILKNGDKFNVEALDVVVKAMSTGTGDTSKLAMKIMTDLKADPDSWNK
VDVVLSKSEVPESKFFALQILEDLITTRWKLLPREQCDGIKSFIVGLVLTVCNEVNTLDK
NHPAKAYLKKLNWVLVNIVKQEWPTYWPTFISEIVDSSRSSESLCANNLYILILLSEELF
EFGHDITAKKQAHMKNQFCNEFEAVFKLCYEILSDSKSVPLIASTLETLNRFLQWIPVGY
VFETETVVFLVDKFLPHPIFRCTAMSCLAEIAGIKVSGTPEYEERIKYMMVNIIKIMSIQ
ISLDIDFNSCYMNGKDEDQKFISNFTKFLTTFLRQHSKILVIFKENENENTIPIKEAHML
ALQYLLKISTIDDTEIFKVCLDFWAELVTDLFRESPFSKSDSQIRLYAFDSRNSPNRTYY
SEVISNLRSLMISKMAKPEEVIVVVNENNEAVRESVKDTDAISLYKTMRETLVYLTHLDP
NDTERQMTEKLQKQVDGSEWSWNNLSRLCWAIGSISGAMHEDDERRFLVTVIRDLLALCE
QKRGKDNKAVIASNIMYVVGQYPRFLRAFWRFLKTVINKLFEFMHESHDGVQDMACDTFI
KIVMKTKRHFVIVQNGEEGPFIEEIIDNIGNIICDLSKAQVHVFYEALGIIVGSVSDTTL
QRSWLIDMFKLPNDMWQDIINNVERDPESFLEPKLLESIINVLKTYVSSCKSIGHPFVNV
LNERFMDMMHMYTLSSKYLDQGLKEIGESVCHQKIFKLLRSVKREILLLLSAWISHSKDK
SIVLNNYMDLIIKNILNDYYNCKYILREPKVLSLMSIVAIHLEKEMNAFAATTFDCLFMS
TLDMIKDDMVTFPEHRVNFYKLLQAMFATCEQSIFEFPVDRMETIIQAIVWGAQHQLRDV
AETSIDLIACILKSAEVLEPYRKVQFFQAHYMTILEHILGIVTDRNQVQFIGLSKLTVAL
CEAFSIPEYKYKEGLDPENAATNNVEYIYNYVYNLFKIHFTMLTDKQLEINIKGFFSYNL
SPEKMREHIRDFLIQIKTACGEDTEDLFLEERRREVEEAQARKNAIPGIRNPNDTEDEMADS

> SVE_0950800

MSISKETLLEAEAVLKNGEKFNVDALDVVVKAMSTGTGEVSKLAMKIMTDLKSDPDSWNK
VDVVLSKSEVPEAKFFALQILEDLITTRWKLLPREQCDGIKNFIVGLVLTVCNEVNTLDR
NHPAKAYLKKLNWVLVNIVKQEWPTYWPTFISEIVDSSRSSESLCANNLYILILLSEELF
EFGHDITAKKQSHMKNQFCNEFEAVFKLCYEILSDSKSIPLIASTLETLNRFLQWIPVGY
VFETEIVVFLVDKFLPHPNFRCTAMSCLAEIAGIKVNGTPEYEEKIKYMMVNVIKIMSVQ
ISLDIDFNSCYMNGKDEDQKFLSNFTKFLTTFLRQHSKILVIFKENENENTRPIKEAHML
ALQYLLKISTIDDTEIFKVCLDFWAELVTDLFRESPFSKSDSQIRLYAFDSRNSPNRTYY
SEVISNLRSLMISKMAKPEEVVVVVNENNEAVRESVKDTDAISLYKTMRETLVYLTHLDP
NDTERQMAEKLQKQVDGSEWSWNNLSRLCWAIGSISGAMHEDDERRFLVTVIRDLLALCE
QKRGKDNKAVIASNIMYVVGQYPRFLRAFWRFLKTVINKLFEFMHESHDGVQDMACDTFI
KIVMKTKRHFVIVQNGEEGPFIEEIINNIGNIICDLSKPQVHVFYEALGIIVGSVSDPEL
QRSLLVDMFRLPNDMWQDIITNVEREPESFLEPKVLDSIINVLKTYVSSCKSIGHPFVNI
LNERFLDMMHMYTLSSNYLDQGLKEIGESVCNQKIFKLLRSVKREILLLLSAWISHSKDK
SIVLNNYMDLIIENILKDYLNCKHILREPKVLSLMSIVAIHLEKDMNAYATVTFNCLFMS
TLDMIKDDMVTFPEHRVNFYKLLQAMFATCEQSIFSFSVDKMETIIQAIVWGAQHQLRDV
AETAIDLISCILRSAEFLEPHQKMQFFQAHYMTILEHILGIVTDRNQVQFIGLSKLTIAL
CEAFSIPEYKYKGSLDPENSATNNVEYIYNYVYNLFKVHFTMLTDKQLEVNIKGFFSYNL
SPDKMRDHIRDFLIQIKTACGEDTEDLFLEERKREVEEAQTRKNAIPGIQNPNEIEDEMNDF

C. elegans xpo-2

> SRAE_2000331500

MVLNTLQGTLSSDKDVRLNAEMQMSQFTENAGFCNTLLKICDTRNGMNANFPESVITCAA
VAVKNFIKYQWTGNGTAPLDQLEREKIRQNVLEVLFGNSSDKKVRDQLLSSLTLIAKTDF
PEQWQNLATVLSEGLRGGISDSAKVSVISMDELFKRYRYEMKSEELWKEILYVLTICAEP
ITKFFVDTVKLVVESSEQKNHTPEQCREMFDCIFYCAEIFHSLNSQDLCEYFEDNLKVWM
EAFLTLMKINMDFYPFVVSDEEDPYNKLRVALCEIFTLFSQRYEEEFLPYMQPCVELMWE

KFFANSNVNNCDSLVNASMNFLGAICIRPQYKEMFTAEGVLVLLLRDIVIKNMVMSQDDI
ETFEDDPAEYLKKDLEGGDAHTKRRGAADLLKILSTDYPAQVSPIIQDLLKEFVGAFSAN
RKANWLQKEITYSMVTSMLVRGETARMGVTKLTDFVNLDDFYIQYVRPEIVESLPDELPI
ITCAALKFAFNFRHKLQPLALKELVSAPVVGRLLSSSNQIIHWYTGNMIDKLLNTKSQDN
KLLFTTADVDTPALISSIAQILELKSTSITPYVIKALMRVLNFMDDSTAAGADNIINALA
KLSFTALKQSANPVYIHYIFECMCVVIKKAYSRVAGGIDQAVLPVIEHIFTENMEDYVPY
ALQISALLISQCSAAIARGENVNSDAYHGFIQFVLTPQIWQHGTNVPAGVIALEAYIKAF
PNQMFSEENVKKLFDIYNKLIGSKANDQHGFTLGNILLPYLNKYPTLTFQSLFIPIYSRL
SRSKTFKLQKNMMIFVCRFVLIVGASEFVNCVNVIQNGLALMTMNRIFALELASIAQMTT
SFERKILILGMAKIFQNEPQIISESFVPLLNGVVKLIGASIKGISNVNSQVEMDEELLFD
EDNFNNKFCRLSVAKPEEDIFKEVKQYHRIFSDAIIKLQSSGVNFSPDCNESLELLKKYVQTTL

> SPAL_0000216400

MDNNVEMVLNALQGTLSSDKEIRQSAEMQMSQFTENAGFCCTLLKICDTRSGMNANIPEA
VVTCAAVAVKNFIRYQWTGSSGTAPLDQLEREKIRQSVLEVLFGNSGDKKVRDQLLSSLT
LIAKTDFPEQWQNLATVLSEALRGGISESAKVSVISMDELFKRYRYEMKSEELWREIRYV
LTVCAEPITKFFVEVVKMVVECSEKRNYTPEQCKEMFDCIFYCSEIFHSLNSQDLCEYFE
DNLKTWMEAFLALMKINMDFYPFSPSNEEDPYNKLRVSLCEIFTLFSQRYEEEFLPYMQP
CVELIWEKFFANGNADNCDSLINASMNFLGAICIRPQYQSMFTAEGVLALLLRDIVIKNM
VMSQDDIETFEDDPAEYLKKDLEGGDAHTKRRGAADLLKILSTDYPAQVTPIVQDLLKEF
VGAFSANRKSNWLQKEITFSMITSMLIRGETARMGATKVTDFVNLDDFYTQYVRPEIVES
LPDELPIITCSALKFAFTFRHKLQPLALKELVSGPVVGRLLASKNQIIHWYTGNMIDKIL
NTKTRDNKLLFTSSDVDTPALIGSIAQILELRSTSITPYVIKALMRVLSFMDDSTAAGAD
NIINALAKLSFTALKQSANPVYIHYIFECMCVVIKKAYSKVAGGIDKAVLPVIEHIFTEN
IEDYIPYALQISALLITQCSAAISRGENVNSDAYHGFMQFVLTPQIWQHSTNAPAGVIAL
EAYINAFPNQMFSEENVKKLFDIYNRLIGSKANDQHGFSLGNALLPYLNKYPSITVQSLF
IPIFSRLSRSKTFKLQKNMMIFVCRFILVVGADEFVRSVNAIQDGLATMTMNRVFAIELT
GIAQMTTSFERKMLILGMAKLFESQPHVISESFGALFNGVVKLIGASIKGISNVSSHVEV
DEELVFDEDNFNNKFCRLSVSRPEEDIFKEYKQYHRIFAEAVSKLQGSGISFPPECDESLVLLKKYVETVL

> SSTP_0000618100

MDNNVEIVLNALQGTLNSVKEVRQNAEMQMSQFTENAGFCNTLLKICDTRNGINVNVPGA
VITCAAVAVKNFIKYQWTGNGVAPLDQLEREKIRQGLLEVLFGNSGDKKVRDQLLSSLTL
IAKTDFPEQWQNLATVLSEALNGGMSESAKVSLISMDELFKRYRYEMKSDALWKEILYVL
GICADSITRFFINTVKLVVECSEKKNASFEQCKEMFECIYYCAEIFHSLNSQDLCEYFED
NLKIWMEAFLALMKINMDFYPYAPSNEEDPYNKLRVSLCEVFTLFSQRYEEEFLPYMQSC
VELIWEKFFANGSADNCDSLVNASMNFLGAICVRQQYKEMFTADGVLVLLLRDIVIKNMV
MSEADIETFEDDPAEYLKKDLEGGDAHTKRKGAADLLKILSTDYPAQVSPIIQDLLKEFI
GAFSANRKANWLQKEITFSMITSMLVRGETARMGVTKLTDFVNLDDFYSQYVRPEIVDSP
TDELPIITCAALKFAVTFRHKLQPLALKELVSAPVIGRLLASKNYIIHWYTGNMIDKLLN
TKSNDNKLLFTTSDVDTPALISSIAQILELKSTSITPYVIKALMRVLSFMDDSTAASADN
IINALAKLSFTALKQSSNPIYIHYIFECMCVVIKKAYSKVAGGIDKAVLPVIENIFAENI
EDYIPYALQISALLITQCSAAILRGENISSDAYHGFIQFVLTPQIWQHSTNVPAGVIAIE
AYIKAFPDQMFSEENVKKLLDIYSKLITSKANDQHGFSLGNALLPYINKYPCLNVSSLFI
PIFSRLSRSKTFRLQKNMMIFVCRFVLVVGVEEFVNSVNAIQNDLALMTMNRIFALELAN
IAQMTSSFERKILILGMAKIFQSHPQIIRESFVQLFYGVVKLIEASIKGVATSNIQLEVD
DELMFDEDNFNNKFCRLSVAKPEEDIFKEFKQYHRIFAESVLKLESSGLNFPPECNEMLGMLKKYTEVTL

> SVE_1733500

MDNNVEMVLNALQGTLSSDKEIRQSAEMQMSQFTENAGFCCTLLKICDTRSGMNANIPEA
VVTCAAVAVKNFIRYQWTGNSGTAPLDQLEREKIRQSVLEVLFGNSGDKKVRDQLLSSLT
LIAKTDFPEQWQNLATVLSEALRGGISESAKVSVISMDELFKRYRYEMKSEELWREIRYV
LTVCAEPITKFFVEVVKMVVDCSEKRNYTPEQCKEMFDCIFYCSEIFHSLNSQDLCEYFE
DNLKTWMEAFLALMKINMDFYPFSPSNEEDPYNKLRVSLCEIFTLFSQRYEEEFLPYMQP
CVELIWEKFFANGNADNCDSLINASMNFLGAICIRPQYQSMFTAEGVLALLLRDIVIKNM
VMSQDDVETFEDDPAEYLKKDLEGGDTHTKRRGAADLLKILSTDYPAQVTPIVQELLKEF
VGAFTANRKGNWLQKEITFSMITSMLIRGETARMGATKVTDFVNLDDFYSQYVRPEIVES
LPDELPIITCSALKFAFTFRHKLQPLALKELVSGPVVGRLLASKNQIIHWYTGNMIDKIL
NTKTRDNKLLFTNSDVDTPALIGSIAQILELKSTSITPYVIKALMRVLNFMDDSTAAGAD

NIINALAKLSFTALKQSANPVYIHYIFECMCVVIKKAYSRVAGGIDKAVLPVIEHIFTEN
IEDYIPYALQISALLITQCSTAIARGENVNSDAYHGFMQFVLTPQIWQHSTNVPAGVIAL
EAYINAFPNQMFSEENVKKLFDIYNRLIGSKANDQHGFSLGNALLPYLSKYPSITVQSFF
IPIFSRLSRSKTFKLQKNMMIFVCRFILVVGADEFVRSVNAIQDGLATMTMNRVFAIELT
GIAQMTTSFERKILILGMAKLFESQPQVISESFGPLFNGVVKLIGASIKGISNVSSHVEV
DEELVFDEDNFNNKFCRLSVSRPEEDIFKEFKQYHRIFAEAVSKLQSSGISFPPGCDESLVLLKKYVETVL

C. elegans dcr-1

> SRAE_1000263400

MTNRSFEVSDEVFTPRPYQLEILEKAKKRNVIVQLPTGSGKTYIGILMIKEVQYTVRKSI
SEGGKRIFFVVNNVCLVEQQARHIKNECELVVGELHGESSITVHDTDKIKTFLEQHQVIV
LTAQILLDLICFSRISMEDISLLIFDECHHSMGKAHPYSNILSRYDKVPNEKKPVILGLT
ASLFNQRLKKNQIENLLLKLEYKMHAEVVTADDFSQVLKYSAIPNIKLIECSDFNAEKFD
IAQKTLSFVDKLNELVREMEKLLKDSRDFDPSQFIKTDINSTIANSIYEKFIDTSSKDIR
KKISEFVYITNNLGPFTVYKICDFWTGDLKNYKNDQTISVKANYVVEAAFDVFDSIKKEY
TKKFSTMVTFEEIEPYLSNKLKKVIEILETFNKKCIEDPSVFQSMSCIIFVERRSAAYAI
FEVLKELKKLDKARFGFIKADYIVGYNNNKSVEETTISSKIQDKKLQSFRQGSLNVLVST
DVLEEGFDVRQCNLVIRYKFPKNFRSYVQGRGRVRHKQGLYILLSDSKSLEDDKAELKNF
EECETLLIQRFRTPNNFVDNKKSINCPDFYVDSLYDSYVVESTGATAVLSNAITIINRYC
QKLPCDNFTVLSPSPDIIENEDGTYECSLLLPANCPYRNIIRSKKPLPSAKLSKMAAAVE
ACKILHQIKELNDNLLPNGREKLKLIETKFDDYEDEVDIKEVIVGSAQRKQLYDKKSSIY
LYDILPLENEESYLYIFDIDLKQPIHSYMNLKNRKIDNPQDYECAFGFLSKKVIPDMPSF
PIFLRYGCAEVTVKKHPKKYLISLENWDKAIKFHEYIFSDILRVTDVATFSAAQAIVKIV
TLPLKKIKLESDNEFDYEIDFNYMDYILETLEGMFKPPLEEDRKKYVFNESLYENAIVYP
WYRSEENRNYYIIGEIMHDVKPSSDFPDNNFKTFEEYFLEKYKITIYNKDQPLLDVDYTS
NRLNLLVPKYPSKSSRPLKNSRSSQRQILVPELVSIHPIKSTHWSIIGALPSIFYRYNCL
LMANELRETIVKNALGKTNVYRGPFEPLSYKTCLEKDLKDEKNLNEIIEEETDQVEEDIT
DFEIGVWDPQYIDTFDILKPVKEIPDQLNNDVQCLKASYPDDEKMESDIDDIDEEKMGTF
KFLSVDERPEIKPVTESSFTLQKLESLSGWDDPCIDNQEIKSVLHISDVDASIDKKALVR
DMNDVNELEETEYLDEKFSTYPIKKKHLVDIEKKFVDLREYEQSESPPPITKDDFNIHMK
LDEFEDLGQNWKTIVRDNYELLIEQPVMPYKLDDEGIDTDDLYSKGVNPAILLCALTSRG
ANDGIDLERLETIGDSFLKFATTDYLYHKHVNAHEGCLSLLRSKEVSNMKLYFLGKKRNI
HNIIINDKFEPHTNWIPPGYVVSSEFKPNYVQQNADKEDKDAEAILNCDFESDDKLSELC
SEMEEGKNLKKSNSEFKFKKVRQFNELSNIVYNPYLQQQICDKTIADVVEALIGCHLIHI
GFDGTLKFMQWMGISVFSHHYEAVEIDPMIKILDFPQNPNKSMQCLYGFYKKNLFDIFEQ
KIGYIFKNKAYLIQAFSHASYYINRVTSCYQRLEFLGDAVLDFVITRYLFSHEASFNPGT
LTDLRSALVNNTMLASVAVDHDFHKYLMEFSPKLHEVVRKFVTFIKSHENELINLDSDLF
MVNEDEEDCDGTEDVEVPKALGDIFESFVGAVYLDSGRNLNVVWALIYKMMKPHLERYTK
EPPISPIRELTESYPDKVQFSKVERDPNTQRVKVYVEVSGTKITGGGRNFKIAKCNAAKR
ALKYIKQLEKERAKNQNIL

> SPAL_0001443300

MTGRSFEVSDEQFTPRPYQLEILEKAKKRNVIVQLPTGSGKTYIGILMIKEVQYTVRETI
SKGGKRIFFIVNNVALVEQQAKHIRNECELVVGELHGDSSVKVENLDKINDFLDKNQVIV
LTAQILVDLISHGRICLENVSLLIFDECHHSMGKAHPYSNILAKYDRVSNEKKPIILGLT
ASLFNQRLKKSQIETLLLKLEHKMHSEVVTADDFSQILKYSARPNVKLVVCDNFNVTDFE
IVEKTLNLVYNLNDLVRQMETVLKDTSDIESGNLTGNNISTEMANTIFEKFLDSGLKEVR
KKTSEFIYVTTSLGPFTVHRVIDFWTRELKELSGDKTYPEKISLLIKSAYDVFDSIKKEY
TSIFSNLTTFEEIMEYLPGKLLKVLEILEAFNNKCAENGGSPQSMSCIIFVERRSSAYAI
FEVLKELKKINKERFGFIKADYIVGYNNYRSEEVVTASSRAQDKKLQNFRQGSLNVLVST
DVLEEGIDIRQCNLVIRYQFPKNFRSYVQGRGRVRHKEGLYLLLSDKKSIEDDSNELQNF
EECEALLIKRFQTPNNCCDDMKNLSCPRLYIDDLYDSYVVESTGAKVSMSNAISIVNKYC
QKLPCDNFTVLSPTPEIIVNDDGTFDCKLLLPANCPYREVIQTKKSLPRAKYAQMAAAVE
ACKILHQIKELNDNLLSNGREKLKLIETKFDDYEDEVDIKEVIVGSAQRKQLYDKKSSIY
LYDILPSVDEESYLYIFDIDLKQPIHSYMNLKNRRIDNPQDFECAFGFLSKKIIPDMPSF
PIFLRCGFAEVTVKRHPKKYSISQDDWEKSIKFHEYIFSDILRVTDVATFSATQAIVKIV
TLPLKKIKVDGDEKYDYEIDFNYMNYILDSLEGMFKAPLEEDRKNYTFDESLYENAIVYP
WYRSEENRNYYIIGEIMHDVKPCSDFPDNNFKTFEEYFLEKYKITIYNKDQPLLDVDYTS
NRLNLLVPKYPTKSSRPLKGSRSSQRQILIPELVSIHPIKSTHWSIIGALPSIFYRYNCL

LMANELREIIVKSALGKSDVYGGPFEALSYRTCLEKDSKDEKNPNEIIEEEGEADQVEED
ITDFEIGVWDPKCVETLDILKPVQEPLTEFENDIQGLKASYPDDEKMESDIEDMDEEKLG
TFKFLPEDKRPIINPIAEPSFDLEKLKSLGGWDDPTIDDQEINSVLHISDVDASIDKKAL
VRDMNDVNELDEGEYLDNHFSSYPIKKKRVVDVEKKFVDLREYEQSESPPPIIDPDFNFH
MKIEGFDDLGHEWKDIVRDNYELLIDQPVMSYRLDDEGIEIDDLYSKGVNPAILLCALTS
RGANDGIDLERLETIGDSFLKFATTDYLYHKHVDAHEGCLSFLRSKEVSNMKLYVLGKKR
NIHNIIINEKFEPHTNWIPPGYVVSSEFKPNYVQQNADKEDKDAEAILNCDFESDDRLSE
LCNGMEKEKSSKKNSSEYKFKKVRQFNELSNIVYNPYLQQQICDKTIADVVEALIGCHLI
HIGFNGTLKFMQWMGISVFSHHFEAVESDPLMRILDSIEEPNKSMECLYGFYRKNSFDIF
EQKVGYVFKNKAYLIQAFSHASYYVNRVTGCYQRLEFLGDAVLDFVITRYLFSHEANFNP
GTLTDLRSALVNNTMLASVAVDHDFHKYLMEFSPKLHEVIKKFVTFIKSHENELINLDSD
LFMVNEDEEDCDGTEDVEVPKALGDIFESFVGAVYLDSGRNLNVVWALIYKMMKPHLERY
TKEPPISPIRELTESYPDKVQFSKMERDPNTQRVKVYVDVSGSKITGGGRNFKIAKCNAA
KRALKYIKQLEKERAKNQSAL

> SSTP_0000596200

MASRGFEVSDELFIPRPYQLEILEKAKKRNVIVQLPTGSGKTYIGILMIKEVQYTVRKSI
SEGGKRIFFIVNNVSLVEQQARHIRNECELVVGELHGESSVNVCDLEKIKAFLDKNQVIV
LTAQILVDLICFKRINVEDISLMIFDECHHSMGKAHPYSNILSKYDSVPNEKKPIILGLT
ASLFNQRLKKNQIEKLLLKLEYKMHSEVVTADDFSQILKYSAIPNVKLIECADFDIEVFD
IAKKTLNFTDKLSEYLRETEKSLKNSKDFDSNQFMKSDISPQIASTIFNKLVDSLTKGIR
KKINEFTYITNYLGPFTVYKLCDFWIKELKDLINEKSIPSKIYDVAEAAFDVFDSIKKEY
VRKFSTMTTFEEIQPYLSSKIKKIIEILEAFNKKCIENPDVFQNLSCIIFVERRLAAYAI
FEILKELRRMDKKRFGFIKADYIVGYNGNKPLEEKSASSNVQDKKLQSFRQGLLNVLVST
DVLEEGIDVRQCNLVIRHRFPRNFRSYVQGRGRVRHKEGLYILLSESKSLEDDKNELKNF
EECETLLVQRFKTPNNHCENFKNLNCSEMYIDCLYDTYTVESTGAKVSLSNSISIINKYC
QKLPCDNFTVLSPTPEIIVNDDKTFECSLLLPANCLYRSVIRSTKPLPNTKLAQKAAAVE
ACKILHQIKELNDYLLPNGREKLKIIETKFDDYEDELDIKEVIVGSAQRKQLYDKKSSIY
LYDILPLVNEESYLYILDIDLKQPIHNYMNLKNRKIDNPQDYECAFGFLSKKIIPDMPSF
PIFLRYGCAEVTVKRHPKKYLITQENWDKAIKFHEYIFSDILRVTDVATFSAAQAIVKIV
TLPLKKIKLENDNEFDYKIDFDYMNYILETLEGMFKPPLEEDRKKYVFNESLYENAIVYP
WYRSEENRNYYIIGEIMHDVKPSSDFPDNNFKTFEEYFLEKYKITIYNKDQPLLDVDYTS
NRLNLLVPKYPSKSSRPLKGSRSSQRQILVPELVSIHPIKSTHWSIIGALPSIFYRYNCL
LMANELRETIVKNALGKTDVYRGPFEPLVYKTCLEKDPKDENNLNKIIEEETNRAEEDIT
DFEIGVWDPKCVETVDVLKPVKEISDELDSSIKCLQASYSDDEKMESDIDDMDEEKLGTF
KFLSADERPIIRPVTESSFNLQKLKNLGGWDDPNVDASIDKKALVRDMNDVNELEETEYL
DENFSSYPIKKKHVVDVEKNFVDLREYEQSESPPPIINTDFNIHMKLEEFEDLGQNWESI
VRDNYELLIEKPLMSYKLDDEDINTDELYSKGVSPAILLCALTSRGANDGIDLERLETIG
DSFLKFATTDYLYHKHVDAHEGCLSFLRSKEVSNMKLYVLGKKRNIHNIIINDKFEPHTN
WIPPGYVVSSEFKPNYVQQNADKEDKDAEAILNCDFESDDKIFELCNGIEKDKNSKKNNS
EYKFKKVRQFNELSNIVYNPYLQQQICDKTIADVVEALIGCHLIHIGFDGTLKFMQWMGI
SVFSHHNEAIESDPMIRVLDSQEEPHRSMQCLHGFYTKNSFDIFERKVGYIFKNKAYLIQ
AFSHASYYINRVTGCYQRLEFLGDAVLDFVITRYLFSHEANFNPGTLTDLRSALVNNTML
ASVAVDHDFHKYLMEFSPKLNEVIKKFVTFIKSHENELINLDSDLFMVNEDEEDCDGTED
VEVPKALGDIFESFVGAVYLDSGRNLNVVWALIYKMMKSHLERYTKEPPISPIRELTESY
PDKVQFSKVERDPNTQRVKVYVEVSGTKITGGGRNFKIAKCNAAKRALKYLKQLEKERAKNQNTL

> SVE_0155200

MTGRSFEVSDEQFTPRPYQLEILEKAKKRNVIVQLPTGSGKTYIGILMIKEVQYTVRETI
SKGGKRIFFIVNNVALVEQQAKHIRNECELVVGELHGDSSVKVENLDKINDFLDKNQVIV
LTAQILVDLISHARICLENVSLLIFDECHHSMGKAHPYSNILAKYDSVPNEKKPIILGLT
ASLFNQRLKKSQIEDLLLKLEHKMHSEVVTADDFSQILKYSARPSVKLVVCDNFNVTDFK
IGEKTLNLVYKLNDLVREMETVLKDTSDIESSNLTGNNISTEMANTIFEKFLESGLKEVR
KKTTEFIYVTTSLGPFTIYRVIELWTRELKELSSDKTYPEKISLLIKSAYDVFDSIKKEY
ASIFSGLTTFESIVEYLPGKLLKVIEILEVFNKKCTENGGSPQSMSCIIFVERRSSAYAI
FEVFKELKKINKERFGFIKADYVVGYNNYKSEEVVTASSRAQDKKLQNFRQGSLNVLIST
DVLEEGIDIRQCNLVIRYQFPRNFRSYVQGRGRVRHKEGLYLLLSDKKSIEDDENELQNF
DECEALLIKRFQTPNNCCDDMKNLSCPRFYIDDLYDSYVVESTGAKVSLSNAISIVNKYC
QKLPCDNFTVLSPTPEIIENDDGTYSCKLLLPANCPYREVIQTKKSLPRAKYAQMAAAVE
ACKILHQIKELNDNLLSNGREKLKLIETKFDDYEDEVDIKEVIVGSAQRKQLYDKKSSIY
LYDILPSVDEESYLYIFDIDLKQPIHSYMNLKNRRIDNPQDFECAFGFLSKKIIPNMPSF

PIFLRCGFAEVTVKRHPRKYSISQENWEKSIKFHEYIFSDILRVTDVATFSAAQAIVKIV
TLPLKKIKIDGDEKYDYEIDFNYMNYILDSLEGMFKPPLEEDRKNYTFNESLYENAIVYP
WYRSEENRNYYIIGEIMHDVKPCSDFPDNNFKTFEEYFLEKYKITIYNKDQPLLDVDYTS
NRLNLLVPKYPTKSSKPLKGSRSSQRQILIPELVSIHPIKSTHWSIIGALPSIFYRYNCL
LMANELREIIVKNALGKSEVYGGPFEPLSYRTCLEKDSKDEKNPNEIIEEEGEADQLEED
ITDFEIGVWDPKCVETLDILKPIQEPLTEFENDIQGLKASYHDDEKMESDIEDMDEEKLG
TFKFLPEDKRPIINPIAELSFDLEKLKSLGGWDDPTIDDQEINSVLHISDVDAGIDKKAL
VRDMNDVNELDEGEYLDNHFSSYPIKKKRVVDVEKKFVDLREYEQSESPPPTIDPDFNFH
MKIEGFNDLGHEWKDIVRDNYELLIDQPVMSYRLDDEGIEIDDLYSKGVNPAILLCALTS
RGANDGIDLERLETIGDSFLKFATTDYLYHKHVDAHEGCLSFLRSKEVSNMKLYVLGKKR
NIHNIIINEKFEPHTNWIPPGYVVSSEFKPNYVQQNADKEDKDAEAILNCDFESDDRLSE
LCNGMEKEKSSKKNNSEYKFKKVRQFNELSNIVYNPYLQQQICDKTIADVVEALIGCHLI
HIGFNGTLKFMQWMGISVFSHHYEAVESDPLMRILDSIEEPNKSMECLYGFYRKNSFDIF
EQKVGYVFKNKAYLIQAFSHASYYVNRVTSCYQRLEFLGDAVLDFVITRYLFSHEANFNP
GTLTDLRSALVNNTMLASVAVDHDFHKYLMEFSPKLHEVIKKFVTFIKSHENELINLDSD
LFMVNEDEEDCDGTEDVEVPKALGDIFESFVGAVYLDSGRNLNVVWALIYKMMKPHLERY
TKEPPISPIRELTESYPDKVQFSKMERDPNTQRVKVYVDVSGSKITGGGRNFKIAKCNAA
KRALKYIKQLEKERAKNQSAL

C. elegans drh-1

> SRAE_1000075900

MFDPYHPVWRLVWKNNFLKKISNEKVYITMGLKKKNLLDSYNEFSKFHGENFKTFEETFY
AWAEMYFPEYFTEAIDTLKKDTANSDYFLSGISREMMKNVNLLFKKVKNMDGEFLILELL
MNNLQISDVLEYYISLNQDEYKYIVDVIKKLYDKFSNITRLSNGGYIKCLIFCERYIKKN
PVTVWEDFLKMLFLLEDKLGNILDILDPIKASIFRREKEDGDDKIKELIYSFDINMCNPV
KLEKFRNQYDYADYFYKNGQPQFLEIRNTYILRNYQVELIKYAIRGQNSIIMAPTGSGKT
LCAVEIIRSHILDNSSNGKGYRCLFIAPTGPLVLQQSQVLKAYLGDLYTVTILNKNSSEE
DVLSQFLAHDVIVCTPQLFLNNLLNNDKSKQLYFSDLSLIIFDECHHCNASHPYKQIMNL
FHGAIGDYQKPQIVGLTASIGTGKTITYEGCYNYYLEMAVNLHAKRICSVYKNRDSLIKV
MKLPVDEIIEIDVDQSLLWKNFNDTIKHLVNNLLKVDPTNNLNEHLNDYPISDVTKPGYF
SKISKLATFKAFVKDPEVFQWIDNCVKAFIHINLVKEVSQLLPISYVNIYCQDYLENIDS
YMKPSVSRDVIYKHLTNMKDTITNVIGQSKNSFPQYTPIFEKLLVICREQIKSLPDSRII
IFVPTRFIAVTLCHSIESINKVKNLNMQPGYIIANGNTRMLKQSPAEQNKTLENFRKGNI
NIIIATTIAEEGLDIDECNLVIKYNIAGNEKTLIQRRGRARALNAKSILLTCNNNYTSQE
VANLQKEKIMFEISETINCMSHSAFTKEVERKRAEMKSIADEKYKNEIERRKLLYGKVYD
VCCKYCKKSLIKSSAIKQLDENYIVLDEKIFKKTIIDIEKVPNPGLNFSRLANSHCKNCY
KNDISSAKIKIGEIIYIEDHFYFRIILKNISFLNVIDGDTYEERSRWDGVKNTLFLIPNV
TIGELIDYNSNFEKRDNKTYVALMECKEELKFQNNHLRIWKKAEEKEIKNLERFME

> SPAL_0000744900

MFDPYHKVWRLIWKNYFLEKISKEIGYITECLKRRNLLDSYNDFARLQSKNFSSFEEAFY
TWAERYFPEYFIEAIDLLKKEIPNSDYFLSGVSNEMMGNVNLLFKKVKEIDGEPLIIELL
MSNLHIGDVLDYYTNLNQEEYSYVVDVVKKLYEKFSNVRRLSNGGFIKCLIFCERFINRN
PNLIWEDFLKMLFLLEGKLGRIVDILDPVKASIIRREKEDRDAKIRELIYTVDVNINCPI
ELKSFRQQYDYVEHFNKNGHPKFNDIRNSYTLRDYQVELVTYAIRGENVIIMAPTGSGKT
LCAVEIMRLHMLENSSEGKGYRCLFIAPTGPLVLQQCQVLKNYLCDLYTVTTLSKNASED
EDVLKKILAHDVIVCTPQLFINQLMSNKESQKLYFSDFTLIVFDECHHCNSSHPYKQIMN
LFYRAEGDYEKPQIVGLTASIGTGKTVTHEGCYNYYLEMAVNLAAKRICGVYKNRETLNK
VMKLPVDEIIEFDVYQNDLIKYLDETTQLIVNELLRIDGASNLHEHLKSHPIGGVEKAGF
FSQVLKLSTFKAFVKNPEVLQWIDNAVKAFTHIYLVREASQILPILYVNLYCVDYLENVN
SVMRHSISRDILVGYLTDMKNKINNEIARNRSSFKTPPIYERLLKICNDQIKKLPNSRII
IFVPTRFVATTLCDSIVRICKAKGSDMRPGYIIANGNTKMTRQSPQDQKFTLDSFRSGDI
NIIIATTIAEEGLDIEECNLVVKYNVGGNEKSLIQRRGRARAQNAKSILLTCNNTYTSAE
ITNIQRERIMAEIAEKINSMSISKFTKEIEFKKMEMKRIADAKYENETERRRELYGKVYK
VCCRNCKEMITLSNTIRRFNTHHVVLDLKIFDKISIDTGRMANPGPTFSVVADGVCKNCC
HENEDGSIEKVKLGEIIYIDDHFYFKIILRNISFINNIDGDAYEERNRWDAVGNTLFLVP
NASFDEVDYYNLNFEKGTNKYYVELMECKEDLRIQNNNITIWKKMEEKELKELEKLIA

> SSTP_0001190100

MFNPYHKVWRLIWKNNFLEKISNEKSYIAKCLKEKNLLESYNEFAKLHGENFNSFDELFY
TWAERYFPEYFDEAINYLKKDIPNSDYFLYGTSKEMMVYVNLLFKKVKDMDGEPLILELL
MSNLQITDVLEYYKSLNQDEYEYVISVLKKLYDKFSNVTRLSTGGYIKCLIFCERFIKKN
PTTIWEDFLKMLFLLEHKIGNIVDILDPVRASIIRQEKEDIDNKIKELIFSVDINISNPI
ELKKFRIQSDYADYFNKNGQPKYLEVKNTYILRNYQVELIMYAIRGQNCILMAPTGSGKT
LCAVEIMRSHMFDKSIKGEGYRCLFLAPTGPLVLQQFQVLKNYLGDLYTVTTLSKSSSDD
EDVLSKILAHDVIVCTPQLFLNNLLYNDQSKQLYFSDLSLIIFDECHHCNSSHQYKQIMN
LFHRAEGNFSKPQIVGLTASIGTGKTVTYEGCYDYYLEMAINLDAKRICAVYKNRDTLIK
VMKLPVDEIIEVDVNLNILWENLNNTIQIIIDNLLEIDPTSSLNEHLKDFPISDVTKPGY
FSKISKLATFKAFIKNPELSQWIDNAVKAFIHIHLVKEVSQLLPISYVDVYCQKYLEDID
SVMKASVSRDIICKHLTNMRNAINDTITNNDNSISKCTPIFEKLLIVCREQIKKLSNSRI
IIFVPTRFIAVALYHSIELANKAKNLDMRSGYIIANGNSKIIKQSIADQKVTLEQFRKGT
INIIIATTIAEEGLDISECNLVVKYNVGGNEKTLIQRRGRARAQNAKSILLTCNNTYTMA
EITNIQKEKIMTEIAQTINCMSHSAFTKELEKKKIDMKKKADEKYENEIERKKQLYGKVY
NVCCRQCKNILTKSTTIRKFNTQHIVLDEHIFEKIFIDTQRMANPGLYFSVVADGNCKVC
NSNDVELTNKVKLGEIINIDDHFYFKIILRNISFISNVNGDTYEERSKWDAVGNTLFLVP
NVSIGELTDYNSNLQKSSKGKYSEFMECKEKLRIQNTNLAIWKKIEEKETKQLEKWID

> SVE_1020200

MFDPYHKVWRLVWKNYFLEKISKEKGYITECLIRKNLLDSYNDFARLQSKNFGSFEEVFY
TWAERYFPEYFIEAIDLLKEEIPNSDYFLSGSSNEMMQNVNFLFKKVKEIDGEPLVIEIL
MSNLQIRDVLDYYTNLNQEEYSYVVDVVKKLYEKFSNVRRLSNGGFIKCLIFCERFINRN
PNLIWEDFLKMLFLLEDKLGKIVDILDPVKASIIRREKEDTDAKIRELIYTVDVNINCPI
ELKSFRQQYDYVEHFNKNGHPKLNDIRNSYTLRDYQVELITFAVRGQNVIIMAPTGSGKT
LCAVEIMRLHMLENSSKGKGYRSLFIAPTGPLVLQQFQVLKNYLSDLYTVTTLSKNASED
EDVLMKILAHDVIVCTPQLFINQLMSSKESQKLYFSDFTLIVFDECHHCNSSHPYKQIMN
LFHRAEGDYEKPQIVGLTASIGTGKKNTHEGCFNFYLEMAINLAAKRICGVYKNRETLNK
VMKLPVDEIIEFDVYQNELIKYLNETTQFIVNELLKIDGASDLREHLKNHPIGGVEKAGF
FPQILKLSTFKAFVKNSEVLQWIDNAVKAFTHIYLVREASQILPILYVTLYCVDYLENVN
SVMRHSTSRDILVGYLMDMKNRISDEIGRNRSSFKPPPIYEKLLKICNDQIKKLPNSRII
IFVPTRFVATTLCDSIVRISKVKGSNMRPGYIIANGNTRMTKQSPAEQKLTLDDFRSGNV
NIIIATTIAEEGLDIEECNLVIKYNVGGNEKSLIQRRGRARAQNAKSILLTCNNTFTSAE
ITNIQKERIMAEISEKINSMSISKFTKEIELKKIEMKKIADAKYENEIERRQKLYGKVYK
VCCRNCKEIITLSHTIRRFNTHHVVLDLEIFDKISIDTGRMANTVSYFSVVADGICKKCW
YVNEDGSVEKTKIGEIIHIDDHFYFKIILRNISFINNIDGDMYEERTKWDAVGNTLFLVP
NASFDEIADYNLNFEKVTNKYYVKLMECKEDLRIQNNNITIWKKMEEKELKQLEKLMV

C. elegans pash-1

> SRAE_2000341100

MDMSQFDKDIPPPPPPPEICPFSNEPLYIKEQNKTSSVESQDSDDSLVDIAMDKKDLLKEK
NKNTEITIKHPNNETFFIPNNEVKPYAPPIKIPKNFITNCVSTRTKNLPEGWKAIAHDSG
HFVYLHLGTRVVTYSKPFELKDGSARHHDTPLASIPCLEQKKHLEKIKLKQESGKLTDIS
EYEVISPEELKNYSTSLFEYETKYITKIDKNMPKSERKRKFADYFNAEDDEIEEEEIPDH
FPCKPKSGFPSNGHLINIECPGTYGKKARTVQFNPVGKSSTNILHEYVQRSMKTKVLYSE
DQFDFDAYVFHCSCYLIINEIVKRNIINNERIIEKLGKIETRVYRDEEQIFIGKGKGKSK
REAKLAAGVNSVKLFLEDLTFDDNGTCKAIGGKSIEESDIIQFFKSIDLEHAKLADLCEK
SGQLTPNAILQIAIRNHPNAGTFKLQSDCQTTSHGRHIFSLSFGNLAVDYECKNKKEGKQ
VAAQKFIKLLHPECTTWGEIIEIYGTKSQSNKEAKLSAQKNVIKMASEFMEESKAKMKHS
YEPNLRVLNALKEHHKKFYCEFSQDCIEQHINDIKRPILYPDIAAKASRKKEMEDPTSLWKNAL

> SPAL_0000226300

MDMSQFDKDIPPPPPPPEICPFSNEPLYIGEDKKTPSIESQDSDDSLVDIAMNKKDLLKNN
NKDNDTNVKSPDNESFSTTNKEVKSYAPPIKIPKNFITNCVSTRTKNLPDGWKAIAHDSG
HFVYLHLGTRVVTYSKPFELKDGSARHHDTPLASIPCLEQKKHLEKVKQKQESGKLTDIS
EYETISSEELKNYSTSLFEYETKYITKFDKNMPKSERKRKYADYFVTEGDDIEEEEEIPD
HFPCKPKGGFPSNGHLINIECPGTYGKKARTVQFNPVGKSSTNILHEYIQRSMKTKVHYA

EEQFDFDAYVFHCSCYLIINEIVKRNIINNERIVEKLGKIETRINRDEEQLFVGKGKGKS
KREAKLAAGVNSVKLFLEDLTFDDNGICKAIGGKSVEESDIIQFFKSIDLEHAKLADLCE
KSGQLTPNAVLQIAIRNHPNAGTFKLQSDCQTTSHGRHIFSLSFGNLAVDYECKNKKEGK
QIAAQKFIKLLHPECTTWGEIIEIYGTKSQSTKEAKLSAQKNVIKMASEFMEESKAKMKH
SYEPNLRVLNALKEHHRKFYDQFSQDCIEQHINDIKRPILYPDIAAKASRKKEMEYPTSLWKNAL

> SSTP_0000132700

MDMSQFDKDIPPPPPPPEICPFSNEPLYVKEDRKTLSTESRDSDDSLVDIAMNKKDLLKEK
NENTEIITKNLNNETFYIPNNEVKSYAPPIKIPKNFITNCVSTRTKNLPEGWKAIAHDSG
HFVYLHLGTRVITYSKPFELKDGSARHHDTPLASIPCLEQKKHLEKMKQKQESGKLTDIS
EYEIISPEELKNYSTSLFEYETKYITKIDKNMPKSERKRKYADYFMTEDDDINEEEIPDH
FPCKPKSGFPSNGHLINIECPGTYGKKARTVQFNPVGKSSTNILHEYVQRSMKTKVLYSE
DQFDFDAYVFHCSCYLIINEIVKRNIINNERIIEKLGKIETRIYRDEEQIFIGKGKGKSK
REAKLAAGVNSVKLFLEDLTFDDNGTCKAIGGKSIEESDIIQFFKSIDLEHAKLADLCEK
SGQLTPNAILQIAIRNHPNAGTFKLQSDCQTTSHGRHIFSLSFGNLAVDYECKNKKEGKQ
VAAQKFIKLLHPECTTWGEIIEIYGTKSQSNKEAKLSAQKNVIKMASEYIEESKTKMKHS
YEPNIRVLNALKEHHKKFYNEFSQDCIEQHINDIKRPILYPDIAAKASRKKEMEDPTSLWKNAL

> SVE_1743700

MDMSQFDKDIPPPPPPPEICPFSNEPLYIGEDKKTPSMESQDSDDSLVDIAMNKKDLLKNN
NKDSDTNVNSPDNEPFSTTNKEVKSYAPPIKIPKNFITNCVSTRTKNLPDGWKAIAHDSG
HFVYLHLGTRVVTYSKPFELKDGSARHHDTPLASIPCLEQKKHLEKVKQKQESGKLTDIS
EYETISSEELKNYSTSLFEYETKYITKFDKNMPKSERKRKYADYFVTEGDDIEEEEEIPD
HFPCKPKGGFPSNGHLINIECPGTYGKKARTVQFNPVGKSSTNILHEYIQRSMKTKVHYA
EEQFDFDAYVFHCSCYLIINEIVKRNIINNERIVEKLGKIETRINRDEEQLFVGKGKGKS
KREAKLAAGVNSVKLFLEDLTFDDNGICKAIGGKSVEESDIIQFFKSIDLEHAKLADLCE
KSGQLTPNAVLQIAIRNHPNAGTFKLQSDCQTTSHGRHIFSLSFGNLAVDYECKNKKEGK
QIAAQKFIKLLHPECTTWGEIIEIYGTKSQSTKEAKLSAQKNVIKMASEFMEESKAKMKH
SYEPNLRVLNALKEHHKKFYDQFSQDCIEQHINDIKRPILYPDIAAKASRKKEMEYPTSLWKNAL

C. elegans rde-4

> SRAE_1000315300

MCSESTNLKEVLAKKIPEHNKKVKDFRAKHGSDAIQQVTIDMIYGGMRSMKGMVTETSVL
DPEEGIRFRGYSIPECQKLLPKAPGGEEPLPEAIWWLLCTGDIPTEKQVAVISKEWAARA
ELPEHVARMLDNFPSNLHPMSQLIAATSALNTESKFAQAYSNGVHKSTYWEYTYEDSMNL
LAKLPTIAAMIYRNLYRDGTSVGVIDSKKDWSANFASMLGYDDPLFTELLRLYLVIHSDH
EGGNVSAHTSHLVGSALSDPYLSFSAAMAGLAGPLHGLANQEVLIFLSKMKKDLGTSYTE
EDLRKWVWDHLKSGQVVPGYGHAVLRKTDPRYTCQREFALKHLPNDELFKLVSTLYKVTP
NVLLEQGKAKNPWPNVDAHSGVLLQHFGLKEMNYYTVLFGVSRALGCLSQLIWARGMGLP
LERPKSHSTEGLMKLVSKK

> SPAL_0000492100

MSAISSFARRSLLVSKNVSPLFSAQASMCSETTNLKEVLAKKIPEHNKKVKDFRAKHGGD
KVQDVTIDMIYGGMRSMKGMVTETSVLDPEEGIRFRGYSIPECQKLLPKAPGGEEPLPEA
IWWLLCTGDIPTDKQVAAISKEWAARAELPEHVVRMLDNFPSNLHPMSQLVAAAAALNSE
SKFAQAYANGVHKSTYWEYTYEDSMNLLAKLPTIAAMIYRNLYRDGTSVGVIDTKKDWSA
NFASMLGYEDPLFTELLRLYLVIHSDHEGGNVSAHTSHLVGSALSDPYLSFSAAMAGLAG
PLHGLANQEVLIFLSKMTKDLGTSYSEEDLRKWVWNHLKSGQVVPGYGHAVLRKTDPRYT
CQREFALKHLPNDDLFKLVSTLYKVTPNVLLEQGKAKNPWPNVDAHSGVLLQHFGLKEMN
YYTVLFGVSRALGCLSQLIWARGMGLPLERPKSHSTEGLMKLVSKK

> SSTP_0000623600

MSTLSSFARRSLLVSKNAGQIINAQASLCSESTNLKEVLAKKIPEHNKKVKEFRAKHGAD
FVQQISVDMIYGGMRTIKGMVTETSVLDPEEGIRFRGYSIPECQKLLPKAPGGEEPLPEA

IWWLLCTGDIPTDKQVATISKEWAARAELPEHVARMLDNFPSNLHPMSQLVAASAALNSE
SKFAQAYSNGVHKSTYWEYTYEDSMNLLAKLPTIAAMIYRNLYRDGTSVGVIDSKKDWSA
NFASMLGYDDPLFTELLRLYLVIHSDHEGGNVSAHTSHLVGSALSDPYLSYSAAMAGLAG
PLHGLANQEVLIFLSKMQKDLGTKYTEEDLRKWVWDHLKSGQVVPGYGHAVLRKTDPRYT
CQREFALKHLPNDELFKLVSTLYKVTPGVLLEQGKAKNPWPNVDAHSGVLLQHFGLKEMN
YYTVLFGVSRALGCLSQLIWARGMGLPLERPKSHSTEGLMKLVSKK

> SVE_1949600

MSAISSFARRSLLVSKNVSPLFSTQASMCSETTNLKEVLAKKIPEHNKKVKDFRSKHGND
KVQDVTIDMIYGGMRSMKGMVTETSVLDPEEGIRFRGYSIPECQKLLPKAPGGEEPLPEA
IWWLLCTGDIPTDKQVAAISKEWVSRAELPEHVVRMLDNFPSNLHPMSQLVAAAAALNSE
SKFAQAYANGVHKSTYWEYTYEDSMNLLAKLPTIAAMIYRNLYRDGTSVGVIDTKKDWSA
NFASMLGYEDPLFTELLRLYLVIHSDHEGGNVSAHTSHLVGSALSDPYLSFSAAMAGLAG
PLHGLANQEVLIFLSKMTKDLGTSYTEEDLRKWVWNHLKSGQVVPGYGHAVLRKTDPRYT
CQREFALKHLPNDDLFKLVSTLYKVTPNVLLEQGKAKNPWPNVDAHSGVLLQHFGLKEMN
YYTVLFGVSRALGCLSQLIWARGMGLPLERPKSHSTEGLMKLVIKK

C. elegans smg-2

> SRAE_2000115100

MAAASQDSEYEENLDLDDIDSVIDNVQKTDEGSDDDEGEYDGLLGPDFSKLPPHACRYCG
IHNVDQVLQCSTCEKWFCNGKGITTGAHILQHMIRAQHREINFHKDAELGDSVLECYQCG
IKNIFLLGFVPAKNDNVIVVLCRNCSSMATKSANWCADEWHCLIEERSLLPWIAKVPSVK
ERMKAFRITTAQIKALEELWRVNPSADISDLHKPGLDEAVEPVQRTYKDAYNYRRILAPL
IRLEADYDKAAKEALKPTVSNVKWDYAKNKKILATFQIQELLTSGIKLMIGDELKLKHNQ
TLLGTTWECHGHLIKVPDHHSEDFVLEFDESFQLPSAKRIIYTVEFIWNATSYTRMYNAL
DTLAQKPNCVSPYIYDKLMGHPTSEVLLNVKLPKSFNVPGLPDLNHSQVNAVRQALRKQL
TLIQGPPGTGKTVCSANLIYHLVHQRKGKILVCAPSNIAVDQLAEKLHQAKLKVVRLCAK
GREDVQSPVAFLSLHNQLKTLTQDTDLQTLLKMKEEGGGLSAADESRLKAIIKAKEIVLL
SRADVICCTCVCAADSRISFLKFKSVLIDECSQATEPEIMVSVVRARSKLILVGDHCQLG
PVVMCEKANAAGFSQTLFERLMLNGNVPIRLQVQYRMHPAMSAFPSNVFYEGSLQNGVTS
SDRILSNLTFQWPVPNKPIIFWHTDGTEELSPTGTSYLNRAEAINVELLATMFLNSGVKP
EQIGIITPYGGQRAYIVQHMHSCGTLHYKLYNEIEVANVDAFQGREKDLIIVSCVRSNEN
NGIGFLSDPRRLNVALTRSKYGLVIIGNAKVLSKNILWNHLLLNYREQGCFMDGHINNLR
PSQIPLPKERSLIEFFKKHTRFVSPALSMTEPVKLQTVDLTKTYPTGMQNPNSFIFQNTH
PSGAYQPFTFRQPVLGQANGFQNAIQPQYESTRQGVTKKNKNNSRSGSSYNQRGNDVLSQ
EMEFGTLHSQEMSQTSISFSRMSESQMSAPYDIEPNMSQIAKDLNGLMLSEDSKI

> SPAL_0001636400

MAAKEDGLCLSENDDYEHMAAASQDSEYEENLDLDDIDSVIDNTNNKTDEGSDDEGEYDG
LLGPDFSKLPPHACRYCGIHNVNQVLQCSTCEKWFCNGKGITTGAHILQHMIRAQHREIN
FHKEAELGDSVLECYQCGVKNIFLLGFVPAKNDNVIVVLCRNCSSMATKSANWCADEWHC
LIEERSLLPWIAKVPTVKERIKSFRITTAQIKALEELWRINPSADISDLHKPGLDEAVEP
VQRTYKDAYNYRRILAPLIRLEADYDKAAKEALKPTVSNVKWDYAKNKKILATFQIQELL
TSGIKLMIGDELKLKHNQTLLGTTWECHGHLIKVPDHHSEDFVLEFDESFQLPSAKRIIY
TVEFIWNATSYTRMYNALDTLAQKPNCVSSYIYEKLMGHPTSEALLNVKLPKSFNVPGLP
DLNHSQVNAVRQALRKPLTLIQGPPGTGKTVCSANLIYHLVHQRKGKILVCAPSNIAVDQ
LAEKLHQTKLKVIRLCAKGREDVQSPVAFLSLHNQLKTLTQDTDLQTLLRMKEEGGGLSA
TDESRLKIIIKAKEKVLLSKADVICCTCVCAADSRISFLKFKSVLIDECSQATEPEIMVS
VVKARSQLILVGDHCQLGPVVMCEKANAAGFSQTLFERLMLNGNVPIRLQVQYRMHPAMS
AFPSNVFYEGSLQNGVTSSDRILSNLTFQWPVPNKPIIFWHTDGTEELSPTGTSYLNRAE
AINVELLATMFLNSGVRPEQIGIITPYGGQRAYIVQHMHACGTLHYKLYNEIEVANVDAF
QGREKDLIIVSCVRSNENNGIGFLSDPRRLNVALTRSKYGLVIIGNAKVLSKNILWNHLL
LNYREQGCFMDGHINNLRPSQIPLPKERSLIEFFKKHTRFVSPALSMTEPVKLQTVDLTK
SYPTGMQNPNSFIFQNTHPSATYQPFVFRQPTLGQVNGFQNAIQSQCENTRQGVNKRTKN
NHSNSRSGSSYSQRGNDMLSQEMEYGTLQSQEMSQTSISFSRMSESQMSAPFDIEPNMSQ
IARDLNGLMLSEDSKI

> SSTP_0000176800

MAAASQDSEYEENLDLDDIDSVIDNAQKTDEGSDDEEEYDGLLGPDFSKLPPHACRYCGI
HNVDQVLQCSTCEKWFCNGKGITTGAHILQHMIRAQHREINFHKDAELGDSVLECYQCGI
KNIFLLGFVPAKNDNVIVVLCRNCSSMATKSANWCADEWHCLIEERSLLPWIAKVPSVKE
RMKAFRITTAQIKALEELWRINPSADISDLHKPGLDEAVEPVQRTYKDAYNYRRILAPLI
RLEADYDKAAKEALKPTVSNVKWDYAKNKKILATFQIQELLTSGIKLMIGDELKLKHNQT
LLGTTWECHGHLIKVPDHHSEDFVLEFDESFQLPSAKRIIYTVEFIWNATSYTRMYNALD
TLAQKPNCVSPYIYEKLMGHPTSEVLLNVKLPKSFNVPGLPDLNHSQVNAVRQALRKQLT
LIQGPPGTVHQRKGKILVCAPSNIAVDQLAEKLHQAKLKVVRLCAKGREDVQSPVAFLSL
HNQLKTLTQDTDLQTLLRMKEEGGGLSAADESRLKTIIKAKEIVLLSRADVICCTCVCAA
DSRISSLKFKSVLIDECSQATEPEIMVSVVRARSKLILVGDHCQLGPVVMCEKANAAGFS
QTLFERLMLNGNVPIRLQVQYRMHPAMSAFPSNVFYEGSLQNGVTSSDRILSNLTFQWPV
PNKPIIFWHTDGTEELSPTGTSYLNRAEAINVELLATMFLNSGVKPEQIGIITPYGGQRA
YIVQHMHSCGTLHYKLYNEIEVANVDAFQGREKDLIIVSCVRSNENNGIGFLSDPRRLNV
ALTRSKYGLVIIGNAKVLSKNILWNHLLLNYREQGCFMDGHINNLRPSQIPLPKERSLIE
FFKKHTRFVSPALSMTEPVKLQTVDLTKTYPTGMQNPNSFIFQNSHPSGTYQPFVFRQPV
LGQANGFQNAIQPQYESTRQGATKKNKNNHNNSRSGSSYSQRGSDMLSQEMEFGTLHSQE
MSQASISFSRMSESQMSTSYDIEPNMSQIAKDLNGLMLSEDSKI

> SVE_0430100

MAAKEDGLCLSENDDYEHMAAASQDSEYEENLDLDDIDSVIDNTNNKTDEGSDDEGEYDG
LLGPDFSKLPPHACRYCGIHNVNQVLQCSTCEKWFCNGKGITTGAHILQHMIRAQHREIN
FHKEAELGDSVLECYQCGVKNIFLLGFVPAKNDNVIVVLCRNCSSMATKSANWCADEWHC
LIEERSLLPWIAKVPTVKERIKSFRITTAQIKALEELWRVNPSADISDLHKPGLDEAVEP
VQRTYKDAYNYRRILAPLIRLEADYDKAAKEALKPTVSNVKWDYAKNKKILATFQIQELL
TSGIKLMIGDELKLKHNQTLLGTTWECHGHLIKVPDHHSEDFVLEFDESFHLPSAKRIIY
TVEFIWNATSYTRMYNALDTLAQKPNCVSSYIYEKLMGHPTSEALLNVKLPKSFNVPGLP
DLNHSQVNAVRQALRKPLTLIQGPPGTGKTVCSANLIYHLVHQRKGKILVCAPSNIAVDQ
LAEKLHQTKLKVIRLCAKGREDVQSPVAFLSLHNQLKTLTQDTDLQTLLRMKEEGGGLSA
TDESRLKIIIKAKEKVLLSKADVICCTCVCAADSRISFLKFKSVLIDECSQATEPEIMVS
VVKARSQLILVGDHCQLGPVVMCEKANAAGFSQTLFERLMLNGNVPIRLQVQYRMHPAMS
AFPSNVFYEGSLQNGVTSSDRILSNLTFQWPVPNKPIIFWHTDGTEELSPTGTSYLNRAE
AINVELLATMFLNSGVRPEQIGIITPYGGQRAYIVQHMHACGTLHYKLYNEIEVANVDAF
QGREKDLIIVSCVRSNENNGIGFLSDPRRLNVALTRSKYGLVIIGNAKVLSKNILWNHLL
LNYREQGCFMDGHINNLRPSQIPLPKERSLIEFFKKHTRFVSPALSMTEPVKLQTVDLTK
SYPTGMQNPNSFIFQNTHPSATYQPFVFRQPTLGQVNGFQNAIQSQCENTRQGVNKRTKN
NHSNSRSGSSYSQRGNDMLSQEMEYGTLQSQEMSQTSISFSRMSESQMSGPFDIEPNMSQ
IARDLNGLMLSEDSKI

C. elegans smg-6

> SRAE_2000102200

MSDASLSMSSDSTNKKVKRPDRPVYKPGMFTKRTLSTVNPISESSEKQKNSKENSNDVSR
NDYIDGVNSNGNQKSFKEKSEKNSRREYNRYNFNGNSGNKRGPRHSTGNGSNTSLNYTSG
GMSLDNSMQANLDNPKKGNFYNNRTHSKHSYNHPGHFQSNTLGGNRNHGTGYRRRNNSIR
SIKSEIVSNSKDDGTNFDAKSVCSSIILNETASISSYTCNNKMNYYASQAPSVNSLATNR
KENEISMDSLMMSLESFDWAACMEEENERQMQVTGSNNRIDEVDEEKYKNENEDTNKQDG
NVKKSIFERITVLPQSSLDTTNDYLNNKQNSQKNDWSGSSTRINKSDYDKRGSNSKLSKN
EFGSWSSLNRERLDKGNKNIKVRSKEDISPNKMKPNNKALKPPPSPRNVGLINKVDGYKS
VTGISITQIKGKPSKTVVKGNTLTQISPRKQSVESLNLQVTPPEFDGYEREISMNIKEIK
NRPKDVLQIIPSLIKNTLRLANEYKTHITRDIETTYKKQWELKMWKNGFYKVIDLFKQTD
VDKNLKSDFQMMYQNFLDAAILYYKELITLYENTYGIRFKEQVPWPYSTDLTNETFLECI
IVNEAPYYSLKSKIQITAITSVQRHYLSLSDLYRYKCMAHGLKSYEKAYEYCWLASQLSP
TVGRSYNSLGLISLYMILQRDPLRRRLDPIFRHLFSIMVSRDIRDQERYLEMIFFYIRSL
AAQFPHKGAKEAMLGTFSDVAGKVKNYNEECMNELFSKHLELNPSKAFENDKEIWRLPEI
RTNSRTWSFDLSMEGSLINGVNDFEIISNLQRLSSSKLYKFAITYLVHCAGILNSKIDME
SFDLYAEIGLICISALLSRNDSPLSCRQLIEITSFFIYYYYTSKNDETSYLHEQRSHSLQ
IIMSLFGIFLSVCSDNSSQLSSLIESKHDDLESVKKVLPVITIIYNFIKSDVFLEDLKKF
GESVFKTIKTNKFKINLLQYLIDIGNLLVTLRNKNILPSYEHLENKDRKRSSTDGVIVSL

PEEIFLLSFDKLFSQKIKVYEIYTHDEEKKKDKKVLGNIVRLNNILQTLEMLFVSTMDFI
NWDDKNERYWLVNSETDEECKTPTPEKIEENLNASNDNNMIENYRNRIIVEPHYIIPDTN
CFIDFSGTIEQLIRSRYFKVTLSYTVIKELRNLSKPVHSKPEVRTKPIKKSNLEADSNHN
EWVRKQAKRALKMIDEWFEKYSDYVISLTEDGLLQEELPPSSTVLPVISSINHGIVQKND
DRILQSVINLEKQFSLNESKKNASNSIKNVAMLTEDKALRIKACAEGIPCKSLSDFVMWLRM

> SPAL_0000476000

MSDAPISLPVDSTNKKVKRPDRPVYKPGMFTKRTLTTINPISESPEKQKNAKENTSDVSK
SDYIDGASNNYGSQKFFREKSEKSTRREYGRSNFNGNCGGKRGTRYFTGNGSNSSLNFVP
GGMPSDNNIQNLMDNPKKNNYYNNRTHPKHSYNNPGHFQSNTLGSNRNHGSGYRKRNNSI
RSIRSEIVPHSKHDGANFPSSSSNFDAKSVCSSIILNETASVSSYTCNNRLNCYASQAPS
VTSLTTNKRENEISMDSLMMSLESFDWAACMEEENERQMQVTGSSNRIDEVDEEKYKNEN
EDNNKQDVSVKKSIFDRITLLPQTSNDKSNDNVYSGKQNNQKNDWNGSSSRIDKSDYDRR
GSNNKLNKSEFGSWSSLNKERLDKVNKNIKVGSREDISPTKIKTNNKALKPPPSPRNTGP
INKVDGYKSVAGISISQIKGKLPKGVIKGNNFVQTSPKKQSVESLNSTPTPPEFEGYERE
ISMNIKEVKNRPKDILQIIPSLIKNTLRLANEYKLHITRDIETTYRKQWELKMWKNGFYK
VIDLFKQTDVDKNIKSDFQMMYQNFLDAAISFYKELITLYENTYGITFKEQVPWPYSTDL
CDESFLECIIVSEAPYYSSKSKLQVTAITSVQRHYLSLSDLYRYKCMAHGLKTYEQAYEY
CWLASQLSPAVGRSYNSLGLISLYMILQRDPLRRRLDLNFRHLYSIMVSRDIRDQERYSE
MIFFYIRSLAAQSPYKAAKEAMLGAFSDVNGKVKNYNEECMNELFSKYLELNPSKAFEND
KEIWRLPELRTNNRPWSFNLSLEGNLINGVNDFEIISNLQTLSTSNLYKFAITYLVHCAG
ILNSKIDMESFDMYAEIGLICTSALLSRHDSPLSCRKLIEITSFFIFYYASSKSDEASYL
QEQGSHSLRMIMSLFGIYLSVCSNNLNQLSSLITAKNDDLESVKKVLPVITIIYNLIKSE
IFLDDLKNCGELVFKTIKTNKFKINLLQNLIDIGNLLINLKNKNVLPSCIHLESKDRKRS
STDGVIISLPEEIFLLSFDKLFSQKIQVYKVYIQDNERKNNKKILGNIVRLNNILQTVEK
LFVCTMDFINWDSKIENYWLVNSEADEECKTPTPETVEKSITAKYDNNTVKEYENRIIVE
PQYIIPDTNCFIDFSGTIEQLIKSRYFKVTLSYTVIKELRNLSKPVHSKPENRIKTKRKS
NLEADSNHNEWVRKQAIKALKMIDEWSEKYSEYVISLTEDGLLQEELPTSSTLVPAISPV
NFSISQKNDDRILRSVINLQKQLSLDQEKKSIDNPIKSIAMLTEDKALRIKACAEGIPCKSLNDFVMWLRM

> SSTP_0000727200

MSDASLSISSDSTSKKIKRPDRPVYKPGMFTKRTLTTINPILESPEKQKNSKRDSDDVSK
NDYVDGALNSSNGNQKFSREKNEKNSRREFNRFTFNGNYDNKRGSRYSTGNGSNSSLNFN
PGNISSDSNVQTHLENSKKGNFYNNRTHSKHSYNYPGHFQSNTLGSNRNHGSGYRRRNNS
IRSIKSEIVSNSKDDKSNFDAKSVCSSIILNETASISSYNCNNKMNCYASQAPSVTSLAT
NKRENEISMDSLMMSLESFDWAACMEEENERQLQITGSTNRIDEVDEEKYKNENDDNNKQ
NENIKKSIFDRITVLPKSSINSSNDYLNNKQKSDWSGSNTRLNKSDYDKKGSSSKLNKNE
FGSWSSLNRERYDKGNKNNKIGSKEDILSNKIKTNNKTLKPPPSPRNIELVSKVDGYKSV
TGISITQIKGKSPKTVIKRNNLTQISPRKQSTESLNLQAILPEFDGYEREISMNIKEIKN
RPKDILQIIPSLIKNTFRLADEYKAHITRDIETTYKKQWELKMWKNGFYKVIDLFKQTDV
DKNIKSDFQMMYQNFLDAAILYYNELITLYENTYGIRFKEQVPWPYSTDLTNETFLECII
VNEAPYYALKSKLQVTAITSVQRHYLSLSDLYRYKCMAHGLKSYEKAYEYCWLASQLSPT
VGRSYNSLGLISLYMILQRDPLRRRLDPTFRHLFSIMVSRDIRDQEKYLEMIFYYIRSLA
AQFPHKGAKEAMLGTFSDIAGKVKSYNEECMNELFSKHLELNPSKVFENDKEIWRLPELR
TNSRAWSFDLSLEGNLINGINDFEIIGNLQRLSSSKLYKFAITYLVHCAGILNSKIDIES
FNMYAEIGLICISALLSRNDSPLSCRQLIEITSFFIFYYCTSKNDETSYLQEQRSHSLQM
IMSLFGIFLSICSNNSNQLSSLIAAKNDDLESVKKVLPVITIIYNFIKSDIFLEDLKNFG
ESVFKTIKTNKFKINLLQYLIDIGNLLVTLRNKNILPSYEHLENKDRKRSSTEGIIVSLP
EEIFLLSFDKIFSQKIKVYEVYTQDIEKKKNKKILGNIVRLNNILQTLEMLFVSTMDFIN
WDDKNERYWSVNLEIDEECKTPTLEIVENNLKILNDNIIIENYKNRVVVEPYYIIPDTNC
FIDFSGTIEQLIRSRYFKVTLSYTVIKELRNLSKPVHSKPEIRTKTIKKSNLEADSNHNE
WVRKQAIRALKMIDEWFEKYSNYVISLTEDGLLKEELPLSSTITPITSSINYGIVQKNDD
RILQSVINLEKQFLSDEGKRNVANPIKSIAMLTEDKALRIKACAEGIPCKSLTDFVMWLRM

> SVE_0454900

MSDASISLPVDSTNKKVKRPDRPVYKPGMFTKRTLTTINPISESPEKQKNAKENTSDVSK
NDYIDGASNNYGSQKFFREKSEKSTRREYGRSNFNGNCGGKRGTRYFTGNGSNSSLNFVP
GGMSSDNNIQNLLENTKKNNYYNNRTHPKHSYNNPGHFQSNTLGGNRNHGSGYRKRNNSI
RSIRSEIVSHSKHDGANFPSSSNFDAKSVCSSIMLNETSSVSSYTCNNRLNCYASQAPSV

```
TSLTTSKRENEISMDSLMMSLESFDWAACMEEENERQMQVTGSSNRIDEVDEEKYKNENE
DNNKQDVNAKKSIFDRITLLPQTSNDNYNDNVYNSKQNNQKNDWNGSSSKIDKSDYDKRG
SYNKLNKCEFGSWSSLNKERLDKVNKNTKVGSREDISPTKIKTNNKALKPPPSPRNTGPI
NKVDGYKSVAGISISQIKGKLPKCVIKGNNFAQTSPKKQSVESLNLTPTPPEFEGYEREI
SMNIKEVKNRPKDILQIIPSLIKNTLRLANEYKLYITRDIETTYRKQWELKMWKNGFYKV
IDLFKQTDVDKNIKSDFQMMYQNFLDAAISYYKELITLYENTYGITFKEQVPWPYSTDLC
DESFLECIIVSEAPYYSSKSKLQVTAITSVQRHYLSLSDLYRYKCMAHGLKTYEQAYEYC
WLASQLSPAVGRSYNSLGLISLYMILQRDPLRRRLDLNFRHLYSIMVSRDIRDQERYSEM
IFFYIRSLAAQSPYKAAKEAMLGAFSDVNGKVKNYNEECMNELFSKYLELNPSKAFENDK
EIWRLPELRANNRPWSFNLSLEGNLINGVTDFEIISNLQTLSTSNLYKFAITYLVHCAGI
LNSKIDMESFDMYAEIGLICTSALLSRHDSPLSCRKLIEITSLFIFYYASSKNDEASYLQ
EQGSHSLRMIMSLFGIYLSVCSNNLNQLSSLITAKNDDLESVKKVLPVITIIYNLIKSEI
FLEDLKNCGELVFMTIKTNKFKINLLQNLIDIGNLLINLKNKNVLPSCIHLESKDRKRSS
TDGVIISLPEEIFLLSFDKLFSQKIQVYKIYIQDSERKNDKKILGNIVRLNNILQTVEKL
FVCTNDFINWDSKIEIYWLVNSEADEECKTPTPETVEKRIDAKYDNNTAKEYENRIVVEP
HYIIPDTNCFIDFSGTIEQLIKSRYFKVTLSYTVIKELRNLSKPVHSKPENRVKTKRKSN
LEADSNHNEWVRKQAIKALKMIDEWSEKYSEYVISLTEDGLLQEELPTSSSLIPAVSPVN
FSISQKNDDKILRSVINLQKQFLLDQVKKNIDNPIKSIAMLTEDKALRIKACAEGIPCKSLNDFVMWLRM
```

C. elegans ego-1

> SRAE_1000097100

```
MITLENIRIIFKLFLNDSSNVEKLKIILNDCISESSTNLQKIGDVAYIDNHFLDSEMHVL
CKCNFGFLESGDFFEYLQLLKLKFKLLNINFAIGIEKFLNNYTSFEICNTNICVQNFGWG
TYYYPEIFYDHYNVDERWSNQYNDYNIKFKKFFNIKYQNIKLIYANFYHDIHKLNVFFSI
PPCNKDSQFKQTENTISKLTLDYRNINRVAISDIILSKKLKNYYIVTFNFWLFNPIKIET
YQEISESFDEKIKKYWKWNQVRTFCDDDNIIEVIHESSIFYLTLELSKNEMVNIIERFCT
LISRNLEFVCWKKINLSITSLVKKPLDDKILKKKLLKNGSFELSYLIETILSMGLLTKVD
LLISQEKRDTFIENVLSCYQENNKITLLALENIIKRIERMFFVKNICKMFQLIYKNESEN
EYLVDDFIEEDIDQNIFYVRKVIVTPTRTLFKIPTAMIGNRVMRQYDPTGEKMLKVIFRT
DNLRSTKEIGSDCILTEIINKYLDKGIIVGGFIYNFLGASNSQIRDGGCYFFRGSKYDMI
SIREGLGSIKQEAIPKMMGRLGQCFTQSFLAKNAIIANDKYVKDNDYFTPVWKDSGEKEY
CFSDGCGMISNEMGRKIVSSFKNIFNQSSTCYQFRFRGYKGVLVKYSTLDKVNQMAIDKK
ISIKNIGRINNDNLWTNDFSVDCVFRYSQCKFRGLLKDCQLEIVKSSKPQELSLNRPLIN
VLDQVSKLQSYECNKRICYRINELFEKHISSIISIFLHEKNAYETLSNMSLKYFGIRKLN
NHKLISFHKEIFFKNILKNYAIYQIKDNLKKLKIKIPTNLGRLMFGVIDETGSLEYGQVF
IQYSLNINSSSKKTKNKDDKKVHLGKVMITKSPTIVAGDVRIFEAVDVNLLHDLVDVIVF
PRDGPFPHTTEMAGSDLDGDEYSVIFDEELFFEYNMKPFDFDVGTSTNEVVNGIKDHKDF
DDRMKEFMLTYLTSDNVGVLASSHLIQSDFFGINSEVCKRIAIKHNIAIDFQKTGKFPQP
LTKEWENDIPPEVPSVVAEFFDGNISKMPSYKSSRLISQLYNRLNKLETLLESSDLLLQD
EEYTKNPLISINGWEKYNDLAMKYYIKYSTAIINLMDTFEIHDESELFCGYRINSNYNNI
NDITNRLSYQNINFLIQKKLTLIIYKIKTEILETFCPLEHFYDVIPDINDYENIKRILEN
PLSNYPKELEEFVVAGYNILYDSTKKDIIKIYSFPWIFWDVLKKIAFINYYNNCNIISLS
YPTFHDLLTKYIMEWYLSINEKDKVLKKLVTEDEELKICLEYIKCYKNLNILLTFLLSWS
KLNNLNIKIKEDKLSDLSEEEIKNSFDINSSIGGIGKHFLNILFILSSFKFMKLDHIYGW
EMDMECGYFLLQEHCYLIHQAAEKTINSLVFYHAFDILPQFKNKCNNLQDCKSYHSMYVY
LPKNMKCHESYIFDRVKNISGLEALKYRKENYYFNGCKETTLWIVTPVGTYDAYLKFKQF
IKVDVSTSFIISEKHNFPYVLGLKLYEKIINRPVVMS
```

> SRAE_2000026400

```
MNEKFETKIELKVLLGSKSKSEDIQLTLDFILFEQGKEKFQIGKLSDYVIKQDLLLGQQM
SVLLSCPFQIISPEFISILNLLTETLKPMSVYFFVTIGRFSKSHENYELMNKAVHIYKIG
WGNYYYPGTFIDHYNVTELWSKEYINDNIEFRRLYENNCGNISPIFADFYHDRQYMEIYF
AIPMCNQDCTIEQRKQSVAKIVLYYNKIIKMSINDIREDKKESGMYIASLYFWTMNPVSV
YYYQYVVAKIGEKERKFWEYKQTRSFIDENSSYVKEAIHESSIFYIQLRLSSNDLMNLIE
RFKSLTSKEVEFTNWRLLTFDRNFYVEKPFQNKECKKKIDNQSDFSLSYLIDAVFSRGFN
IKDQLLTSSKRRDDFLDMILKFYQENRKVTLLSLESFINKLDESIVVGDILNIFEWVYNN
ESKNVEITDAIKEHEKKEHYVYVRKIVITPTRKIYRPPELMMHNRMLRQFDPNGKKTLRI
LFRDDNKRPIKEVRNEYIIEQVLNDCLDNGLNVGGQLYNFLGSSNSQMRDGGCYFYRGTT
DEIIELRKNFGFIKQEVVPKMIARIGQCFTQALIAEKAIVQENRMLRDPDYETPRWIGSN
```

EKVGCYSDGCGMISYNMAENIFKSLNIFNLVSSCYQFRFRGYKGVLSVCPHLDKSNKLAE
KNKINLIGFARKNNSILKHEFAVDSVFRFSQLKFKGNSEDRHLEIVKSSQPSLLSLNRPL
LNVMDQVSKRQSYKCNKRICNRVHELFDVHVSSIRKCLLTERGAFEILSSMPIKCFGVNQ
LNNPKVVSFQVEPFFKSMINSYALFQVQKVLKKLKIQIPCNMGRTMFGIIDETGILEYGQ
VFVQYHENMNVMTNMNDINTKKIIHKGKVMITKNPTVVSGDVRIFEAVDVPELHDMVDVV
IFPRDGPRPHSDEMAGSDLDGDEYSVFFDESLFIDYNMPAFDFDAGKSVKDVKVGVKDEN
ELDIRMKEFIKDFLKTESIGTLASSHLMQSDFFGLDSEVCVKIAIKHNKALDFAKTGEFP
EQLTTRWEGNVPPETPKVVADIFEYRALKKPSYESSRLIGELFRRLHNLETLLSTSSSNF
ENDIQKKNPLFDITNWNEQYTLATKFYYRYAAAITSLQDTYGIDNEAELFSGFRLNVRNK
ITDNDDDDMSYFNTDLVIRRKLKLILYNFKIEILKNFGDIDKFFNDLPKEESNERIEIVL
EQPLAIYPETLKKFVIAAYHVSYDMVKCGAFNIYSFPWIFWDVLKKVGSLNLVRMENKQF
ITSKYFDSLLTNHINEYILSDTKKDSFEKFCHELKNNQQLKECYRYVKRYINLDKLLYFL
LEWAKEWNIIPKVNKHLLIVLFIQLLCGYYNINSIESYNFIDKISELTNEELMTPIDINS
IHGGSGRFCLDILSFLSSYQFKNLNHIFGWDMEMGCEYALLNFEWISIYNAAVETINNLA
FSHTFDALPQIIKKVCKYQSFPPMTVYLPKEVSICALTIFEKIKRISGLKKIHHRKNENS
YKSIKNVTEWVLTPIGTYESYIKFKGIVRPVIPTSINIIHEIDLAHYIAMKFFAKVNNVSIFNS

> SPAL_0001017300

MNNKNETKIELKIALKEKFNAKDIQDTLDFTLIDEGKEKFKICRLNDYVVKKDPLLGSQM
SVLLSLSSELASKEFLSFLNALKDMLKPMGHHFFVGIGRFPKNHVDFELTSKAVRVYKMG
WGNYYYPDTFIDHYYVKESWSNTYTRENKEYELLYEKYCGNVSPIFADFYHDRQYLEVHL
AIPRCNQDSPCEQKKRSVARIVLFYNKIIKLSINEIKESDNEKGTFIVNLSFWTANPVSI
YVSQYTIAKIGGKERPIYDFKQTRSFVDEDAEYVKESIHESSIFYIQVKLLGGELMNLIE
RFRSITSKEVEFTKWATLKRNSYVEKPFDNEECRKKIENEGSFSLSYLIDAIFSRGFVVK
DQLLSSAQRRDRFIDAIIECYRDNKKITLLSLEAFLNKLDELTVVSDIIEVFEWVYNYEL
RNLKITNAVKEHEKKEHYVYVRKIIITPTRRIYRAPELMMHNRMLRQFDPNGEKTLRILF
RDDNKRPIKEVRNDYILEQVISDCLDNGMNIGGQLHNFLGSSNSQMRDGGCYFYRGTRHD
IIELRKNFGSIKQEVVPKMMARIGQCFTQALIAEKAVVVESRMLKNPDYETTFWINKNEK
IGCFSDGCGMISYDMGEKILKSLNLFDRVSSCYQFRFRGYKGVLSVCPFLDKANKLAEND
KIILIDFGRKNDSRFKHEFPTDCVFRSSQLKFKGDSKDKHLEIVKSSQPSLLSLNRPLLN
VMDQVSKMQSYECNKRICNRVHELFDVHVSLIRKCLLTEKGAFEILSSMPLKCFGVSQLN
SPKVVSFQLEPFFKSMINSYALYQIQKVLKKLKIQIPCNMGRTMFGIIDETGILEYGQVF
IQYNEDMNIMTNINDIHPKRIIHKGKVMITKNPTVVSGDLRVFEAVDVPELHDLIDVVVF
PRDGPRPHSDEMAGSDLDGDEYSIFFDESLFIDYNMPAFDFDAGESVKSVQKGVKDEHDL
DEKMKAFIKDFLKTESIGTLASSHLMQSDFLGLDSEVCYQIAIKHNKALDFAKTGEFPKP
LTTRWNRSIPPETPRVVADIFEYRAMKKPSYESSRLVGELFRRLCNLETLLSTSSSALGS
GMQNKNKLYDIANWKEQEVLATRFYYRYAAAITSLQDTYGIDSEAELFSGFRLNVRNKIT
DSDDDDMSFYNTDLVIKEKLKMIVYNFKVEILKLFGDLNSFFSELPQEESKERIQMVLES
PLIIYPEDFKKFVVAAYHVSYNIVKRGDFNVYSFPWIFWDVLKKVGSLNSIKVEGKKFLT
PGYFDSSLTSYINLYISSEKKADFEEFCFILKTDPQLKECYRYVKRYENLEKLLYFLLEW
AKKWNIVSKINKKLLVVLFIQLLCGYYHGSSVETYNFLDKLSELTDEELNNSIDINSIQG
GTGRLCLDILSLLSSYQFKIRDYIFGWDMGMNCDFALLNFEWTCIYEAAVETINNLAFSH
TFESLPQLNKNVEVNCRIQSFPPITVYLPNGSSVYASTIFEKIRNMSGLLKVYHRKNENL
SRSIQNVSEWVITPIGTYESLMKFKSLVRPEIPTSMNITHEIDLAHYIAMKFYIKALDISIINS

> SPAL_0001386500

MMTLKINLKVFLDNNTDVKKIKNIIVDTLNEFPEKVKSNFCFEIIEKDFLNKELSLWCDY
SFESFKSGEFFNRFIEEFKIRLTIVRLHFTIGIGKLLEEYKSFKYVFNHDPCFNNFSSRL
TNEHINILKFGWGTCIYPGDFCTHFNFSQDSSKKFLRHGKNFNPNIEKVMKGFTPIYGNF
YHDLNKMEIFFATSPCKYDCDFVQVSNTIGKITIFYKNINRLSISNIEKSSNKANHFRVT
FNFWLLFPVRVDAYQQMRETKNEKDFTAWKLRRIRTFDDDLSTIQAIYESSVFYFRMEVP
LDVMINLIERLSSITNRVVEFVSWKEKSLPIKRFIRKPFDDEILENKLFKNGSFELEYFI
DALLSKGLFVKIHILTSTEQRNTFINTILDYYRKNSKITLLTIENLIKTLEENVFIKDIL
SIFERLYKIEVENEKIVDTINGEDKLCSNYVYIKKVIITPTRMLLKVPTLMVSNRVMRLF
TKKGRHFLKVIFKSDDNRPIIMIKNDFLLKQIVNECFVNGITVGGKKHNFFGASNSQMRE
DSCYFVDASIIEILNMKKELGSFTLESVSKMIARVAQCFTQSWNAENAIVGENEMKKGTE
YDNPEWIKSNKKTYCYTDGCGIISLAMAKNIASSFKDKYQAISSCYQFRFEGYKGVLATY
PILDKANKLNTENKITLFDNKNQLHFIWRPSQMKFRANNSLKNLEIVKASKPSEVSFNRP
FINVLDQVSRMQSIECNRRICNRIHELYDNHVTSIIEALLDEQSAFQTLSSMNLKYFGIK
ELKDTRMFSFQTEAFFKNLINVYASYQVNTLLNKFKIKIPSNLGRTMFGVGDESGCLEYG
QVFIQYNTDMNPKKDNANSGKKIHIGKVLVTKNPTVVSGDVRIFEAVDVEALHDLVDVIV

FPRDGPKPHTTEIAGSDLDGDEYSVIFDENLFLEYNMEAFDFDKEDSDKHVSVGVNSHME
FHAKQKEFMLDFLLANSVGVIATTHLIQSDFFGLDSEVCNRIAIKHNTAVDFQKSGSFPK
KLTDRWDKNVPPEINCAIPDYQESDNNKAISYKSNRLLGELYSRFIKLKTLLNSSDYLSQ
KVRHKKNPLVDIENWGKYYDMAKKYYSKYSSTLLGLMKTYAMEDESEIFCDYRVNLNTNF
VRTDKDIMLDYNINVVIKQKVETLIYNIKKEILETFYPLEYYYNVESGIDETEQLRLLLE
YPMIRYPDEIKEFVVAAYHVTYDSSKDEMFNIFSFPWIFWDVLKKVASMNSCFKRNNEAS
RVKSFSDLLTEHIMEWYSLTDEEDKDLYELFKDDIEMAGCLRYMKCYKNLDILLSFLLSW
SKSNGLTKKIKKGMLVALFIQCLTGHHYTANVKRHNFISKLSDLTDEELEHGFDINSYIG
GLGGRCLNIFSILKSYQFRKSNEIYGWDTDIDCGYTLTCEDCININDVAERTINSIAFSH
TFDILPQYEKNINKNYARTTSYPSVSIFLPSTMECTDDEFFSLLKKQSQLDMIKYRKNHD
YRSNNGTKSYLVTPVGTYESYIKFKNLIRIDQPTTFRITEGLDFTHILATKLYIRICSNLC

> SSTP_0000010600

MDEHPETKIELKVLIDKNYKPEDIQLALDFILIEQGEEKFKIGRLSEFTVKNDPLLGVQM
SVLLSYPFQIISREFISFLNLLKETLKPMSLYFFVKVGRFSKHFVDYELLDKAIRIHKIG
WGNFYYPGNFVDHYNVSEGWAKDYTDENVEFHRLYESYNRTISPIFVDFFHDRQYMEVSL
AIPKCNQDCVEEQKKRSVAKITLYYNKIIKISINEIKEDRKESGVYVVVNLNFWTINPVSI
YVYQYIVKKAGDKQRTFWDYKQTRSFIDEDAAYVRKAIHESSVFYMKVRIPGHKLMNLIE
RFRSITSKEVEFTNWKIVHFGMNSYIDGPFQDEECRKKIESQKSFSLAYLIDAIFSRGFV
VKDQLLCSPERRNIFIDVIIKYFKRNSKVTLLSLETFISKIDESVIVGDITNVFQWVYIN
ELSNVKISDAVKEHEKKEHYVYVRKIIITPTRRIYRSPELMMHNRMLRQFDPNGEKTLRV
LFRDDNKRPMKEVRNELIIEEILKDCLDNGLEVGGYLYNFLGSSNSQMRDGGCYFYRGTV
EEIIELRKNFGSIKHEAVPKMIARLGQCFTQALIAEKAVVEESRMLRDPDYETPYWMGKN
EKVVCYSDGCGMISHSMAEKILKSLKLFDRISSCYQFRFRGYKGVLSVCPFLDKANKLAE
DNKVILSGFGRKNDSRFKHEFPVDTVFRSSQSKFKGNSRDRCIEIVKSSQPSLLSLNRPL
LNVMDQVSKKQSYECNKRIRNRIHELFDVHVSSIRKCLLTEKGAFEVLSSMPIKCFGISQ
LNNPKVVSFQVEPFFKNMVNSYALFQVQKVLKKLKIQIPCNMGRTMFGIIDETGILEYGQ
VFVQYYEDMNVMTNTGDNHSKKIIHTGRVMITKNPTVVSGDLRIFEAIDVPELHDMVDVV
IFPRDGPRPHSDEMAGSDLDGDEYSVFFDESIFIDYNMPAFDFDAGVSVKNVQRGVKDEY
DFDEKMKEFIKDFLKIESIGTLASSHLMQSDFLGLDSEVCYQIAVKHNKALDFAKTGEFP
EQLTTRWIGSIPPETPKVVADIFEYRALKKPSYESSRLVGELFRRLYNLETLLSTSTTSL
DNVQNKNPLFDMPNWNEQYKLAIKLYYRYAAAITSLQDTYGIDSEAELFSGFRINIRNKI
TDSDDDDMSYYNTDLVIRKKLKLLLYNFKIEILNHFGGIDNFFKDLPQEESNERIQAVLE
TPLAVYPDSLKKFAVASYHISYDLVKRGVFNIYSFPWIFWDVLKKVGSINLAKKDKKDYI
TPGYMDALLTTHIEQYTLSELKIDSFKQFRLMLENDPQLKKCYIYVKRYINLDKLLFFLL
EWAKEWNIVPKVNEKLLIVLFIQLMCGYYHSNSIEPYIFIDKLSELTDEELNTVVDINVY
QGGSGRFCLDILSFLSSYQFKNLDYIFGWDMEMECDFSLLNFEWNYIYKAAVETINNLAF
SHTFEALPQIIKNYEGSCKVQSFPPMTVYLPNKITVHAVTIFEKIKKVSGLEKIHYRKNE
SISRGIQNVSEWILTPVGVYESYMKFKKLIRPEIPTSMNITHEVDLAHYIAMKFYIKAIDISFFNS

> SVE_0276300

MSFQNKELSLWCDYSFESFKSGEFFNNFIQEFKIRLTIVRLHFTIGIGKLLEEYESFKLI
NEHINVLKFGWGTCVYPGDFNTHFNFSQDLSKKFLKYGKNFNPNVEKVIKRFTSIYGNFY
HDLSRMEIFFVTSPCKHDCDFVQVSNTIGKITIPYKNINRLSISNIEKSLNKTNHFRVTF
NFWLLFPVRIDAYQQTRETKNERDFTAWKLRRIRTFDDDLSTIQAIYESSIFYLRMEVPL
NVLINLIERLSSTTKRVVEFVNWKEKSLPIKRFIGKPFDDEVLENKLFKNGSFELEYFID
ALLSKGLFVKIHILTSTEQRNTFINTILDYYKENSKITLLAIENLIKTLEENVFIKDILS
IFERLYKIEMENEKIVDTINYEDKLCSNYVYIKKVIITPTRILLKVPTLMVGNRVMRLFV
RKGRHFLKIIFKSDDNRPIIMIKNDFLLKQVVNEYFRNGIIVGGKKHNFFGASNSQMRED
SCYFVDASIIEIINMKKELGSFTLESVSKMIARVAQCFTQSWNAENAIVEGNEMKKGTEY
DNPGWIKSNKKTYCYTDGCGMISLTMAKQIASSFKDKYKDISSCYQFRYGGYKGVLATYP
ILDKVNKLNIENKITLFDNKNQLHFIWRPSQMKFRTGNSLKNLEIVKASKPSEVSFNRPF
INVLDQVSRMQSNECNRRICNRIHELYDNHVTSIIEALLDENNAFQALSSMNLKYFGIKE
LKDTRMFSFQTEAFFKNLVNVYASYQVNTLLNRFKIKIPSDLGRTMFGVADESGCLEYGQ
VFIQYNTNMNPKKDYANSSKKIHIGKVLVTKNPTVVSGDVRIFEAVDVEALHDLVDVIVF
PRDGPKPHTTEIAGSDLDGDEYSVIFDENLFLEYNMEAFDFDKEDSEKHVSIGVNSHMEF
HAKQKEFMLDFLLTNSVGVIATTHLIQSDFFGLESEVCNRIAIKHNTAVDFQKSGSFPKK
LTDSWDKNVPPEIYCAIPDYQESDNNKTISYKSNRLLGELYSRFIKLKTLLNSSDYSSQK
VRHKKNPLVYIENWEKYYDMAKKYYSKYSSTLLGLMRTYAMEDESEIFCGFRANLSVNFV
RTDKDLMLDYNINIVIKQKVETLIYNIKKEILETFHPLEYYYNVESGIDETEKLRLLLEC
PMIRYPDEIKKFIVAAYHVTYDSSKDDMFNIFSFPWIFWDALKKVVSMNSCFKRSSEESR

LKSFSDLLTEHIMKWYSLTDEEDKDLYELFKDDIEMAGCLRYMKCYKNLDILLSFLLSWS
KINGLTKKIKKGMLVTLFIQCLTGYHYTANIKRHNFISKLSDLTDEELEGNFDINSYIGG
LGGHCLNIFSILKSYQFRKSNEIYGWDTDIDCGYTLTCEDCININDVAERTINSIAFSHT
FDILPQYEKNVNKNYSIITSYPSVSIFLPGTMECTDDEFFNLLKKQSQLDMIKYRKNLDY
RGCNGTKSYLVTPIGTYESYIKFKNLIRIDQPTTFRITEGLDFTHILATKLYIRICSNLC

C. elegans rrf-3

> SRAE_1000029300

MSSRKNFKNCNRPKKISKEIYSKGRSSYFNKNEDNQCVQVLWFGLGSIIIGNTFACRSQY
FSGLNTYSSTELHHLETTCNYAAGNHQLLSYIHINNKKTTLRLDFCIPILPGTSKLNFQG
YRTYITYRNIRKIYIDLNDKNVKQTVIYIKIKQPLLFWKAIPNTSSTVKIMNTELCRHWR
RCFNWPSTEKCSGITSDIMVRSNVVAISIPKDNALSNVNSYRRGIYVKEGEDAFYSNIIC
EMLFILYKDYNIPIVNNKMERFFMKHIALMKPPVTKSFSINYSLEALNCRNFYITDQLFK
LDESGVPLFYLKVLQKMNISQVITEFALNRLLSFIDIHGEVNIIETFETLFTQGTKLMVN
DENQKNSKTFFTTIPKNCLYIRKVLVTPCKIVLLPPEVMMTNRVVRKFGENSCIRVVFRD
DDGTRIHIRGFYRTRSEEDEVNLISDFILNPLLNGLNIAGIRYNFLGWSNSQMRDHGAYF
FRDTEIINSTTGLSKIYKIIDVRRWMGDFTKCSSLPKMMARMGQCFTQTQPVICLKETDY
IVIDDIYGGYNYDGTKFCFSDGCGTISLKMASTLASILKLDYIPSVFQIRFKGFKGILSI
DNSIDNSGQAYSIIFRKSQLKFDIYNNDSEAFLEVVKYSMPAIACFNRQLIVILDQVGYK
QCIKRGSLISKTLMYYFMNEIRSLFSCFFFSDEAAKVAMDRCNSHINFYKLHESGIDLAK
EPFFRKIIGSIIKVANMNLMKLKLDFPKNCARTMYGLMDETGTLMPGQVFVQYSQNYFEP
NKNVILHQGKVMVTKHPCRSPGDVRILETVNVPALKHLVDVIVFPKYGWRPHPDEMAGSD
LDGDEYVVIFDKNLFFRNNEPASSYLSPSPRMCNEEISDLAMADFFINFIKHDYLGILSN
AHLVHADINGIFTPICTELAKKCSIAVDFCKTGVSTQQLQREEKSECAPDFLQPKSNKII
YESKRIIGIIFRKVNAFNHFTTLYQSIHDKAGPTILDEVFKMKMVDMLKYPKVYEKVKSS
YKEYIFRLEILMAEYGIDDEASIVSNSIVNLNRISDMEKMDFTFYHSEQIVDIRYKHIIS
IMRKQFFYDFGIEVYNLSKNYLLDDFKISKVMAAKARMWYTLAYTNSTDINEKPSLKSFA
WIIWDVLSNMKKNYNKINNEYSILQNDELCSENN

> SPAL_0000637300

MPPKKVARQLKKPRKVLKENCSKKKASYINKNDENQCIQTFWFGLGSIVVGNTFACRSQY
FSGLNTYSSTELHHLETTCNYAAGNHQLLSYIHINNKKSSLRLDFCIPTLPNTSKLNFQG
YRTYILYKNVSKIFVDLNDKNGKQTVIYFQIKRPLLFWKAIPNASSTIKIMNTELCRHWR
RCFSWESTEKCSGITPDIIAKSNTIAISVPKDNALSNVNSYKRGIYVKEGEDSFYSSILT
EMVFTLFKDYGVSIVNRKMEKFFMKHVALMKPPVTKNFAINYCLEALNCRNFYITDQLFK
LNESGVPLFYLKVLQKMDVSQHVTEFALNRLLSYIDIYGELDIIKTFEALFSQGTKLTMY
DENKKNNKSFFTTIPKNCLYIRKVLITPCRAVLLPPEVMMTNRVVRKFGENSCIRVVFRD
DDGGRIHIRGFYRTRTDEDDKNLISNFIINPLLNGINIAGVKYNFLGWSNSQMRDHGAYF
FKDTEIVNSVTGESKLYTITDIRKWMGDFTKCSSLPKMMARMGQCFTQTQPAICLKESDY
IIIDDVYGGYNNDGSRFCFSDGCGILSTKMAGALAAILKLDHVPSVFQIRFKGFKGILSV
DNTIGSNGETYSIAFRKSQLKFDISDSGGESFLEVVKYSMPAIACFNRQLIVILDQVGYK
QCIRRGSLISKSLIYYFMNEIRSLFSSLFFSDEAAKIAMDRCNSHINYSIIHECGIDLTT
EPFFREIIRSIIRVSTQSLMKLKLDFPKNCARTMYGVMDETGTLMPGQVFIQYSQNYFEP
SKNVILHQGKVMVTKHPCRSPGDVRILEAIYAPALKHLVDVIVFPKYGWRPHVDEMAGSD
LDGDEYVVIFDKNLFFRNNEPASSYESPAPTIYKEEISDIAMANFFLNFIKHDHLGVLSN
AHLIHADLNGIFSPICIDLAKKCSIAVDFCKTGISTQQLQRDEKSECVPDFLQPKSNKIS
YESKRLIGIIYRKVVAFNNFSTLYQSTNENTNPIVLDEVFKMKIVDTLKYPNIYEKVNNS
YKEYVFRMQMLMTEYGIDDEASIVSNSVTTLNRISDMEKMDFTFYHSEKIVDIRYKHIVS
LMREQFFSDFGNEIYKLSKNCLPEDVAISKVMSAKARMWYTMAYTNQENIDVNMKSFAWI
IWDVLARMKKKFNQNNDQYGNHHRDEFYDENY

> SSTP_0000447900

MSSKKIFKNSNKSKXIVKDNFSKGRSSYFTKNDDNQCVQILWFGLGSIIIGNTFACRSQY
YSGLNTYSSTELHHLETTCNYAAGNHQLLSYIHINNKKLTLRLDFCIPIPPGTSKLNFQG
YRTYIGYKNIKKIYVDLNDRNSKQTIIYFKLKQPLLFWKAIPNTSSAVKIMNTELCRHWR
RCFNWPGTEKCSGITSDIIARSNVIAISIPKDNALEGVNSYRRGIYVKEGEDAYYSNILC
EMLFTLYKDYNIPVINKKIKRFFMKHVVLMKPPMTKSFSINYSLEAINCRNFYITDQLFK
LHENGAPLFYYKVLQKMNISQGVTEFALNRLLSFIDIYGEIDILETFEILFTQGIKLMGS

NDNPKNSTTFFTTIPKNCLYIRKVLITPCKKVLLPPEVMMTNRVVRKFGENSCIRVIFRD
DDGGRIHIRGFYRTRSDEDEVNLISDFIFNPLLNGIRIAGEEYNFLGWSNSQMRDHGAYF
FKNTEIKNPTTGEGKLYTVTDVRRWMGDFTKCSSLPKMMARMGQCFTQTQPVISLKEDDY
IVIDDVYGGYNYDGTRFCFSDGCGTISLKMASTLASILKLDYVPSVFQIRFKGFKGILSI
DISIDNNNVSYSIVFRKSQLKFDIHNNNNESFLEVVKYSMPAIACFNRQLIVILDQVGYK
QSIKVGSLISKTLMYYFMNEIRSLFSCLFFSDEAAKVSMDRCNSHINFYKIHECGINLST
ESFFRKIIKSIIRVANINLMKLKLDFPKNCARTMYGLMDETGTLMPGQIFIQYSENYFEP
NKNIILHKGKVMVTKHPCRSPGDVRILEAIDVPSLKHLVDVVVFPKYGWRPHSDEMAGSD
LDGDEYVVIFDKNLFFQENEIASSYITSSPKIYNEEISNLAMANFFINFVKNDYLGILSN
AHLVHADISGIFSPICIELARKCSVAVDFCKTGISTQQLQRDEKSECAPDFLQPKSNKVV
YESKRLIGIIYRKVAAFNHFITLYQNIHNDSGPIIMDEVFKMKMVDMIRYPDVYEKVNDS
YKEYIFRLEILMAEYGIDDEASIVSNSIVNINRISDMEKVDFTFYHSERIVDIRYKHIIS
IMRKQFFYDFGIEIYNLSKNYLPDDFKISKVMAAKARMWYTIAYSAPKNKEDKGPMQSFA
WIIWDVLVNIKNRYNQIDKEYGDMDNDEFDNENY

> SVE_0019900

MPPKKVARHLKKPRKVLKENCSKKKASYINKNDENQCIQTFWFGLGSIVVGNTFACRSQY
FSGLNTYSSTELHHLETTCNYAAGKNINFRHTKISTIGNHQLLSYIHINNKKSSLRLDFC
IPTLPNTSKLNFQGYRTYILYKNVSKIFVDLNDKNGKQTVIYFQIKRPLLFWKAIPNASS
TIKIMNTELCRHWMRCFSWQSTEKCSGITPDIIAKSNTIAISVPKDNALSNVNSYKRGIY
VKEGEDSFYSSILTEMVFTLFKDYGVSIVNRKMEKFFMKHVAVMKPPVTKNFAINYCLEA
LNCRNFYITDQLFKLSESGVPLFYLKVLQKMDISQHVTEFALNRLLSYIDIYGELDIIKT
FETLFSQGTKLTMYDENKKNNKKFFTTIPKNCLYIRKVLITPCRAVLLPPEVMMTNRVVR
KFGENSCIRVVFRDDDGGRIHIRGFYRTRTDEDDKNLIANFIINPLLNGINIAGVKYNFL
GWSNSQMRDHGAYFFKDTEIVNSVTGESKLYTITDIRKWMGDFTKCTSLPKMMARMGQCF
TQTQPAICLKESDYIIIDDVYGGYNNDGSRFCFSDGCGILSTKMAGALAAILKLDYVPSV
FQIRFKGFKGILSVDNNIGNNGETYSIAFRKSQLKFDSSDSSGESFLEVVKYSMPAIACF
NRQLIVILDQVGYKQCIRRGSLISKSLIYYFMNEIRSLFSSLFFSDEAAKVAMDRCNSHI
NYSIINECGIDLTIEPFFREIIRSIIKVSTQSLMKLKLDFPKSCARTMYGVMDETGTLMP
GQVFIQYSQNYFEPSKNVVLHQGKVMVTKHPCRSPGDVRILEAIYAPALKHLVDVIVFPK
YGWRPHVDEMAGSDLDGDEYVVIFDKNLFFQNNEPASSYESPAPTIYKEEISDVAMANFF
LNFIKHDHLGLLSNAHLIHADLNGIFSPICIDLAKKCSIAVDFCKTGISTQQLQRDEKSE
CVPDFLQPKSNKISYESKRLIGIIYRKVVAFNNFSILYQSTNENTNPIVLDEVFKMKMVD
MFKYPNVYEKVNNSYKEYVFRMEMLMTEYGIDDEASIISNSVTTLNRISDMEKMDFTFYH
SEKIVDIRYKHIISLMREQFFSDFGNEIYKLSKNCLPEDVTISKVMSAKARMWYSIAYTN
QENINVNMKSFAWIIWDVLARMKKKFNQKNDQYGNNYRDEFYDENY

C. elegans rrf-1

> SRAE_1000097100

MITLENIRIIFKLFLNDSSNVEKLKIILNDCISESSTNLQKIGDVAYIDNHFLDSEMHVL
CKCNFGFLESGDFFEYLQLLKLKFKLLNINFAIGIEKFLNNYTSFEICNTNICVQNFGWG
TYYYPEIFYDHYNVDERWSNQYNDYNIKFKKFFNIKYQNIKLIYANFYHDIHKLNVFFSI
PPCNKDSQFKQTENTISKLTLDYRNINRVAISDIILSKKLKNYYIVTFNFWLFNPIKIET
YQEISESFDEKIKKYWKWNQVRTFCDDDNIIEVIHESSIFYLTLELSKNEMVNIIERFCT
LISRNLEFVCWKKINLSITSLVKKPLDDKILKKKLLKNGSFELSYLIETILSMGLLTKVD
LLISQEKRDTFIENVLSCYQENNKITLLALENIIKRIERMFFVKNICKMFQLIYKNESEN
EYLVDDFIEEDIDQNIFYVRKVIVTPTRTLFKIPTAMIGNRVMRQYDPTGEKMLKVIFRT
DNLRSTKEIGSDCILTEIINKYLDKGIIVGGFIYNFLGASNSQIRDGGCYFFRGSKYDMI
SIREGLGSIKQEAIPKMMGRLGQCFTQSFLAKNAIIANDKYVKDNDYFTPVWKDSGEKEY
CFSDGCGMISNEMGRKIVSSFKNIFNQSSTCYQFRFRGYKGVLVKYSTLDKVNQMAIDKK
ISIKNIGRINNDNLWTNDFSVDCVFRYSQCKFRGLLKDCQLEIVKSSKPQELSLNRPLIN
VLDQVSKLQSYECNKRICYRINELFEKHISSIISIFLHEKNAYETLSNMSLKYFGIRKLN
NHKLISFHKEIFFKNILKNYAIYQIKDNLKKLKIKIPTNLGRLMFGVIDETGSLEYGQVF
IQYSLNINSSSKKTKNKDDKKVHLGKVMITKSPTIVAGDVRIFEAVDVNLLHDLVDVIVF
PRDGPFPHTTEMAGSDLDGDEYSVIFDEELFFEYNMKPFDFDVGTSTNEVVNGIKDHKDF
DDRMKEFMLTYLTSDNVGVLASSHLIQSDFFGINSEVCKRIAIKHNIAIDFQKTGKFPQP
LTKEWENDIPPEVPSVVAEFFDGNISKMPSYKSSRLISQLYNRLNKLETLLESSDLLLQD
EEYTKNPLISINGWEKYNDLAMKYYIKYSTAIINLMDTFEIHDESELFCGYRINSNYNNI
NDITNRLSYQNINFLIQKKLTLIIYKIKTEILETFCPLEHFYDVIPDINDYENIKRILEN

PLSNYPKELEEFVVAGYNILYDSTKKDIIKIYSFPWIFWDVLKKIAFINYYNNCNIISLS
YPTFHDLLTKYIMEWYLSINEKDKVLKKLVTEDEELKICLEYIKCYKNLNILLTFLLSWS
KLNNLNIKIKEDKLSDLSEEEIKNSFDINSSIGGIGKHFLNILFILSSFKFMKLDHIYGW
EMDMECGYFLLQEHCYLIHQAAEKTINSLVFYHAFDILPQFKNKCNNLQDCKSYHSMYVY
LPKNMKCHESYIFDRVKNISGLEALKYRKENYYFNGCKETTLWIVTPVGTYDAYLKFKQF
IKVDVSTSFIISEKHNFPYVLGLKLYEKIINRPVVMS

> SRAE_2000026400

MNEKFETKIELKVLLGSKSKSEDIQLTLDFILFEQGKEKFQIGKLSDYVIKQDLLLGQQM
SVLLSCPFQIISPEFISILNLLTETLKPMSVYFFVTIGRFSKSHENYELMNKAVHIYKIG
WGNYYYPGTFIDHYNVTELWSKEYINDNIEFRRLYENNCGNISPIFADFYHDRQYMEIYF
AIPMCNQDCTIEQRKQSVAKIVLYYNKIIKMSINDIREDKKESGMYIASLYFWTMNPVSV
YYYQYVVAKIGEKERKFWEYKQTRSFIDENSSYVKEAIHESSIFYIQLRLSSNDLMNLIE
RFKSLTSKEVEFTNWRLLTFDRNFYVEKPFQNKECKKKIDNQSDFSLSYLIDAVFSRGFN
IKDQLLTSSKRRDDFLDMILKFYQENRKVTLLSLESFINKLDESIVVGDILNIFEWVYNN
ESKNVEITDAIKEHEKKEHYVYVRKIVITPTRKIYRPPELMMHNRMLRQFDPNGKKTLRI
LFRDDNKRPIKEVRNEYIIEQVLNDCLDNGLNVGGQLYNFLGSSNSQMRDGGCYFYRGTT
DEIIELRKNFGFIKQEVVPKMIARIGQCFTQALIAEKAIVQENRMLRDPDYETPRWIGSN
EKVGCYSDGCGMISYNMAENIFKSLNIFNLVSSCYQFRFRGYKGVLSVCPHLDKSNKLAE
KNKINLIGFARKNNSILKHEFAVDSVFRFSQLKFKGNSEDRHLEIVKSSQPSLLSLNRPL
LNVMDQVSKRQSYKCNKRICNRVHELFDVHVSSIRKCLLTERGAFEILSSMPIKCFGVNQ
LNNPKVVSFQVEPFFKSMINSYALFQVQKVLKKLKIQIPCNMGRTMFGIIDETGILEYGQ
VFVQYHENMNVMTNMNDINTKKIIHKGKVMITKNPTVVSGDVRIFEAVDVPELHDMVDVV
IFPRDGPRPHSDEMAGSDLDGDEYSVFFDESLFIDYNMPAFDFDAGKSVKDVKVGVKDEN
ELDIRMKEFIKDFLKTESIGTLASSHLMQSDFFGLDSEVCVKIAIKHNKALDFAKTGEFP
EQLTTRWEGNVPPETPKVVADIFEYRALKKPSYESSRLIGELFRRLHNLETLLSTSSSNF
ENDIQKKNPLFDITNWNEQYTLATKFYYRYAAAITSLQDTYGIDNEAELFSGFRLNVRNK
ITDNDDDDMSYFNTDLVIRRKLKLILYNFKIEILKNFGDIDKFFNDLPKEESNERIEIVL
EQPLAIYPETLKKFVIAAYHVSYDMVKCGAFNIYSFPWIFWDVLKKVGSLNLVRMENKQF
ITSKYFDSLLTNHINEYILSDTKKDSFEKFCHELKNNQQLKECYRYVKRYINLDKLLYFL
LEWAKEWNIIPKVNKHLLIVLFIQLLCGYYNINSIESYNFIDKISELTNEELMTPIDINS
IHGGSGRFCLDILSFLSSYQFKNLNHIFGWDMEMGCEYALLNFEWISIYNAAVETINNLA
FSHTFDALPQIIKKVCKYQSFPPMTVYLPKEVSICALTIFEKIKRISGLKKIHHRKNENS
YKSIKNVTEWVLTPIGTYESYIKFKGIVRPVIPTSINIIHEIDLAHYIAMKFFAKVNNVSIFNS

> SPAL_0001017300

MNNKNETKIELKIALKEKFNAKDIQDTLDFTLIDEGKEKFKICRLNDYVVKKDPLLGSQM
SVLLSLSSELASKEFLSFLNALKDMLKPMGHHFFVGIGRFPKNHVDFELTSKAVRVYKMG
WGNYYYPDTFIDHYYVKESWSNTYTRENKEYELLYEKYCGNVSPIFADFYHDRQYLEVHL
AIPRCNQDSPCEQKKRSVARIVLFYNKIIKLSINEIKESDNEKGTFIVNLSFWTANPVSI
YVSQYTIAKIGGKERPIYDFKQTRSFVDEDAEYVKESIHESSIFYIQVKLLGGELMNLIE
RFRSITSKEVEFTKWATLKRNSYVEKPFDNEECRKKIENEGSFSLSYLIDAIFSRGFVVK
DQLLSSAQRRDRFIDAIIECYRDNKKITLLSLEAFLNKLDELTVVSDIIEVFEWVYNYEL
RNLKITNAVKEHEKKEHYVYVRKIIITPTRRIYRAPELMMHNRMLRQFDPNGEKTLRILF
RDDNKRPIKEVRNDYILEQVISDCLDNGMNIGGQLHNFLGSSNSQMRDGGCYFYRGTRHD
IIELRKNFGSIKQEVVPKMMARIGQCFTQALIAEKAVVVESRMLKNPDYETTFWINKNEK
IGCFSDGCGMISYDMGEKILKSLNLFDRVSSCYQFRFRGYKGVLSVCPFLDKANKLAEND
KIILIDFGRKNDSRFKHEFPTDCVFRSSQLKFKGDSKDKHLEIVKSSQPSLLSLNRPLLN
VMDQVSKMQSYECNKRICNRVHELFDVHVSLIRKCLLTEKGAFEILSSMPLKCFGVSQLN
SPKVVSFQLEPFFKSMINSYALYQIQKVLKKLKIQIPCNMGRTMFGIIDETGILEYGQVF
IQYNEDMNIMTNINDIHPKRIIHKGKVMITKNPTVVSGDLRVFEAVDVPELHDLIDVVVF
PRDGPRPHSDEMAGSDLDGDEYSIFFDESLFIDYNMPAFDFDAGESVKSVQKGVKDEHDL
DEKMKAFIKDFLKTESIGTLASSHLMQSDFLGLDSEVCYQIAIKHNKALDFAKTGEFPKP
LTTRWNRSIPPETPRVVADIFEYRAMKKPSYESSRLVGELFRRLCNLETLLSTSSSALGS
GMQNKNKLYDIANWKEQEVLATRFYYRYAAAITSLQDTYGIDSEAELFSGFRLNVRNKIT
DSDDDDMSFYNTDLVIKEKLKMIVYNFKVEILKLFGDLNSFFSELPQEESKERIQMVLES
PLIIYPEDFKKFVVAAYHVSYNIVKRGDFNVYSFPWIFWDVLKKVGSLNSIKVEGKKFLT
PGYFDSSLTSYINLYISSEKKADFEEFCFILKTDPQLKECYRYVKRYENLEKLLYFLLEW
AKKWNIVSKINKKLLVVLFIQLLCGYYHGSSVETYNFLDKLSELTDEELNNSIDINSIQG
GTGRLCLDILSLLSSYQFKIRDYIFGWDMGMNCDFALLNFEWTCIYEAAVETINNLAFSH

```
TFESLPQLNKNVEVNCRIQSFPPITVYLPNGSSVYASTIFEKIRNMSGLLKVYHRKNENL
SRSIQNVSEWVITPIGTYESLMKFKSLVRPEIPTSMNITHEIDLAHYIAMKFYIKALDISIINS
```

> SPAL_0001386500

```
MMTLKINLKVFLDNNTDVKKIKNIIVDTLNEFPEKVKSNFCFEIIEKDFLNKELSLWCDY
SFESFKSGEFFNRFIEEFKIRLTIVRLHFTIGIGKLLEEYKSFKYVFNHDPCFNNFSSRL
TNEHINILKFGWGTCIYPGDFCTHFNFSQDSSKKFLRHGKNFNPNIEKVMKGFTPIYGNF
YHDLNKMEIFFATSPCKYDCDFVQVSNTIGKITIFYKNINRLSISNIEKSSNKANHFRVT
FNFWLLFPVRVDAYQQMRETKNEKDFTAWKLRRIRTFDDDLSTIQAIYESSVFYFRMEVP
LDVMINLIERLSSITNRVVEFVSWKEKSLPIKRFIRKPFDDEILENKLFKNGSFELEYFI
DALLSKGLFVKIHILTSTEQRNTFINTILDYYRKNSKITLLTIENLIKTLEENVFIKDIL
SIFERLYKIEVENEKIVDTINGEDKLCSNYVYIKKVIITPTRMLLKVPTLMVSNRVMRLF
TKKGRHFLKVIFKSDDNRPIIMIKNDFLLKQIVNECFVNGITVGGKKHNFFGASNSQMRE
DSCYFVDASIIEILNMKKELGSFTLESVSKMIARVAQCFTQSWNAENAIVGENEMKKGTE
YDNPEWIKSNKKTYCYTDGCGIISLAMAKNIASSFKDKYQAISSCYQFRFEGYKGVLATY
PILDKANKLNTENKITLFDNKNQLHFIWRPSQMKFRANNSLKNLEIVKASKPSEVSFNRP
FINVLDQVSRMQSIECNRRICNRIHELYDNHVTSIIEALLDEQSAFQTLSSMNLKYFGIK
ELKDTRMFSFQTEAFFKNLINVYASYQVNTLLNKFKIKIPSNLGRTMFGVGDESGCLEYG
QVFIQYNTDMNPKKDNANSGKKIHIGKVLVTKNPTVVSGDVRIFEAVDVEALHDLVDVIV
FPRDGPKPHTTEIAGSDLDGDEYSVIFDENLFLEYNMEAFDFDKEDSDKHVSVGVNSHME
FHAKQKEFMLDFLLANSVGVIATTHLIQSDFFGLDSEVCNRIAIKHNTAVDFQKSGSFPK
KLTDRWDKNVPPEINCAIPDYQESDNNKAISYKSNRLLGELYSRFIKLKTLLNSSDYLSQ
KVRHKKNPLVDIENWGKYYDMAKKYYSKYSSTLLGLMKTYAMEDESEIFCDYRVNLNTNF
VRTDKDIMLDYNINVVIKQKVETLIYNIKKEILETFYPLEYYYNVESGIDETEQLRLLLE
YPMIRYPDEIKEFVVAAYHVTYDSSKDEMFNIFSFPWIFWDVLKKVASMNSCFKRNNEAS
RVKSFSDLLTEHIMEWYSLTDEEDKDLYELFKDDIEMAGCLRYMKCYKNLDILLSFLLSW
SKSNGLTKKIKKGMLVALFIQCLTGHHYTANVKRHNFISKLSDLTDEELEHGFDINSYIG
GLGGRCLNIFSILKSYQFRKSNEIYGWDTDIDCGYTLTCEDCININDVAERTINSIAFSH
TFDILPQYEKNINKNYARTTSYPSVSIFLPSTMECTDDEFFSLLKKQSQLDMIKYRKNHD
YRSNNGTKSYLVTPVGTYESYIKFKNLIRIDQPTTFRITEGLDFTHILATKLYIRICSNLC
```

> SSTP_0000821100

```
MPTSQSIKLIFKLFLNDNSDVEKLKTTLLECISKSSSNIQQLGDFTYIDDPFLDPKLHVL
CRCTFNYLTSGDFFQYLYLINVKFKLIKANFSIGLEKFLNNHTSFRICDSFIRVRSFGWG
TYSYPEIFYNHYNVDEGWSNKYTKQNTKFKELLNRSNLRNKPIHVNFYHDTQKLDVFFAI
PPCNKDSQFKQSKNTICRLTLQYENINRISISDVSLSKELKNYYIVTFNFWLFNPIKIDV
CQELSTSISGKEKNYWGWRQVRTFYDESNIIEIIHESLIFYLTLQLHKNELINIIERFCT
LINKNLEFVYWRRVDLSITSLIKKPFDDKELKEKLIKNGSFALSYLIDSFLSKGLVIKVH
LLISQEVRDTFMEHILSCYQENSKVTLLAFEVFLKKIEGMLFIKDAHKMFQLIYKNELKN
QSLTDDLFREDNDRNIYYIRKIIITPTRILFRIPTPMIGNRVMRQYDPKGEKMIKVIFRT
DNYRPTKEIINGLILTEIINKYLDNGIAVGGFIYNFLGASNSQIRDGGCYFFRGSKYDMI
TIRESLGSIKQEAIPKMMGRLGQCFTQSFLAKNAIVENDKYIKDSDYYTPIWKDSNEKRY
CFSDGCGMISEEMAGKVISSFDEIFDQESTCYQFRFRGYKGVLVTYPILDKVNQLAVSNK
ITLQNIGKINNDNLWTNDFSVDCIFRYSQCKFRGLLKDCQLEIVKASKPQELNLNRPLIN
VLDQVSKLQSYECNKKICYRINELFENHILSIISIFLNEKNACETLLNMPLKYIINIKEL
NNFKLISFQKELFFKNILINYAVHHIENRLKKLKIKIPTNLGRSMFGVIDETGSLEYGQV
FIQYSLNINSTSKKTKNSIDKKIHLGRVMVTKNPTVVSGDVRIFEAVDVKLLHHLVDVIV
FPRDGPLPHTTEMAGSDLDGDEYSIIFDEELFFDYNMEPFDFDAGTSVNEVTKGIKDHKD
FDDRMKEFMTTYLTSENIGVLALSHLIQSDFFGLHSEVCKRIAIKHNIAIDFQKTGEFPR
PLTKEWDNNIPPEVPSVVAEFFDGNMSKKPSYRSSRLINQLYNRLNNLEIFLQSSDLLLQ
KEEYKRNPLIFIEGWEEYIDEALKYYIKYSTSIMNLMDMFGIYNEAELFSGYKIINSYNT
INNISEKLGYQNVNYLIQEKLGLIIYKIKAEILETFHSLDHFYNIIPEMDDSNKVKVVLE
SPLLYYPKELKKFVVAAYNILSDSTKTDMFKIYSFPWIFWDVLKKIVSINSCDDSSIITS
LPYDTFNDSFTKYIIKWYSSTIERDQKLMKLIMEDDEVKVCLEYIKCYKNLNILLTFLLS
WSKQHNLTLKIKDGMLLVLFIQCLCGFFNEYSNLSGGFIDKLSELSDEEIRNGFDINLSS
GGLGRHCLNIFFILSSFRFRMLDHVYGWDMGMKCGYMLFQEDCYLIQQAAEKTINNLVFY
HTFDILPQIENKDKSLESYKSYHYIYVYLPKNMKYNELYIFDRIKQISGLEELKYRQERS
YFYGCKETKLWIVTPVGTYDSYIKFKQLIKVDVPTNFVMTERLNFPYILGRKLYEKIINHSLIVI
```

> SVE_0507500

MNNNNETKIELKIALKEKFIVEDIQDTLDFTLIGEGKEKFKICRLSEYVVKKDPLLGSQM
TVLLLLSSELASKEFLSFLNALKDMLKPMGHHFFVGIGRFPKYHVDFKLTSKAVRIYKMG
WGNYYYPDTFIDHYHVKESWSCNYIKENKEYELLYEKYCGNVSPIFADFYHDRQYLEVHL
AIPKCNQDSPCEQRKRSVAKIVLFYNKIIKLSINEIKENDNEKGTYIVNLSFWTANPVSI
YVKQYTIIKIGRNEIPCFEFKQTRSFVDEDAEYVKESIHESSIFYIQVKLSGGGLMNLIE
RFRSITSKEVEFTKWTTLKRNSYVEKPFENEECRNKIENEGSFSLSYLIDAIFSRGFVIK
DQLLSSAQTRDRFIDAIIECYRENEKITLLSLETFLSKLDELTVVSDIIKVFEWVYNYEL
RNIKITDAVKDHEMKEHYVYVRKIIITPTRRIYRAPELMMHNRMLRQFDPNGEKTLRILF
RDDNKRPIKEVRNDYILEKVISDCLDNGMNIGGQLHNFLGSSNSQMRDGGCYFYRGTRHD
IIELRKNFGSIKQEVVPKMMARIGQCFTQALIAEKAVVAENRMLKNTDFETTSWINKNEK
IGCFSDGCGMISYDTGEKILKSLNLFDRVSSCYQFRFRGYKGVLSVCPFLDKANILAEND
KITLIDFGRKNDSRFKHEFPTDCVFRSSQLKFKGDSKDKHLEIVKSSQPSLLSLNRPLLN
VMDQVSKMQSYECNKRICNRVHELFDVHVSMIRKCLLTEKGAFEILSSMPLKCFGVSQLN
SPKVVSFQLEPFFKSMINSYALYQIQKVLKKLKIQIPCNMGRTMFGIIDETGILEYGQVF
IQYNEDMNIMTNINDIHPKRIIHKGKVMITKNPTVVSGDLRVFEAVDVPELHDLVDVVVF
PRDGPRPHSDEMAGSDLDGDEYSIFFDETLFIDYNMPAFDFDAGESVKSVQKGVKDEHDL
DEKMKAFIKDFLKTESIGTLASSHLMQSDFLGLDSEVCHQIAIKHNKALDFAKTGEFPKQ
LTTKWNRSVPPETPRVVADIFEYRAMKKPSYESSRLVGELFRRLCNLETLLSTSSSALGS
GMQNKNKLYDIANWREQYNLAKKFYYRYAAAITNLQDTYGIDSEAELFSGFRLNVRNKIT
DSDDDDMSFYNTDLVIKEKLKMVVYNFKVEILKMFGKLNSFFSELPQEESKERIQMVLES
PLIIYPEDLKKFVVAAYHVSYDLVKRGDFNVYSFPWIFWDVLKKVGSLNSIKVEGKQFIT
PGYFDSLLTSHIDSFTSSKQKNSDFEGFCFILETDPQLKECYRYVKRYTNLNKLLYFLLE
WSKEWNIVSKVNERLLVVIFIQLLCGYYHGSSVETYNFLDKLSELTDEELNNSIDINSIQ
GGTGRLCLDILSLLSSYQFKIRDYIFGWDMEMDCDFALLNFEWTCIYEAAVETINNLAFS
HTFESLPQIDKNVEVNCRIQSFPPITVYLPNGGSVYASTIFEKIRNMSGLLKVHHRRNEN
LSRSIQNVSEWVITPVGTYESLMKFKSLVRPEIPTSMNITHEIDLAHYIAMKFYIKALDISIINS

C. elegans rsd-3

> SRAE_X000072500

MEKKNEDNVNVNANLESDSEDDKDNTDERSIKLVLIGDGCSGKTSICNQLCKKKFSKKYT
QTYGVDFYSKKITLPNNIEVLLQIWDIGGQSVASPLLEKYLYGVQGVLLIYDVTNSTSFA
NLQDWITVTKKITKNYEKHVHMSLVGNKTDLEHRRAVRVEKHTKLAEQYGMSSHYVSAKT
GDSVALAFRQTAADIFGIQLSKIDMESDITIVQAPVAVPTEKELKEVAAKNALANQNNSAACRIQ

> SPAL_0001557000

MANLFSGLASITKTVSDALNNPEVKKFQDQVTGYVMNFSDAEIKVRNATNDEGWGPTNQQ
CDEIASMTFQYDLCTEASEMLFKRMMENSKGTWRRTYKSLRLVDHLLKHGSDRFIRYTRE
HSTQIRGLQNFHYIDEKGKDQGINIRVKAKAIIQLLQDEDLLMDERKKAKTMNRDKYVGY
SKEDMIHMSGSSSMSGFGSDNYNKYSDSFSNVTKSQSMNDGVKTEANLFNFPDDKDIDHT
ELGIPDTPTNDDDFGDFESPAKIPDVPKKNEIDLFGDIVPIAPPPSGITSPAFSPKTKVI
FESVKQPDVTNDLISLMDDLNVTNPPPSPKTSINVFSATRMDGDTLSLPQKNEPPKEYVN
NLLDFDFNKPNQTTTTDIKDDFADFVAFNGIKTKTNDKQELDFFTSKPTVSIRRENETKS
STTSQSNDEDFGDFFTKTTSPVKESKKVGSTWNGLEDKFGLDFANFNLKKGDSEKKKVSM
NEMKSKANITSNLF

> SSTP_0000262800

MSNLFSGLASITKTVSDALNNPEIKKFQDQVTGYVMNFTDAEIKVRNATNDEGWGPTNQQ
CDEIASMTFQYDLCTEASEMLFKRMMENNKGTWRRTYKSLRLVDHLLKHGSDRFIRYTRE
HSAQIRGLQNFHYIDEKGKDQGINIRVKAKAIIQLLQDENLLIEERKKAKTMNRDKYVGY
SKEDMIHMSGSSSMSGFGNDYYNKYSDTISNVSKSQSMNDDIHKEANLFNFPDDKDMDHT
ELGIPNTPNNDDDFGEFESPIKVAEVPKKVEVDLFGDITPIAPPPNDVISPAFSPKSVVS
PGSKKQTTTNDLINLMDDFNVTQPSPTQQTSVNVFSATRMDSDILSVPQKTEIPKNNVGN
ILDIDFNTTNHNTNVQDDFADFVAFGNTPVTNETQQIDFFTSMFTISPKESKEITNTSII
QKNEEDFGDFVSEKKPSVKESKKVGSTWNGLEDKFGLDFANFNLKKGELEKKKVSMNEMKSKGSIPTNLF

> SVE_0055100

MANLFSGLASITKTVSDALNNPEVKKFQDQVTGYVMNFSDAEIKVRNATNDEGWGPTNQQ
CDEIASMTFQYDLCTEASEMLFKRMMENSKGTWRRTYKSLRLVDHLLKHGSDRFIRYTRE
HSTQIRGLQHFHYIDEKGKDQGINIRVKAKAIIQLLQDDDLLMEERKKAKTMNRDKYVGY
SKEDMIHMSGSSSMSGFGNDHYNKYSDSFSNVTKSQSMNDGVKTEANLFNFPDDKDIDHT
ELGIPDTPTNDDDFGDFESPAKIPDVPSKNEIDLFGDITPIAPPPSGITSPAFSPKTKII
LESVKRPVVTNDLISLMDDLNVTNPPPQTSINVFSVTRMDDDILSLPQKCEPPKEYVNNL
LDFDINTPNQTTTTDIKDDFADFVAFSDTTTKTNDKQELDFFTSKPTVSITRENKIKLST
TSQSNNGDFGDFFSKTASPVKENKKVGSTWNGLEDTFGLDFANFNLKKGDSEKKKVSMNE
MKSKANITSNFI

C. elegans tsn-1

> SRAE_X000148500

MKVIGLQYLSSKIYKKIADINEKESNYDKEEIEVRNNLACLYRIIDQMGLSQSVFNHLTA
RISKYKNEILINPFGLLYHEITASSLIKVDEKGDILDKGSTKYGINNAGYILHSAIHEYN
NEIGCVIHLHVPDVVAVSSMTCGLLPICQESMIIGQVTYHDYEGIINSEKEKIKFVNDLG
NKKVMLLRNHGFVCCGETIMEALFLTYHLIIACETQVKIMSMANYDHSKFIIPSVDAQNV
TFQIASGGAGGVNFEGTKNNKCEKKWKRGELEWEAWIRILDDMNLETVKIILELKLKMAD
QSAKTKIAYVKQVLSGDSIILMGTGDNSTDMFVCLAYTKAPHLGKAPTESTSGTQDEPFA
WECREFVRRRLVGRRVTFIREYIGSNGREFGQIIIGTENLDKCENISESATAAGLMEIKP
VKQSNEFVEKLLALQEKAKSSKLGKWIDESEHHKHIRNIQWNIQNIQGFVQKYKGTSIKA
IIENVRDGSSFRAYISQESAYITFVLSGIKCPALVKKEQYAEEAKLFVEARLLNREIDLI
VEGVDNQRIVASIAHRAGNIAVLLLREGYAKCIDWSIGLVTGGAQAYRDAEKVAKERKIN
VWKDYEGGTISQSKGQTNGIVVEVGLGDNIMIENGSGNIVKYYFSSLRPPRMDTQKENNT
SSKAFRALYDIPCMYMAREYLRRKLIGKSVKIVVDYIQPKSEQFPEKVCGTVYLDKENIC
ELLVKKGFAKVIKHRRDDENRSSQYDTLLAAEKEAESAKVNLWADNANTMSMRVTELIGD
YSRAKQFLPSLQRTTKHSGIIEFVTSGSRYRVFLPKENLVISMALSGITCPRAGRPGGSD
NEPFGVEAINFAKKIALQHDVEVEIDAIDKTGGFIGQIFVLQKTGNINVAKSLIENGLAY
VHSAIDKSRFANEFYAAEESAKKKKLNIWENYVDENKVVEVAPKENDNSERKQIFKRVVI
SYVDRATFKIYLQFYDHEKSLVDLMERLQICAKNSVSQILPKRGELCMAKDSQYGKWTRA
RIDGIRDNKANVFYIDYGNSEILNYNIDNIKPISAEDKALQQIAREVRLAFVNPPPMDEF
GDLSFVALTDILYQEEYIYAALEYKVGKDDYVTLAIDDKKAGVKYDVGKKLVSDGYLVTV
DDRHEPKFKAVLEEYLNAEQKARREHLNIWRYGDFTGNEI

> SPAL_0001731600

MADQSAKTKIAYVKQVLSGDSIILMGTGDNNTDMFVCLAYAKAPHLGKAPTDSTPGTQDE
PFAWECREFVRRRLVGRRVTFIREYIGSNGREFGQIIIGTENLDHCENISESATAAGLME
VKPVKQSNEFVEKLLALQEKAKASKLGRWIDESEHHKHVRNIQWNIPNIQGFVQKYKGTT
VKAIIENVRDGSSFRAYIPQESAYITFVLSGIKCPALLKKEPFAEEAKLFVEARLLNREI
DLVIEGIDNSRIVASIAHRAGNIAVLLLSEGYAKCIDWSIGLVTGGAQAYRDAEKSAKEK
KRNVWKDYEGGAVPQSKGQTNGVVVEVGLGDNIVVENGAGSTVKYYFASIRPPRMDTQKE
TNSSSKAFRALYDIPCMYAAREFLRRKLIGKNVRVVVDYIQPKSEQFPEKVCGTVYLDKE
NICEILVKKGYAKVIKHRRDDENRCSQYDALLAAEKEAENLKINLWADNVNTMTMRVTEL
IGDYSRAKQFLPSLQRVTKHSGIVEFVTSGSRYRIFLTKENLVISLALSGITCPRAGRPG
GNDNEPYGVEATNFAKKFALQHDVEVEIESVDKTGGFIGQIYLVNKGGNINVAKSLVENG
LAYVHGTIDKTRFASEFYAAEDSAKKKKLNLWENYI

> SSTP_0000862900

MADQSAKTKIAYVKQVLSGDSIILMGTGDNSTDMFVCLAYTKAPHLGKAPTESTSGTQDE
PFAWECREFVRRRLVGSRVTFIREYIGSNGREFGQIIMGTENLDQCENISESATAAGLME
IKPVKQSNEFVEKLLALQEKAKSAKIGRWMDESEHHKHVRNIQWNIPNIQGFVQKYKGTS
LKAIIENVRDGSSFRAYIPQESAYITFVLSGIKCPALVKKEQYAEEAKLFVEARLLNREI
DLIIEGVDNQRIVASIAHRAGNIAVLLLREGYAKCIDWSIGLVTGGAQAYRDAEKIAKER
KINVWKDYEGGVISQSKGQTNGVVVEVGLGDNILIENGSGNIVKYYFSSLRPPRMDTQKE
TNNLSKTFRALYDIPCMYVAREYLRKKLIGKSVRIVVDYIQPKSDQFPEKVCGTVYLDKE
NICELLVKKGFAKVIKHRRDDENRSSQYDTLLAAEKEAESSKINLWADNANTMTMRVTEL
VGDYSRAKQFLPSLQRTTKHSGIIEFVTSGSRYRVFLPKENLVISMALSGITCPRAGRIG
GNDNEPFGVEAMNFAKKIGLQHDVEVEIDAIDKTGGFIGQIFVLRKGGNINLAKSLVENG
FAYVHGAIDKSRYASEFYAAEEIAKKKKLNIWENYVDENKVVEVTPKENDNSERKQIFKR

VVISYVDRLTFKIYLQFYDHEKSLVDLMEKLQISAKNSVSQILPKRGELCIAKDSQYGKW
TRARIDGIRDNKANVFYIDYGNSETLSYNTDYIKPISAEDRALQQIAKEVHLAFVNPPPL
EEFGDLSFVALTDILYQEEYIYAALEYKVGKEDYVTIAVDDKKANTKYDVGKKLVSEGYL
VTVDDRREPKFKAVIEEYLSAEQKARRDHLNIWRYGDFTGNEI

> SVE_0099900

MADQSAKTKIAYVKQVLSGDSIILMGTGDNNTDMFVCLAYAKAPHLGKAPTDSAPGTQDE
PFAWECREFVRRRLVGRRVTFIREYIGSNGREFGQIIIGTENLDQCENISESATAAGLME
VKPVKQSNEFVEKLLVLQEKAKASKLGRWIDENEHHKHVRNIQWNIPNIQGFVQKYKGTT
VKAIIENVRDGSSFRAYIPQESAYITFVLSGIKCPALLKKEPFAEEAKLFVEARLLNREI
DLVIEGIDNSRIVASIAHRAGNIAVLLLSEGYAKCIDWSIGLVTGGAQAYRDAEKSAKER
KRNVWKDYEGSAVPQSKGQTNGVVVEVGLGDNIVVENGSGSTVKYYFASIRPPRMDTQKE
TNSSSKAFRALYDIPCMYAAREFLRRKLIGKNVRVVVDYIQPKSEQFPEKVCGTVYLDKE
NICEILVKKGYAKVIKHRRDDENRCSQYDALLAAEKEAENLKVNLWADNVNTMTMRVTEL
IGDYSRAKQFLPSLQRVTKHSGIVEFVTSGSRYRIFLPKESLVISLALSGITCPRAGRPG
GSDNEPYGVEATNFAKKFVLQHDVEVEIESVDKTGGFIGQIYLVSKGGNLNVAKSLVENG
LAYVHGTIDKTRFAGEFYAAEESAKKKKLNLWENYVDETKVVEEVSKEDDNSERKQIFKR
IVVSNVDRQTFKIYIQFYDHGKSLVDLMEKLQVSAKNSVSQILPKRGELCMAKDSQYGKW
TRARIDGIRDNNAHVYYVDYGNSEVLKYDSNNIKPLAAEDRALQQVAREVRLAFVNPPPM
EEFGDLSYDALTDILYSEEYIYAALEYKVGKDDYVTIAVDDKKANSKYDVGKKLVAEGYL
VTADDRREPKFKAVIDEYVNAEQKARRDHLNIWRYGDFTGNEV

C. elegans ain-1

> SRAE_2000380100

MRNNYMYSNNDYSQNHIKNPNGQWTGPVPNVAGYMNPEFGHPMIPGPFMEAPPMMAEGYP
PNSKLNQYVSGGGTWDDNNTGKVDNDDGSTPTFTFNKGAANVPMHLSGTDGGFENEFKRT
SGWHNKNRNVQMSIPPPTDNWNVGNSALDHHGVQSNWPPRPMNVPMGGSHMNNVPIQGGW
DNMKRQQNTGNYRGNKNNYIDNNMKPNIHHAYMPRNDMNYPRNNMNSGFNNAPNMLWNQN
NNLNSQYINPRIPMGHHHEIQIQRQNTNQNMNPPHVNVGVNQLPVQGGNRNINNQHENRN
KHDSSSSMVLGNTSNHSNIQTPTETPSNVFSNTEDSYWRDPNGEMKKWQRDTGTAAWGDP
EDNNNSTIKRWMIPSIPDLLEGDHMLSGMEKENGVKIVVARGWGDENQSGMNSPQIKSNT
SVASPLYNKNDNRWSPDSSKGNRHGLYGSGDGSDWEADKNAHPFSGLTKGGNDIVSRDDH
IQNTQPSPELIKQGDPQYTKQLNTLLGYVQQAHFLENQLDNFRRANENSFAANDNPEIQN
EAKKNHNALIVEVARVKNEIVSLRDSLKNRHSGMPSPSNCELPPGSLLHSIKSEYNFPPS
NDDFLHDSLSTTNATFGMDSASAAVNRALANLDMSGNDNQTVSLLNSGVWNLG

> SRAE_X000088200

MNTNYMYQTYDGSQNQYQNNIDPRNRPNYVNIPNNNRNIQYQQTDINNPYNYGFITNPPP
INGSLMMNQCPPNLQQNNKDNVNMQFTINKGAANVPSHLSSPYRRQDNKNNSGYTNKNKN
SSSTKSPINDNWNLEYNMSQGGWNSHMNVSMGGINQSNNWEDINRLPPQNSNNYRNNNRN
NFHEVNIQSNTNSQYSQQRNDIRNIKFPTINQPPNNHNISSCISNNDMNSGQFNPQLNQY
GVPVSRSNTNFYNNSPVPINTVSKQNVGSKGNKNYGGRINYNYNNNQNTNRNIVPQNSVL
PGKVGIFNVSEDSYWKNPNEENLKKQRDNGTSIWGDPEKNNSLPIRRWGIPSIPDLIEGN
SSICDTKNTNVGSCKIIVATGWGDIEKKDDLKKNPKEFNKMSDESSLYNRDENHWSPGSG
KGNTFETFSNNQDHDWNLTGNTNIYPDIIRGFQGNNVSTPSDEYSLNSSSQELLKIAVSK
NLINKNLINKFYDPQSTVFMNNLLSLVSQAVMLDDKLGSTRRHDMDPNDFEQKQRYNSMI
VEIAKVKNEIETLSENVTNRAFGNNISANSNLPSNYLIANVHKDINFAAFTDEYLFENIS
STTNFHFDSATAAVSRSLANLDISGNGETPDHILHNDVWK

> SPAL_0000585900

MRNNYMYSNSDYSQNHMKNPNGQWNGPVPNVAGYMNPDFGHAMIPGPFMEAPPMMNEAYP
PNSKLNQYVSGGGAWDDTNTGKVDADDVNGPSFTFNKGAANVPMHLSGTDGGFENEFKRN
SGWHNKNRNVQMNIPPPTDNWNVGNSGIDHHNVQGNWPRPMNVPMGGNHMNNVPIQGGWD
NMKRQQNAGNYRGNKNSYIDNNMKQNMHHGYIPRNDINFPRNNMNSGFNNTPNMLWNQNN
NLNSQYINPRIPIGHHHEIPLPRQNTNQNINPPHVNVGGNQLPVQGGNRNISNQHENRNK
HDSTSSMVLGNTTNHSNIQTPTENPGNVFSNAEDSYWRDPNGEMKKWQRDTGTAAWGDPE

ENNNSTIKRWMIPSIPDLLEGDHMISGIEKENGVKIVIARGWGDENHSGMNSPQIKSNTS
VASPLYNKNENRWSPDSGKGNRLGIYGSGDGSDWEADKNAHPFSGLVKGANDMVSRDDHL
QNTPPSPDLIKLALSKKYIDNSMLNRQGDPQYTKQLNLLLGYVQQASFLENQLDNFRRAN
ENSFAANDNPEIQNEAKKNHNALIVEVARVKNEIVSLRDMLKNRLSGMPSPSNCELPPGS
LLHSIKSDYNFTTTNDDFLHDTLTTTNAGYGMDSASAAVNRALANLDISGSDNQSGNILASTVWNIN

> SPAL_0001296900

MNGNYMYQAYDGSQNQYQNTIDPAMRNRPNFMNIPNNNRNIQYQQSDMNNPYNYSFLNNP
PPMNGSMMINQCPQPLPPNTKDNSNMQFTINKGAANVPSHLSSPYRRQDNKNNSGYSSGG
KNNKNSPSSTNTAKSPLNDNWNMDYNIPQGNWNPHINIPIGGINQQSNWEDVSRSLPQNT
NNYRNNNRNNFHDMSIPQNTNTSYSQQRSDIRNVKFPNMNQPPNNQNIPVITGNDMNSGQ
YNPQLNQYGASLSRSNSNNYFNNSPVPMNTSGPKQGMGSKSTKNYGGRVNYNYNNNQNPN
RNIVPQNNLFPGKVGMFNVTEDSYWKNPNEENMRKQKDNGTSIWGNPDKQNTLPIKRWAM
ANIPDLVEGNDPSDTTSPPNPSCKVIVAVGWGDFDKKDEKKIQKDTCKVADDGNLYNRDE
NRWSPGSGKGSNFETFGSNHGSDWNLSGNPNNMYTDMIRGFQGNNMPNPSDGHSLSSTTQ
ELLKLAVSKNLINRNLLPKFYDPQSTVLLNNLLSLISQAVMLDDKLESIRRLEMGSTNDY
EMKQRQNALIVEIAKIKNEIIMVSENLNNRAFGNTLSVNGNLPPNHLVNNVHKDINLAAF
TDEYLFENISSTSNFHFDSATAAVSRSLANLDISGNGDETSDHLFHSDVWK

> SSTP_0000418700

MSNMRNNYMYSNSDYSQNHIKNPNGQWTGPVPNVAGYMNPEFGHPMIPGPFMEAPPMMAE
GYPPNSKLNQYVSGGGAWDDNSTGKVDTDDGSTPTFTFNKGAANVPMHLSGTDGGFENDF
KRTSGWHNKNRNVQMNIPPPNDNWNVGNSGLDHHGVQNNWPRPMNVPMGGSHMNNVPMQG
GWDNMKRQQNNGNYRGNKNNYIDNNMKQNMHHAYIPRNDMNYPRTNMNSGFNNAPNMLWN
QNNNLSSQYINPRIPMGHHHEIQIQRQNTNQNMNPPHVNVGVNQLPVQGGSRNINNQHEN
RNKHDSSSSMVLGNTSNHSNIQTPTETPSNVFSNTEDSYWRDPNGEMKKWQRDTGTAAWG
DPEDNNNSTIKRWMIPSIPDLLEGDHMISGIEKENGVKIVIARGWGDENQSGMNSPQIKS
NTSVASPLYNKNENRWSPDSSKGNRLGLYGSGDGSDWEADKNAHPFSGLVKGGNDMVSRD
DHIQNTQPSPELIKLALSKKYIDSSMLNRQADPQYTKQLNLLLGYVQQASFLENQLDNFR
RANENSFAANDNPEIQNEAKKNHNALIVEVARVKNEIVSLRDSLKNRHSSMPSPSNCELP
PGSLLHSIKTEYNFPPSNDDFLHDSLATSNATFGMDSASAAVNRALANLDMSGNDNQTVSLLNSGVWNIG

> SSTP_0000495400

MNTNYMYQTFDVTQNQYQNNIDPRNRSNFINIPNNNRNIQYQQSDMNNPYNYSYINNPPP
MNGSLMINQCPPHLQQNNKDNGNMQFTINKGAANVPSHLSSPYRRQDTKNNSGYTNKNKN
SSTTKSPINDSWNLDYNTSQGGWNSHMGVSMGGINQSNNWEDLNRLPPQNTNNYRNNNRN
NFHELNVQSNTNLPFPQQRNDIRNIKFPTINQPPNNQNISCISSNDINSGQFNPQLNQYG
VPVSRSNNNFYNNSPAPTTNATSKQNVGSKNNKNFGGRINYNYNNNQNSNRNIIPQNNTL
PGKVGMFNVTEDSFWKNPNEESIKKQKDNGTSIWGDPEKNNSLPIRRWGIPNIPDLVESN
TTIYDNKNTNNSSCKIIVATGWGDIEKKDNLKKNQKDLNKITDEGSLYNRDENHWSPGSG
KESTFEAFNNNPNSEWNLPGNTNLYSDMMRGFQGNNVSTPSDEYSLSSSSQELLKIAVSK
NIISKNILTKFYDPQSSVLLNSLLSLISQAVMLDDKLGSIRRNNMDQNDFEQKQRYNIMI
VEIAKIKNEIETLSENLTNRAFGNTISVNSNLPSNYLVTNVHKDINFAAFTDEYLFENIS
STTNFHFDSATAAVSRSLANLDISGNGETSDHLLHSDVWK

> SVE_0052100

MNGNYMYQAYDGSQNQYQNTIDPAMRNRSNFINVPNNNRNIQYQQSDMNNPYNYSFLNNP
PPMNGSMMINQCPQSLPPNTKDNSNMQFTINKGAANVPSHLSSPYRRQDNKNNSGYSSGG
KNNKNSSSSTNTAKSPLNDNWNMDFNNSQGNWNPHINIPIGGINQQSNWEDVSRSLPQNT
NNYRNNSRNNFHDMSIPQNTNTSYSQQRSDIRNVKFPNMNQPQSNQNIPVISGNDMNSGQ
YNPQLNQYGASLSRSNSNNYFNNSTVPMNTSAPKQGMGSKSTKSYGGRVNYNYNNNQNSN
RNIVTQNNLFPGKVGMFNVTEDSYWKNPNEENMRKQKDNGTSIWGNPDKQNTLPIKRWAM
ANIPDLVEGNDPSDTSSPPNPSCKVIVAVGWGDFDRKDEKKIPKDTCKVVDDGNLYNRDE
NRWSPGSGKGSNFETFGSSHGSDWNLSGNPNNIYTDMIRGFQGNNMPNPSDNHSLSSTTQ
ELLKLAVSKNLINRNLLPKFYDPQSSVLLNNLLSLISQAVMLDDKLESIRRLEMGPTNDY
EMKQRHNALIVEIAKIKNEIVMVSENLNNRTFGNTLSVNGNLPPNHLINNVHKDINLAAF
TDEYLFENISSTPNFHFDSATAAVSRSLANLDISGNGDETSDHLFHSDVWK

> SVE_1648100

MYSNSDYSQNHMKNPNGQWNGPVPNVAGYMNPDFGHAMIPGPFMEAPPMMNEAYPPNSKL
NQYVSGGGTWDDTNNGKVDTDDVNGPSFTFNKGAANVPMHLSGTDSGFENEFKRNSGWHN
KNRNIQMNIPPPTDNWNVGNSGIDHHNVQGNWPRPMNVPMGGNHMNNVPIQGGWDNMKRQ
QNAGNYRGNKNSYIDNNMKQNMHHGYIPRNDINFPRNNMNSGFNNTPNMLWNQNNNLNSQ
YINPRIPIGHHHEIPLPRQNTSQNINPPHVNVGGNQLPVQGGNRNINNQHESRNKHDSTS
SMVLGNTTNHSNIQTPTENPGNVFSNAEDSYWRDPNGEMKKWQRDTGTAAWGDPEENNNS
TIKRWMIPSIPDLLESDHMISGIEKENGVKIVIARGWGDENHSGMNSPQIKSNTSVASPL
YNKNENRWSPDSGKGNRLGIYGSGDGSDWEADKNAHPFSGLVKGANEMVSRDDHLQNTPP
SPDLIKLALSKKYIDNSMLNRQGDPQYTKQLNLLLGYVQQASFLENQLDNFRRANENSFA
ANDNPEIQNEAKKNHNALIVEVARVKNEIVSLRDMLKNRLSGMPSPSNCELPPGSLLHSI
KSDYNFTTANDDFLHDTLTTTNAGYGMDSASAAVNRALANLDISGSDNQSGSILASTVWNIN

C. elegans vig-1

> SRAE_X000171800

MEYKVKVSNKFHFCSDDEDVADPSELLARAIANKKKKKEEDALKKKVAEQKAKETPVTDV
VKQPTEQRKPVREGEKKKQSPRGRGGFRGERRPKEEGRPKEEGRSKEEVRPKRTEEKGSL
NVEEGVDDDFEISRRPPRNAGKKPNFKKDNNERKPRASGSTKTGVRSMPKRDGFGKGNWG
TQKDELTGETEPLNQTAVDESENVAPGIKNNNTAEVPEEEKTLTLEEYKAQLAAAKKDET
PFNIRQISEQEFKKLNLIPIPKKKVEQVTEEVEIIRREPRKQVINLDLKFVDTNKKSRRE
PVNRRGNNHRRGKQSQGFVYKENAFPALGSA

> SPAL_0000359300

MEYKVKVSNKFHFCSDDEDVADPSELLARAIANKKKKKEEDAMKKKVADEKAKETPVGVV
AKPPTEQRKPTRDGEKRNQSSRGRGGFRGGERRPREEGRPKRNEEKAPVTTEEGVDDDFE
INRRPPRNNGKKPNRKMENGERKPRASGSNKTGVRAMPKREGFGKGNWGTQKDELTGETE
PLNQQPTDESENVAPVAKNNDTAEVPEEEKTLTLEEYKAQLAAAKKDETPFNIRQISEQE
FKKLNLIPIQKKKVEQTTEEVEIIRREPRKQVINLDLKFVDTNKKTRRDNRDNNVNRRGN
NRRGKQPQGFVYKENAFPALGSA

> SSTP_0000432100

MEYKVKVSNKFHFCSDDEDVADPSELLARAIANKKKKKEEDALKKKAAEQKAKETPVADV
AKQQTEQRKVTREGEKKKQSPRGRGGFRSERRPKEEGRPREEGRPKRVEEKESVAVEEGV
DDDFEINRRPVRNGGKKPNFKKDNNERKPRASGSNKTGVRSIPKRDGFGKGNWGTQKDEL
AGETEPLNQTAVDESENVAPGVKNNNTAEVPEEEKTLTLEEYKAQLAAAKKDETPFNIRQ
ISEHEFKKLNLIPIPKKKVEQVTEEVEIIRREPRKQIINLDLKFVDTNKKSRRDNVPRRG
NNRRGKQSQGFVYKENAFPALGSA

> SVE_0663200

MEYKVKVSNKFHFCSDDEDVADPSELLARAIANKKKKKEEDAMKKKVADEKAKETPVGVV
AKPPNEQRKPTRDGEKRNQSTRGRGGFRGGERRPREEGRPKRTEEKAPVTTEEGVDDDFE
INRRPPRNNGKKPNRKMENGERKPRASGSNKTGVRAMPKREGFGKGNWGTQKDELAGETE
PLNQQPTDESENVAPVAKNNDTAEIPEEEKTLTLEEYKAQLAAAKKDETPFNIRQISEQE
FKKLNLIPIQKKKVEQTTEEVEIIRREPRKQVINLDLKFVDTNKKTRRDNRDNNVNRRGN
NRRGKQPQGFVYKENAFPALGSA

C. elegans ain-2

> SRAE_2000380100

MRNNYMYSNNDYSQNHIKNPNGQWTGPVPNVAGYMNPEFGHPMIPGPFMEAPPMMAEGYP
PNSKLNQYVSGGGTWDDNNTGKVDNDDGSTPTFTFNKGAANVPMHLSGTDGGFENEFKRT
SGWHNKNRNVQMSIPPPTDNWNVGNSALDHHGVQSNWPPRPMNVPMGGSHMNNVPIQGGW

DNMKRQQNTGNYRGNKNNYIDNNMKPNIHHAYMPRNDMNYPRNNMNSGFNNAPNMLWNQN
NNLNSQYINPRIPMGHHHEIQIQRQNTNQNMNPPHVNVGVNQLPVQGGNRNINNQHENRN
KHDSSSSMVLGNTSNHSNIQTPTETPSNVFSNTEDSYWRDPNGEMKKWQRDTGTAAWGDP
EDNNNSTIKRWMIPSIPDLLEGDHMLSGMEKENGVKIVVARGWGDENQSGMNSPQIKSNT
SVASPLYNKNDNRWSPDSSKGNRHGLYGSGDGSDWEADKNAHPFSGLTKGGNDIVSRDDH
IQNTQPSPELIKQGDPQYTKQLNTLLGYVQQAHFLENQLDNFRRANENSFAANDNPEIQN
EAKKNHNALIVEVARVKNEIVSLRDSLKNRHSGMPSPSNCELPPGSLLHSIKSEYNFPPS
NDDFLHDSLSTTNATFGMDSASAAVNRALANLDMSGNDNQTVSLLNSGVWNLG

> SRAE_X000088200

MNTNYMYQTYDGSQNQYQNNIDPRNRPNYVNIPNNNRNIQYQQTDINNPYNYGFITNPPP
INGSLMMNQCPPNLQQNNKDNVNMQFTINKGAANVPSHLSSPYRRQDNKNNSGYTNKNKN
SSSTKSPINDNWNLEYNMSQGGWNSHMNVSMGGINQSNNWEDINRLPPQNSNNYRNNNRN
NFHEVNIQSNTNSQYSQQRNDIRNIKFPTINQPPNNHNISSCISNNDMNSGQFNPQLNQY
GVPVSRSNTNFYNNSPVPINTVSKQNVGSKGNKNYGGRINYNYNNNQNTNRNIVPQNSVL
PGKVGIFNVSEDSYWKNPNEENLKKQRDNGTSIWGDPEKNNSLPIRRWGIPSIPDLIEGN
SSICDTKNTNVGSCKIIVATGWGDIEKKDDLKKNPKEFNKMSDESSLYNRDENHWSPGSG
KGNTFETFSNNQDHDWNLTGNTNIYPDIIRGFQGNNVSTPSDEYSLNSSSQELLKIAVSK
NLINKNLINKFYDPQSTVFMNNLLSLVSQAVMLDDKLGSTRRHDMDPNDFEQKQRYNSMI
VEIAKVKNEIETLSENVTNRAFGNNISANSNLPSNYLIANVHKDINFAAFTDEYLFENIS
STTNFHFDSATAAVSRSLANLDISGNGETPDHILHNDVWK

> SPAL_0000585900

MRNNYMYSNSDYSQNHMKNPNGQWNGPVPNVAGYMNPDFGHAMIPGPFMEAPPMMNEAYP
PNSKLNQYVSGGGAWDDTNTGKVDADDVNGPSFTFNKGAANVPMHLSGTDGGFENEFKRN
SGWHNKNRNVQMNIPPPTDNWNVGNSGIDHHNVQGNWPRPMNVPMGGNHMNNVPIQGGWD
NMKRQQNAGNYRGNKNSYIDNNMKQNMHHGYIPRNDINFPRNNMNSGFNNTPNMLWNQNN
NLNSQYINPRIPIGHHHEIPLPRQNTNQNINPPHVNVGGNQLPVQGGNRNISNQHENRNK
HDSTSSMVLGNTTNHSNIQTPTENPGNVFSNAEDSYWRDPNGEMKKWQRDTGTAAWGDPE
ENNNSTIKRWMIPSIPDLLEGDHMISGIEKENGVKIVIARGWGDENHSGMNSPQIKSNTS
VASPLYNKNENRWSPDSGKGNRLGIYGSGDGSDWEADKNAHPFSGLVKGANDMVSRDDHL
QNTPPSPDLIKLALSKKYIDNSMLNRQGDPQYTKQLNLLLGYVQQASFLENQLDNFRRAN
ENSFAANDNPEIQNEAKKNHNALIVEVARVKNEIVSLRDMLKNRLSGMPSPSNCELPPGS
LLHSIKSDYNFTTTNDDFLHDTLTTTNAGYGMDSASAAVNRALANLDISGSDNQSGNILASTVWNIN

> SPAL_0001296900

MNGNYMYQAYDGSQNQYQNTIDPAMRNRPNFMNIPNNNRNIQYQQSDMNNPYNYSFLNNP
PPMNGSMMINQCPQPLPPNTKDNSNMQFTINKGAANVPSHLSSPYRRQDNKNNSGYSSGG
KNNKNSPSSTNTAKSPLNDNWNMDYNIPQGNWNPHINIPIGGINQQSNWEDVSRSLPQNT
NNYRNNNRNNFHDMSIPQNTNTSYSQQRSDIRNVKFPNMNQPPNNQNIPVITGNDMNSGQ
YNPQLNQYGASLSRSNSNNYFNNSPVPMNTSGPKQGMGSKSTKNYGGRVNYNYNNNQNPN
RNIVPQNNLFPGKVGMFNVTEDSYWKNPNEENMRKQKDNGTSIWGNPDKQNTLPIKRWAM
ANIPDLVEGNDPSDTTSPPNPSCKVIVAVGWGDFDKKDEKKIQKDTCKVADDGNLYNRDE
NRWSPGSGKGSNFETFGSNHGSDWNLSGNPNNMYTDMIRGFQGNNMPNPSDGHSLSSTTQ
ELLKLAVSKNLINRNLLPKFYDPQSTVLLNNLLSLISQAVMLDDKLESIRRLEMGSTNDY
EMKQRQNALIVEIAKIKNEIIMVSENLNNRAFGNTLSVNGNLPPNHLVNNVHKDINLAAF
TDEYLFENISSTSNFHFDSATAAVSRSLANLDISGNGDETSDHLFHSDVWK

> SSTP_0000418700

MSNMRNNYMYSNSDYSQNHIKNPNGQWTGPVPNVAGYMNPEFGHPMIPGPFMEAPPMMAE
GYPPNSKLNQYVSGGGAWDDNSTGKVDTDDGSTPTFTFNKGAANVPMHLSGTDGGFENDF
KRTSGWHNKNRNVQMNIPPPNDNWNVGNSGLDHHGVQNNWPRPMNVPMGGSHMNNVPMQG
GWDNMKRQQNNGNYRGNKNNYIDNNMKQNMHHAYIPRNDMNYPRTNMNSGFNNAPNMLWN
QNNNLSSQYINPRIPMGHHHEIQIQRQNTNQNMNPPHVNVGVNQLPVQGGSRNINNQHEN
RNKHDSSSSMVLGNTSNHSNIQTPTETPSNVFSNTEDSYWRDPNGEMKKWQRDTGTAAWG
DPEDNNNSTIKRWMIPSIPDLLEGDHMISGIEKENGVKIVIARGWGDENQSGMNSPQIKS
NTSVASPLYNKNENRWSPDSSKGNRLGLYGSGDGSDWEADKNAHPFSGLVKGGNDMVSRD

DHIQNTQPSPELIKLALSKKYIDSSMLNRQADPQYTKQLNLLLGYVQQASFLENQLDNFR
RANENSFAANDNPEIQNEAKKNHNALIVEVARVKNEIVSLRDSLKNRHSSMPSPSNCELP
PGSLLHSIKTEYNFPPSNDDFLHDSLATSNATFGMDSASAAVNRALANLDMSGNDNQTVSLLNSGVWNIG

> SSTP_0000495400

MNTNYMYQTFDVTQNQYQNNIDPRNRSNFINIPNNNRNIQYQQSDMNNPYNYSYINNPPP
MNGSLMINQCPPHLQQNNKDNGNMQFTINKGAANVPSHLSSPYRRQDTKNNSGYTNKNKN
SSTTKSPINDSWNLDYNTSQGGWNSHMGVSMGGINQSNNWEDLNRLPPQNTNNYRNNNRN
NFHELNVQSNTNLPFPQQRNDIRNIKFPTINQPPNNQNISCISSNDINSGQFNPQLNQYG
VPVSRSNNNFYNNSPAPTTNATSKQNVGSKNNKNFGGRINYNYNNNQNSNRNIIPQNNTL
PGKVGMFNVTEDSFWKNPNEESIKKQKDNGTSIWGDPEKNNSLPIRRWGIPNIPDLVESN
TTIYDNKNTNNSSCKIIVATGWGDIEKKDNLKKNQKDLNKITDEGSLYNRDENHWSPGSG
KESTFEAFNNNPNSEWNLPGNTNLYSDMMRGFQGNNVSTPSDEYSLSSSSQELLKIAVSK
NIISKNILTKFYDPQSSVLLNSLLSLISQAVMLDDKLGSIRRNNMDQNDFEQKQRYNIMI
VEIAKIKNEIETLSENLTNRAFGNTISVNSNLPSNYLVTNVHKDINFAAFTDEYLFENIS
STTNFHFDSATAAVSRSLANLDISGNGETSDHLLHSDVWK

> SVE_0052100

MNGNYMYQAYDGSQNQYQNTIDPAMRNRSNFINVPNNNRNIQYQQSDMNNPYNYSFLNNP
PPMNGSMMINQCPQSLPPNTKDNSNMQFTINKGAANVPSHLSSPYRRQDNKNNSGYSSGG
KNNKNSSSSTNTAKSPLNDNWNMDFNNSQGNWNPHINIPIGGINQQSNWEDVSRSLPQNT
NNYRNNSRNNFHDMSIPQNTNTSYSQQRSDIRNVKFPNMNQPQSNQNIPVISGNDMNSGQ
YNPQLNQYGASLSRSNSNNYFNNSTVPMNTSAPKQGMGSKSTKSYGGRVNYNYNNNQNSN
RNIVTQNNLFPGKVGMFNVTEDSYWKNPNEENMRKQKDNGTSIWGNPDKQNTLPIKRWAM
ANIPDLVEGNDPSDTSSPPNPSCKVIVAVGWGDFDRKDEKKIPKDTCKVVDDGNLYNRDE
NRWSPGSGKGSNFETFGSSHGSDWNLSGNPNNIYTDMIRGFQGNNMPNPSDNHSLSSTTQ
ELLKLAVSKNLINRNLLPKFYDPQSSVLLNNLLSLISQAVMLDDKLESIRRLEMGPTNDY
EMKQRHNALIVEIAKIKNEIVMVSENLNNRTFGNTLSVNGNLPPNHLINNVHKDINLAAF
TDEYLFENISSTPNFHFDSATAAVSRSLANLDISGNGDETSDHLFHSDVWK

> SVE_1648100

MYSNSDYSQNHMKNPNGQWNGPVPNVAGYMNPDFGHAMIPGPFMEAPPMMNEAYPPNSKL
NQYVSGGGTWDDTNNGKVDTDDVNGPSFTFNKGAANVPMHLSGTDSGFENEFKRNSGWHN
KNRNIQMNIPPPTDNWNVGNSGIDHHNVQGNWPRPMNVPMGGNHMNNVPIQGGWDNMKRQ
QNAGNYRGNKNSYIDNNMKQNMHHGYIPRNDINFPRNNMNSGFNNTPNMLWNQNNNLNSQ
YINPRIPIGHHHEIPLPRQNTSQNINPPHVNVGGNQLPVQGGNRNINNQHESRNKHDSTS
SMVLGNTTNHSNIQTPTENPGNVFSNAEDSYWRDPNGEMKKWQRDTGTAAWGDPEENNNS
TIKRWMIPSIPDLLESDHMISGIEKENGVKIVIARGWGDENHSGMNSPQIKSNTSVASPL
YNKNENRWSPDSGKGNRLGIYGSGDGSDWEADKNAHPFSGLVKGANEMVSRDDHLQNTPP
SPDLIKLALSKKYIDNSMLNRQGDPQYTKQLNLLLGYVQQASFLENQLDNFRRANENSFA
ANDNPEIQNEAKKNHNALIVEVARVKNEIVSLRDMLKNRLSGMPSPSNCELPPGSLLHSI
KSDYNFTTANDDFLHDTLTTTNAGYGMDSASAAVNRALANLDISGSDNQSGSILASTVWNIN

C. elegans eri-1

> SRAE_2000336900

MVNSSKFFHIKSCKKECVQYFNYFLVIDFEATCEINSGPDYYHEIIEFPCLLVDGKTKQI
LSTFHSYVRPKKNPNITAFCTELTGISQNVIDEAPPLDKVWDMFLGWLEEHGVDYDKVST
EFVIITDGDADIGRFLIPNLHDSNCYIPKSFYYYVDLRCSMLKILPKVSYLQIHKMTLKA
ILMKFSLEFEGKEHSGMDDARNILALLKICIENNVDILPNSFVEIKKEYDEKTMEEYKYY
QCALLKKEMCFTIFGLTR

> SPAL_0000221600

MVNSSKLFQIKSCKKNCIQYFNYFLVIDFEATCEINSGSDYYHEIIEFPCILVDGKSKQI
VSTFHSYVKPRKNPNITAFCTELTGISQNIIDEAPPLDKVWRMFLNWLQENNVDYEKVST

EFVVITDGDADIGRFLIPNLHDLNCYIPKSFYYYVDLRCSMLKILPKVSYLQIHKMSLKA
ILTKFSLSFEGKEHSGMDDARNILALLKVCIQNNVDILPNSFVEIKKEYDKIKMEEYKYY
QCALLKKDICFTIFGLTR

> SSTP_0000128100

MVNSSKFFHVKSCRKDCVQYFDNFLVIDFEATCETNSGPDYYHEIIEFPCILVDGKTKEI
ISTFHSFVKPKKNPNITAFCTKLTGISQNVIDKAPPLDKVWNMFLDWLQEHNVDYNKTSK
EFVIITDGDTDIGRFLIPNLHDLNCYIPESFYYYVDLRCSLLKILPKISYLQIHKMSLKA
ILTRFSLSFEGKEHSGMDDAKNILALLKICIKNNVDILPNSFVEIKKEYDKETMEEYKYY
QCALLKKEICFTIFGLTR

> SVE_173900

MVNSSKLFQIKSCKKNCIQYFNYFLVIDFEATCENNSGPDYYHEIIEFPCILVDGKSKQI
VSTFHSYVKPRKNPNITAFCTELTGISQNIIDEAPPLDKVWCMFLNWLQENNLDYEKVST
EFVVITDGDADIGRFLIPNLHDLNCYIPESFYYYVDLRCSMLKILPKVSYLQIHKMSLKA
ILTKFSLSFEGKEHSGMDDAKNILALLKVCIQNNVDILPNSFVEIKKEYDTTKMEEYKYY
QCALLKKDICFTIFGLTR

C. elegans xrn-2

> SRAE_2000328200

MGVPAFFRWLSRKYPSIVSDVIEEECQIVDGVRIPIDCTKPNLNFQEFDNLYLDMNGIIH
PCAHPEDRPPPETEDDIFVLIFEYIDRLMAIIRPRKLLYMAIDGVAPRAKMNQQRSRRFR
TAKESADKKKEIQEVKDKLMSQGIPVIQRKKETNWFDSNCITPGTPFMERLAKALRYYIA
LKIKTDPAWANILVILSDASVPGEGEHKIMDFIRKQRASSGHDPNTAHCLCGADADLIML
GLATHEANFTIIREEFIPRQMSPCELCNLVGHTLQECDGGIIESSIVKTVPVKRRTNFIF
IRLPVLREYLEKELIMYEINFKFDLERAIDDWVFLCFLVGNDFLPHLPSLEIRENAICRL
VRLYKQMASKCGGYLTDNGEVNMQRISMILAELGDVEDDIFKRRQENEQKFKDKKKSMKC
RNTNYNKASSFIAPNVDGGLITPVPIENANLNNFRAETKIKAYMARVEQEDCLVAERRLL
SILKPDKVAELPPLNPQVFQVVGPNGKTIGEESDSEPEDNVKFYEPGWKNRYYEEKFGAS
FSNVSEEFRRIVAEAYMEGLCWVLKYYYSGCPSWDWFYPYHYAPFASDFDKIEHYKPNFP
RNTKPFNPLEQLMGVFPAASSQHVPECWRKLMTDIDSEISDFYPTDFAIDLNGKKFSWMG
VALLPFVDQERLLACLEKYSDGLTEDEKKRNIRGENHIYVSKVHSAFEKIREIYETNQDV
TWISLNPTSNNGVTLLISRMEDCINLDEPYYSPVKAMICKDFTNSCAMALVRDPFFVDQH
HYPVHRLEGVIEPERIVSTFNGRRPYNNKKEYHQYNNRKEQHNVKKDYQQQSNNGKSYHK
PTTKNEYFRTNNRKDVHDFRNSRDVHHNSNTNNQFSNNVVNGNFRNNGSGNNFNNRNPRV
NHADNNSRAGNSQEKRYQPNKGTFQKRNFGLKPKQNFIHQPENQ

> SRAE_2000328700

MGVPAFFRWLSRKYPTIVGDVVEEECQIVDGVRIPIDCTKPNLNFQEFDNLYLDMNGIIH
PCSHPEDRPAPETEDDIFVLIFEYIDRLMAIIRPRKLLYMAIDGVAPRAKMNQQRSRRFR
TAKESADKKKEIQAVKDKLTAQGIPVIQRKKEANWFDGNCITPGTPFMERLAKALRYYIA
YKIKTDPSWAKIHIILSDASVPGEGEHKIMDFIRKQRASPGHDPNIAHCLCGADADLIML
GLATHEANFTIIREEFIPRQMSPCELCNLMGHTLQECDGGIIESSIIKTEPVKRKTNFIF
IRLPILREYLEKELIMYEINFKFDLERAIDDWVFLCFLVGNDFLPHLPSLEIRENAICRL
IKLYKQMASECGGYLTDNGEVNMQRMSMILAELGDAEDEIFKRRQDTEQRFKERNKAMKR
KHSSINKASSFIAPSVDGGLITPVPIKNAHLNNFGSQTKVKAYMARVEQEDCIVAERRLL
SILKPQKSDDVPPLIPQGMVSNNTVVVEESDSEPEDNVKFYEPGWKDRYYEEKFGVSFSD
VSLEFRRSVAEAYMEGLCWVLKYYYTGCPSWDWFYPYHYAPFASDFDKIEYYKPNFPKNT
KPFNPLEQLMGVFPAASSQHVPECWRKLMTDIDSEIYDFYPTDFAIDLNGKKHAWMGVAL
LPFVDQERLLACLEKYSDGLTEDEKKRNIRGENHIYVSKYHQAFKEIRGIYETNQDVTWI
SLNPASNNGVSMLISRMVDCVNPDEPYYSPIKTMIYKDFTNSSAMALVRDPFFVDQHYYP
VHRLEGVIEPERVLPLTNFYNSDYKRDYRKNNGSRDDSNFRNNYRNDYNYKKGYRGNNYV
NRGFRNDYSNRPIRRGMR

> SPAL_0001232100

```
MGVPAFFRWLSRKYPLIVSDVVEEESLGADGVRKPVDCTQPNPNFQEFDNLYLDMNGIIH
PCSHPEDRPAPETEDDIFVLIFEYIDRLMAIIRPRKLLYMAIDGVAPRAKMNQQRSRRFR
AAKESADKKKEVQAVKDKLMAQDIPIIQRKKEANWFDGNCITPGTPFMDRLTKALRYYIA
YKIKSDPSWANIHVILSDASVPGEGEHKIMDFIRKQRASSGHDPNTAHCLCGADADLIML
GLATHEANFTIIREEFVPRQMSPCELCNYMGHTLQECDGGIIESSIIKMEQPKRKTNFIF
IRLPILREYLEKELTMYDINFKFDLERAIDDWVFLCFLVGNDFLPHLPSLEIRENAICRL
VKLYKQMASECGGYLTDNGEVNMQRISMILAELGDSEDEIFKRRQENEQKFKERNKAMKR
KHPSSNKASSFIAPNIDGGLITPVPIASAHMNNFGGETKIKAYMARVQQEDCITAERRLL
SILKPEKSVNVPSVSSQSFQNVRQNGKVVDGGSDSEPEDNVKFYEPGWKDRYYEEKFGVSFSDVSLEFRRN
```

> SSTP_0000047400

```
MGVPAFFRWLSRKYPTIVGDVVEEECPIIDGVRIPIDCTKPNLNFQEFDNLYLDMNGIIH
PCSHPEDRPAPETEDDIFVLIFEYIDRLMAIIRPRKLLYMAIDGVAPRAKMNQQRSRRFR
SAKESADKKKEIQAVKDKLMAQGIPVIQRKKEVNWFDGNCITPGTPFMERLAKALKYYIA
FKIKNDPAWSKIHVILSDASVPGEGEHKIMDFIRKQRASSGHDPNTAHCLCGADADLIML
GLATHEANFTIIREEFIPRQMSPCELCNLMGHTLQECDGGVIESSIVKVDPVKRKTNFIF
IRLPILREYLEKELFMNDINFKFDLERAIDDWVFLCFLVGNDFLPHLPSLEIRENAICRL
VRLYKQMASECGGYLTDNGEVNMQRMSMILAELGDAEDEIFKRRQETEQRFKERNKAMKR
KHADISKASSLIAPNVYGGLITPVPIESAHLNNFGSETRKRAYMARVGQEDCNAGGKKLL
KILKPEKATDSSPLNSQVFQAAGPNGKVIDEESDSEPEDNVKFYEPGWKDRYYEEKFGVS
FSDVSLEFRRNVAKAYMEGLCWVLKYYYSGCPSWDWFYPYHYAPFASDFDKIEYYKPNFP
KNTKPFNPLEQLMGVFPAASSQHVPECWRKLMSDIDSEIYDFYPTDFAIDLNGKRHAWMG
VALLPFVDQNRLLACLEKYSDGLTEEEKKRNVRGENYLYVSKYHQAFKEIRAIYENNQDV
TWMSLNPASNNGVTMLISRMVDCINPDEPYYSPIKTMIYSDFTNSSAMALVRDPFFVDQH
YYPVHRLNGVIEPERILPSTNYCGSEYKREYRRNNVSRDDPNFKNNYRNDYNYKKGYRGN
NYINRGFRNEYNNKPIRRGMR
```

> SSTP_0000048000

```
MGVPAFFRWLSRKYPTIVGDVVEEECPIIDGVRIPIDCTKPNLNFQEFDNLYLDMNGIIH
PCAHPEDRPAPETEDDIFVLIFEYIDRLMAIIRPRKLLYMAIDGVAPRAKMNQQRSRRFR
SAKESADKKKEIQAVKDKLMAQGIPVIQRKKEVNWFDGNCITPGTPFMERLAKALKYYIA
FKIKNDPAWSKIHVILSDASVPGEGEHKIMDFIRKQRASSGHDPNTAHCLCGADADLIML
GLATHESNFTIIREEFIPRQMSPCELCNLMGHTLQECDGGVIESSIVKVDPVKRRTNFIF
IRLPILREYLERELFMNDINFKFDLERAIDDWVFLCFLVGNDFLPHLPSLEIRENAICRL
VRLYKQMASECGGYLTDNGEVNMQRISMILAELGGAEDEIFKRRQETEKRFKEKNRSVKR
KHANFNKTFSLITPKVDGDIVNQISIENVHLNNFESETKIKAYMARVEQEDCKTAEIRLL
SILKPEQVADMLPLNPQAFQVVGPNGKVIDEESDSEPEDNVKFYEPGWKDRYYEEKFSVS
FSDVSLEFRRNVARAYMEGLCWVLKYYYSGCPSWDWFYPYHYAPFASDFDKIEYYKPNFP
KNTKPFNPLEQLMGVFPAASSQHVPECWRKLMIDSNSEIYDFYPTDFAVDLNGKKHAWMG
VALLPFVDQDRLLACLEKYSDGLTEEEKRRNVRGENHLYVSKHHQSFKKIREIYENNQDV
TWISLNPASNNGITLLISRMVDCINPDEPYYSPVKAMICKDFTNSCAMALVRDPFFVDQH
CYPVHRLDGVIEPERHLPIYNGQKQKSRKDYRQYNNKKEQHNFRKKYQQPNDGKNYYKHN
NKNEHSCANNRKDLRDFKNKNDKHYQMNTNSQFLKKLFNNSFNNTHPGNNLNNHKSKVSH
INNYPRVDNYQEKKYPSTKVTFRRRNCESKPKQNFFHQPDIQ
```

> SVE_1730200

```
MGVPAFFRWLSRKYPLIVSDVIEEESLSADGVRKPIDCTKPNPNFQEFDNLYLDMNGIIH
PCSHPEDRPAPETEDDIFVLIFEYIDRLMAIIRPRKLLYMAIDGVAPRAKMNQQRSRRFR
AAKESADKKKEIQAVKDKLMAQDIPVIQRKREANWFDGNCITPGTPFMDRLTKALRYYIA
YKIKSDPSWANIHVILSDASVPGEGEHKIMDFIRKQRASSGHDPNTAHCLCGADADLIML
GLATHEANFTIIREEFVPRQMSPCELCNFMGHTLQECDGGIMETSLTRIEQPKRKTNFIF
IRLPILREYLEKELAMYDINFKFDLERAIDDWVFLCFLVGNDFLPHLPSLEIRENAICRL
VRLYKQMASECGGYLTDNGEVNMQRISMILAELGDSEDEIFKRRQENEQKFKERNKAMKR
RHINSNKASAFVAPNVHGGLITPVPVENAHLNNFGGETKIKAYMARVQQEDCITAERRLL
SILKPEKSLDLLSLNLQGFRIVGQNGKVLDEGSDSEPEDNVKFYEPGWKDRYYEEKFGVS
FSDVSLEFRRNVAEAYMEGMCWVLKYYYSGCPSWDWFYPYHYAPFASDFDQIEYYKPNFP
KNTKPFNPLEQLMGVFPAASSQHVPECWRKLMTDVNSEIYDFYPTDFAIDLNGKKQAWMG
```

VALLPFVDQDRLLDCLKKHSDGLTDEEKRRNIRGENHVYVSNQHPAYKKIREIYENIQDV
TWISLNPASNNGVILLISRMEDCINPDEPYYSPVKAMVCKDFTNSCAMALVRDPFFMDQH
YYPVHRLAGVIEPEKILPICSNDRHMNKKHYQQNNFRKNYNQTNSRRDFTQTDFGNNHNH
SNSKKDFHDFRNTKSNYYNGNKNRNNYSNNKMVNGDSKDKGNGNNHAFKVKYNDHHHHAS
NQTQGGRRHPTRNRFQERNAKPKYDRDITP

> SVE_1730800

MGVPAFFRWLSRKYPSIVGDVVEEECLTADGTRLPVDCTKPNPNFQEFDNLYLDMNGIIH
PCSHPEDRPAPETEDDIFVLIFEYIDRLMAIIRPRKLLYMAIDGVAPRAKMNQQRSRRFR
AAKESADKKKEIQAVKDKLMAQDIPVIQRKKETNWFDGNCITPGTPFMDRLTKALRYYIA
YKIKSDPSWANIHVILSDASVPGEGEHKIMDFIRKQRASSGHDPNTAHCLCGADADLIML
GLATHEANFTIIREEFVPRQMSPCELCNFMGHTLQECDGGIMETSLTRIEQPKRKTNFIF
IRLPILREYLEKELAMYDINFKFDLERAIDDWVFLCFLVGNDFLPHLPSLEIRENAICRL
VRLYKQMASECGGYLTDNGEVNMQRISMILAELGDSEDEIFKRRQENEQKFKERNKAMKR
KHPSSNKASSFIAPNIDGGLITPVPIASAHMNNFGGETKIKAYMARVQQEDCITAERRLL
SILKPEKSVNSPSVSSQSFQNVGQNGKVLDEGSDSEPEDNVKFYEPGWKDRYYEEKFGVS
FSDVSLEFRRNVAESYMEGMCWVLKYYYSGCPSWDWFYPYHYAPFASDFDKIEYYKPNFP
KNTKPFNPLEQLMGVFPAASSQHVPECWRRLMVDIESEIYDFYPTDFAIDLNGKKQAWMG
VALLPFVDQDRLLDCLKKHSDGLTDEEKRRNIRGENHVYVSKQHPAYRMIREIYESNQDV
TWISLSPTSNNGVTMLISRMADCVNPDEPYYSPIKAMVCRDFTNSCAMALVRDPFFMDQH
YYPVHRLAGVIEPERFLPLPNSYGPEYGREYRKGIRTRGDYNFKNNYRNDFNYRGGYQDS
NNANRGFRRNYNNRTFRHDTR

C. elegans xrn-1

> SRAE_2000328200

MGVPAFFRWLSRKYPSIVSDVIEEECQIVDGVRIPIDCTKPNLNFQEFDNLYLDMNGIIH
PCAHPEDRPPPETEDDIFVLIFEYIDRLMAIIRPRKLLYMAIDGVAPRAKMNQQRSRRFR
TAKESADKKKEIQEVKDKLMSQGIPVIQRKKETNWFDSNCITPGTPFMERLAKALRYYIA
LKIKTDPAWANILVILSDASVPGEGEHKIMDFIRKQRASSGHDPNTAHCLCGADADLIML
GLATHEANFTIIREEFIPRQMSPCELCNLVGHTLQECDGGIIESSIVKTVPVKRRTNFIF
IRLPVLREYLEKELIMYEINFKFDLERAIDDWVFLCFLVGNDFLPHLPSLEIRENAICRL
VRLYKQMASKCGGYLTDNGEVNMQRISMILAELGDVEDDIFKRRQENEQKFKDKKKSMKC
RNTNYNKASSFIAPNVDGGLITPVPIENANLNNFRAETKIKAYMARVEQEDCLVAERRLL
SILKPDKVAELPPLNPQVFQVVGPNGKTIGEESDSEPEDNVKFYEPGWKNRYYEEKFGAS
FSNVSEEFRRIVAEAYMEGLCWVLKYYYSGCPSWDWFYPYHYAPFASDFDKIEHYKPNFP
RNTKPFNPLEQLMGVFPAASSQHVPECWRKLMTDIDSEISDFYPTDFAIDLNGKKFSWMG
VALLPFVDQERLLACLEKYSDGLTEDEKKRNIRGENHIYVSKVHSAFEKIREIYETNQDV
TWISLNPTSNNGVTLLISRMEDCINLDEPYYSPVKAMICKDFTNSCAMALVRDPFFVDQH
HYPVHRLEGVIEPERIVSTFNGRRPYNNKKEYHQYNNRKEQHNVKKDYQQQSNNGKSYHK
PTTKNEYFRTNNRKDVHDFRNSRDVHHNSNTNNQFSNNVVNGNFRNNGSGNNFNNRNPRV
NHADNNSRAGNSQEKRYQPNKGTFQKRNFGLKPKQNFIHQPENQ

> SRAE_2000328700

MGVPAFFRWLSRKYPTIVGDVVEEECQIVDGVRIPIDCTKPNLNFQEFDNLYLDMNGIIH
PCSHPEDRPAPETEDDIFVLIFEYIDRLMAIIRPRKLLYMAIDGVAPRAKMNQQRSRRFR
TAKESADKKKEIQAVKDKLTAQGIPVIQRKKEANWFDGNCITPGTPFMERLAKALRYYIA
YKIKTDPSWAKIHIILSDASVPGEGEHKIMDFIRKQRASPGHDPNIAHCLCGADADLIML
GLATHEANFTIIREEFIPRQMSPCELCNLMGHTLQECDGGIIESSIIKTEPVKRKTNFIF
IRLPILREYLEKELIMYEINFKFDLERAIDDWVFLCFLVGNDFLPHLPSLEIRENAICRL
IKLYKQMASECGGYLTDNGEVNMQRMSMILAELGDAEDEIFKRRQDTEQRFKERNKAMKR
KHSSINKASSFIAPSVDGGLITPVPIKNAHLNNFGSQTKVKAYMARVEQEDCIVAERRLL
SILKPQKSDDVPPLIPQGMVSNNTVVVEESDSEPEDNVKFYEPGWKDRYYEEKFGVSFSD
VSLEFRRSVAEAYMEGLCWVLKYYYTGCPSWDWFYPYHYAPFASDFDKIEYYKPNFPKNT
KPFNPLEQLMGVFPAASSQHVPECWRKLMTDIDSEIYDFYPTDFAIDLNGKKHAWMGVAL
LPFVDQERLLACLEKYSDGLTEDEKKRNIRGENHIYVSKYHQAFKEIRGIYETNQDVTWI
SLNPASNNGVSMLISRMVDCVNPDEPYYSPIKTMIYKDFTNSSAMALVRDPFFVDQHYYP
VHRLEGVIEPERVLPLTNFYNSDYKRDYRKNNGSRDDSNFRNNYRNDYNYKKGYRGNNYV
NRGFRNDYSNRPIRRGMR

> SPAL_0000213400

MGVPAFFRWLSRKYPSIVGDVVEEECLTADGARLPVDCTKPNPNFQEFDNLYLDMNGIIH
PCSHPEDRPAPETEDDIFVLIFEYIDRLMAIIRPRKLLYMAIDGVAPRAKMNQQRSRRFR
AAKESADKKKEVQAVKDKLMAKDIPIIQRKKEANWFDGNCITPGTPFMDRLTKALRYYIA
YKIKSDPSWANIHVILSDASVPGEGEHKIMDFIRKQRASSGHDPNTAHCLCGADADLIML
GLATHEANFTIIREEFVPRQMSPCELCNYMGHTLQECDGGIMESSLTRIEQPKRKTNFIF
IRLPILREYLEKELMMYDINFKFDLERAIDDWVFLCFLVGNDFLPHLPSLEIRENAICRL
VRLYKQMASECGGYLTDNGEVNMQRISMILAELGDSEDEIFKRRQENEQKFKERNKAMKR
RHVNSNKASSFVAPNVDGGLITPVPIENAHLNNFGGETKIKAYIARVQQEDCITAERRLL
SILKPEKSLDLLSLNLQGFRIVGQNGKVLDEGSDSEPEDNVKFYEPGWKDRYYEEKFGVS
FSDVSLEFRRNVAEAYMEGMCWVLKYYYSGCPSWDWFYPYHYAPFASDFDRIEYYKPNFP
KNTKPFNPLEQLMGVFPAASSQHVPECWRKLMTDVNSEIYDFYPTDFAIDLNGKKQAWMG
VALLPFVDQDRLLDCLRRHSDGLTDEEKRRNVRGENHVYVSKQHPAYKKIREIYENIQDV
TWISLNPASNNGVILLISRMEDCINPDEPYYSPVKAMVCKDFTNSCAMALVRDPFFMDQH
YYPVHRLAGVIEPEKILPVCNNDRHANKKHYQQNNFRKNYNQTNSRKDYTQTDFRNNHNH
SNGNNGKKDFHDFRNTKNNYYNGNKNRNNYSNNKVVNGNSKDRGNGNNYAFKARYNEHHH
HANNQTQGGRRRPTRNHFQERNAKPKYDQDVTP

> SSTP_0000047400

MGVPAFFRWLSRKYPTIVGDVVEEECPIIDGVRIPIDCTKPNLNFQEFDNLYLDMNGIIH
PCSHPEDRPAPETEDDIFVLIFEYIDRLMAIIRPRKLLYMAIDGVAPRAKMNQQRSRRFR
SAKESADKKKEIQAVKDKLMAQGIPVIQRKKEVNWFDGNCITPGTPFMERLAKALKYYIA
FKIKNDPAWSKIHVILSDASVPGEGEHKIMDFIRKQRASSGHDPNTAHCLCGADADLIML
GLATHEANFTIIREEFIPRQMSPCELCNLMGHTLQECDGGVIESSIVKVDPVKRKTNFIF
IRLPILREYLEKELFMNDINFKFDLERAIDDWVFLCFLVGNDFLPHLPSLEIRENAICRL
VRLYKQMASECGGYLTDNGEVNMQRMSMILAELGDAEDEIFKRRQETEQRFKERNKAMKR
KHADISKASSLIAPNVYGGLITPVPIESAHLNNFGSETRKRAYMARVGQEDCNAGGKKLL
KILKPEKATDSSPLNSQVFQAAGPNGKVIDEESDSEPEDNVKFYEPGWKDRYYEEKFGVS
FSDVSLEFRRNVAKAYMEGLCWVLKYYYSGCPSWDWFYPYHYAPFASDFDKIEYYKPNFP
KNTKPFNPLEQLMGVFPAASSQHVPECWRKLMSDIDSEIYDFYPTDFAIDLNGKRHAWMG
VALLPFVDQNRLLACLEKYSDGLTEEEKKRNVRGENYLYVSKYHQAFKEIRAIYENNQDV
TWMSLNPASNNGVTMLISRMVDCINPDEPYYSPIKTMIYSDFTNSSAMALVRDPFFVDQH
YYPVHRLNGVIEPERILPSTNYCGSEYKREYRRNNVSRDDPNFKNNYRNDYNYKKGYRGN
NYINRGFRNEYNNKPIRRGMR

> SSTP_0000048000

MGVPAFFRWLSRKYPTIVGDVVEEECPIIDGVRIPIDCTKPNLNFQEFDNLYLDMNGIIH
PCAHPEDRPAPETEDDIFVLIFEYIDRLMAIIRPRKLLYMAIDGVAPRAKMNQQRSRRFR
SAKESADKKKEIQAVKDKLMAQGIPVIQRKKEVNWFDGNCITPGTPFMERLAKALKYYIA
FKIKNDPAWSKIHVILSDASVPGEGEHKIMDFIRKQRASSGHDPNTAHCLCGADADLIML
GLATHESNFTIIREEFIPRQMSPCELCNLMGHTLQECDGGVIESSIVKVDPVKRRTNFIF
IRLPILREYLERELFMNDINFKFDLERAIDDWVFLCFLVGNDFLPHLPSLEIRENAICRL
VRLYKQMASECGGYLTDNGEVNMQRISMILAELGGAEDEIFKRRQETEKRFKEKNRSVKR
KHANFNKTFSLITPKVDGDIVNQISIENVHLNNFESETKIKAYMARVEQEDCKTAEIRLL
SILKPEQVADMLPLNPQAFQVVGPNGKVIDEESDSEPEDNVKFYEPGWKDRYYEEKFSVS
FSDVSLEFRRNVARAYMEGLCWVLKYYYSGCPSWDWFYPYHYAPFASDFDKIEYYKPNFP
KNTKPFNPLEQLMGVFPAASSQHVPECWRKLMIDSNSEIYDFYPTDFAVDLNGKKHAWMG
VALLPFVDQDRLLACLEKYSDGLTEEEKRRNVRGENHLYVSKHHQSFKKIREIYENNQDV
TWISLNPASNNGITLLISRMVDCINPDEPYYSPVKAMICKDFTNSCAMALVRDPFFVDQH
CYPVHRLDGVIEPERHLPIYNGQKQKSRKDYRQYNNKKEQHNFRKKYQQPNDGKNYYKHN
NKNEHSCANNRKDLRDFKNKNDKHYQMNTNSQFLKKLFNNSFNNTHPGNNLNNHKSKVSH
INNYPRVDNYQEKKYPSTKVTFRRRNCESKPKQNFFHQPDIQ

> SVE_1730200

MGVPAFFRWLSRKYPLIVSDVIEEESLSADGVRKPIDCTKPNPNFQEFDNLYLDMNGIIH
PCSHPEDRPAPETEDDIFVLIFEYIDRLMAIIRPRKLLYMAIDGVAPRAKMNQQRSRRFR
AAKESADKKKEIQAVKDKLMAQDIPVIQRKREANWFDGNCITPGTPFMDRLTKALRYYIA
YKIKSDPSWANIHVILSDASVPGEGEHKIMDFIRKQRASSGHDPNTAHCLCGADADLIML

GLATHEANFTIIREEFVPRQMSPCELCNFMGHTLQECDGGIMETSLTRIEQPKRKTNFIF
IRLPILREYLEKELAMYDINFKFDLERAIDDWVFLCFLVGNDFLPHLPSLEIRENAICRL
VRLYKQMASECGGYLTDNGEVNMQRISMILAELGDSEDEIFKRRQENEQKFKERNKAMKR
RHINSNKASAFVAPNVHGGLITPVPVENAHLNNFGGETKIKAYMARVQQEDCITAERRLL
SILKPEKSLDLLSLNLQGFRIVGQNGKVLDEGSDSEPEDNVKFYEPGWKDRYYEEKFGVS
FSDVSLEFRRNVAEAYMEGMCWVLKYYYSGCPSWDWFYPYHYAPFASDFDQIEYYKPNFP
KNTKPFNPLEQLMGVFPAASSQHVPECWRKLMTDVNSEIYDFYPTDFAIDLNGKKQAWMG
VALLPFVDQDRLLDCLKKHSDGLTDEEKRRNIRGENHVYVSNQHPAYKKIREIYENIQDV
TWISLNPASNNGVILLISRMEDCINPDEPYYSPVKAMVCKDFTNSCAMALVRDPFFMDQH
YYPVHRLAGVIEPEKILPICSNDRHMNKKHYQQNNFRKNYNQTNSRRDFTQTDFGNNHNH
SNSKKDFHDFRNTKSNYYNGNKNRNNYSNNKMVNGDSKDKGNGNNHAFKVKYNDHHHHAS
NQTQGGRRHPTRNRFQERNAKPKYDRDITP

> SVE_1730800

MGVPAFFRWLSRKYPSIVGDVVEEECLTADGTRLPVDCTKPNPNFQEFDNLYLDMNGIIH
PCSHPEDRPAPETEDDIFVLIFEYIDRLMAIIRPRKLLYMAIDGVAPRAKMNQQRSRRFR
AAKESADKKKEIQAVKDKLMAQDIPVIQRKKETNWFDGNCITPGTPFMDRLTKALRYYIA
YKIKSDPSWANIHVILSDASVPGEGEHKIMDFIRKQRASSGHDPNTAHCLCGADADLIML
GLATHEANFTIIREEFVPRQMSPCELCNFMGHTLQECDGGIMETSLTRIEQPKRKTNFIF
IRLPILREYLEKELAMYDINFKFDLERAIDDWVFLCFLVGNDFLPHLPSLEIRENAICRL
VRLYKQMASECGGYLTDNGEVNMQRISMILAELGDSEDEIFKRRQENEQKFKERNKAMKR
KHPSSNKASSFIAPNIDGGLITPVPIASAHMNNFGGETKIKAYMARVQQEDCITAERRLL
SILKPEKSVNSPSVSSQSFQNVGQNGKVLDEGSDSEPEDNVKFYEPGWKDRYYEEKFGVS
FSDVSLEFRRNVAESYMEGMCWVLKYYYSGCPSWDWFYPYHYAPFASDFDKIEYYKPNFP
KNTKPFNPLEQLMGVFPAASSQHVPECWRRLMVDIESEIYDFYPTDFAIDLNGKKQAWMG
VALLPFVDQDRLLDCLKKHSDGLTDEEKRRNIRGENHVYVSKQHPAYRMIREIYESNQDV
TWISLSPTSNNGVTMLISRMADCVNPDEPYYSPIKAMVCRDFTNSCAMALVRDPFFMDQH
YYPVHRLAGVIEPERFLPLPNSYGPEYGREYRKGIRTRGDYNFKNNYRNDFNYRGGYQDS
NNANRGFRRNYNNRTFRHDTR

C. elegans eri-5

> SPAL_0000160500

MKKFKDFDQGIYNEKISENTDQRVLHTADDLLLNASHSFSTKRFPLRILSSTSNRDDYLY
DFSTNTLEIKISKDKLSKFWQEICEKPLKNSGIVLPMEKLTPAEVHAKLVRHNDATWADT
VSIVNIVSPHLIFVRRLTSTYKNFQFTLPDNLEKIQWINSDTELLEDPKCLNLNDCIGYY
VLAPIEEDVYARARIIDVTEDREYMKIIYIDHGTISWVNRKCLAVMEESLFKFRWQVRSI
ALHNLFPYSNDLESSKKVVWTKKHVEAVKDVLSNCSVYKFTHYNRLKRTYAADVVHVQLF
AAENDLKSQNSTPINDVLIAKYKNLFYKNEKHNQPMFNTPIDISDECDEKLDKNNIPSWK
LNFPCSDKVYKPSPIYNFVREDGIIGYSPDHWTLEKLKDKGYLVDGECLVVFLQAPMDNS
HPLRLGGYLVPGDDIKVIQQKCKEEKIINSGVVRVKRLLSELCREIRINTFDKIRKYSEQ
YKVQNKHASISLDDIIVEWKNNRPFYIVTERKAKNNELMFFRAEVTGFHKEINYFLLRIR
YLDFNGTGICCMHEAYKLSKVVAEDMPFNIGFTFDEILIKNENIINDEESFSELISHVNE
KLPFSEPVLIRLNLSGNRKHYPSKDLYSKWPLIVDNVFQTDFKIDSMWKIDNLKEDDTFD
VNSINFKDVSKKYRKYFKEESSTANCMINFYTKYNYMEFSEISQIIC

C. elegans eri-7

> SRAE_1000168200

MRFLINNFVKYLNFRSSTYIIRTFASSKQSKKKDTSIPSLLKERIESYKKILTTEIDLIE
KGLKSNTNGNTKEKNTVFNDIRIKKISFHPVHGKTLEIRDYKLVGLTTKNLKFGIPFDLE
IDKQSFETIVLEYDSTLGKIILKILDRALTWTSSIEQKLCSLKLSSKSNFKNILTFISKP
NFFTAPGWNTMKIIYKGSAAPTTYSDININLIHGLNETQQKAVKASLNSKRNILCINGPP
GTGKTKVIAEIIKQLKAKKKKILVVTPRIDVIVNIMKHFNKEDIKNSCVFGDETNSIDEK
MKGQVKFDDLDLLSNLISESVNTPYSSFIPNYIDLANYLNSSIRIEIVKESQIIYTTVGR
NLWKYLQSAKFVPDVVILEEASQILECAAWQFLLAGKRSIVVGDFNQLTSNFSSIPESEV
QSKCPSILEYLWNNVSSSCKILLNTQYRTNEKIMKWSSKIFYDNAMIAHDNNKNILLSDI
SNIKKNSGYNSPLLIFDLKDFKNSFERQNNSSYFNMNEVLVCVKYVKFLLRNNIKENDIG

IITPYSAQQKEIKKNFNNIKVSTVDGFQGQEKEVIVFCFVRNNKYRQIGFLNNEKRMNVA
LTRAKRQFVFIGNTDMLNGVKSFIELQKIFSDAGKSIDAKKFLSDSNI

> SPAL_0001494200

MKPFNRLLYIVGFQNTKNSGPSVRYLTTSKLLCEKKTKKKPKIHTPIIFEEKLSLFKKGL
KAELYAINDLILSENTEKNSSVRNQAFHNIRAKKTSYDTLYGNVIELSDSKFIGLNPSAF
PKFKAFNLKQNDKQFLSYLMDYNPKNGTILMKLVSSIDDWKEIKENSIFDLLPSAKNSLD
GVLDFLHAGGFTKMPGWKTLESIYKGSLSPVAYSDRPVKFNGEFNTTQKNAIKAALNSKR
QILCIGGPPGTGKTQVIVEILRQLLEDGQKILVVVPRPTVLTNIYERIDLMKYKACAMVG
DESAHIDIKTKSHKDFENLEYMAELIDEIKEGDGNNECIKDYSDLVVSAKLGINRNIIND
SQIIFTSMGRNVMGLIAKTKFTPDVVLLEEGSQILECVSWRFLLSGKRSIVVGDHNQLAT
TLTSESAVKDCQLDISIMEYLWKNLPKVDKIMLDTQYRMNKKIMEWSSKTFYGNAMIADK
SVENITLSDISSIKNDEKFNSPLLVFDSKNCSNFREIMIQRSFANVKEVMVCAKYVKYLL
NNGLKESGIGIITPYSAQKIKIEEHLKGHNIKVSTVDGFQGQQKEVIIFCYVRDNKSKNV
GFLSEKKRMNVALTRAKRQFVFIGNTDMLSTDDAFEELKNTLLNSGVSIDAGQYLL

> SSTP_0000434500

MISRVNYFLRFSNLKNYNFVIRNLSSKKKKLIKPKDTSIPALFKQKLDSAKKLLKDEIDF
VEKPSVKDIGDSSKERNDEEFTNRKVKVSNAIFDSIHGKVVEVFNEEFIGKNNNKLKLGY
PFVLKIYDFNIDTVVLKFDPVFGKLRLKIIDKNVDWNIIRKNEECKLLPSNRNTLTNVLK
FISDTNFFTLPGWNTMKCIYGGEGVPVAYSDRGLKFTQSLNASQQNAVKASLNSKRNIVC
ISGPPGTGKTQVISEIIRQLQKKKKKILIVGPRKDVLTNILKYFDSSNLNGCCMFDEDKH
SFDSKLKSHRKFDDLHLVSNVINDIKNNNDASTDMSKFFKIAQDLSITMKLNIIRSSKII
YTTTGRNIMGLLATADFIPDVVLFDEASQILEPVSWRYLLNGQRSIVVGDPNQLTTSLNS
ENKTDNPMLQTSIVEYLWKNLPTSCKVLLNCQYRTNEKIIKWSSKTFYNNEMYADDSNKN
IVLSDISGIDKKSRFNHPVVVFDSKDCRKFYESRLNTSYINYNEASVCVKYIKFLLKNGI
KKRDIGVITPYSAQRQELEKHLEDVKVSTVDGFQGQEKEVIVFCFVRNNRYKEIGFLSNE
KRMNVALTRSRRQFVFIGNTDMLSCTESFKELRDCLVSAGPTINADKYLLETES

> SVE_0739600

MRLQKIKYSIPSVRYSTSSKIVAEKKDNKKPQIQTPAIFDKRLDFFKKGLKAELKAIYNL
VSLANIKKNLNKKNQVFCNLKAKKISYDTIYGQLIELCDSKLVALNTKFFPKAKPFILKQ
NNKEFFSHLLHYDPKNGTILLKLNSTIDEWKELRENSTFDLLPSHKSPLENVLEFLHSGR
FTKMPGWKTLELIYKGSRSPVAYSDPPVKFNGEFNTAQQNAIKAALNPKRKMLCIGGPPG
TGKTQVIVEILRYLLEDRKKILVVVPRPDVLTNIYERIDLMKYKSCAMVGDESAHIDMQT
KNHRDFEYLEDMTDIICEIKGEDVDSSSIRDYIGLVDGAKKKINLSIVNNSQIVFTSAGR
NVMGLIELSKFTPDVVLVEEGSQVLECVSWRFLLSGKRSIVVGDHNQLVTTLNSESAAKD
YQLDNSIMEYLWNNFSKVDRIMLDTQYRMNKKIMEWSSKIFYDNAMIAHDSVENITLSDI
SSIKNNNKFNSPLLVFDSKNCSDFKETIIGRSFANIKEVFVCVKYVRYLLQNGLKESDIG
IITSYNKQRMKIEEHLKGKNIKVSTVDGFQGQQKEVIVFCYVRDNKNKNVGFLSEEKRMN
VALTRAKRQFVFIGNTNMLSTDDKFEELRNILLNNGVSLDAVLQNHVNLITIMDDASPFH
PTPRERIPLPQRQNHTSNLSSPPPHLIFIFTKAPTYFFLKHAIIPIPVNVKIKVKKI

C. elegans ekl-1

> SRAE_1000250800

MSKKSLQKFGVRQNTVERFILGNKYRIPYLIIPQKAYFKIVKVISPSIIIVKLLNAITES
INESFDILYHTKLNIEDKFYGNIKPFCDDICEGFLFRYCLAPTGDNKYGRGRIVEEAFQL
TNNEILDAKKFVKVFFIDTGDEGWFNVDSLYEIPIEKYLIPWQVTIVSLNGVTPSGDDIK
YWNENVCLELDNILKQFSFVEIINTNVNINIPNNITSVKMIGFCDAKEIIGENIACKLFF
KLPSYVRYTQFSTSNNIFDTLKEISINNDKKLFKDNIPIIESFDDLRLKIECKCENITYD
EISNNKELKITKLKEKKKQPDVNLLIQKEIGILSYDTFYSDYKKNGENFYESLMFYSPQI
PIWTEELLEKFNYIGTDNKMYLELLPFEETSFGEEIVKNPLEMHAAMLRYQPEDVVIRED
TLYNTLELEYYSERTAFQKKLNYFYSQPGNICPLNQMKVLKDLNNGYSVYGIYFSSSDCD
NFIANRIEVLSVELEKDINDNLQDKEILVLEYQELDIMDKNTKGEIDTNNNNNIFKSIKI
RFLDYGGIVIVTPKFLSKIHSQFCFLPPFSIQINLIPLTQKICSIASEENREFILQYYDY

FNAAIDLKTPMLAIFDSHQGTLRKKNNEEYVPFFEDNCEWEYNHVINISNMQRLFPRGIP
IDMTIDSYINFLENNK

> SPAL_0000859000

MSDEILEKLGFRGNCIENFVLGNKYTIPYLTIPQHAYFKVVKVISPSLIFIKLLNDVTVN
LNESINMEYHKKLNIEEKFYYNTENLNNNVCEGFLYRYCLAPINDKEYGRGRIVKEALQL
TDSKTLISKKFVKIFFIDTAQEGWFSIDSLYELPVEKYLTPWQIAPISLYGIIPSIKKNI
NYWNENICNELNSILKDIMFIEVINKNCGSIITSVKMIGYTDLKRLVGKDIGAQLFCRLP
LDVDYAQFPPSTFFFNTFNEILNNNKKKLFNDDIPFIEMTNNIEKQGNNDSSIVEEKDNI
KVIKREKIKEKKQYYDIKPPVQTTLGVLSFNSFLNCYKNIDNIMEEAKKFCSPQIPIWTK
RLLQKFNYIGPDNKMYVELLPFEETSYGEEIVKNPLELHGALLRYQPEDVVIREDILYNT
LELEYYSERLALQNKLNFFYSQPGHLCPLNQAEVVRDLNKGYCVYGIYFTINDYGILIAN
RVEILSVDMVNDAQSNSNEILDYENMNNEENFDIFNTAKIRFLDYGGIVTVSVKMLSKIH
TQFCFLPPFSIQINLIPLTKKIYSIATEENREFILKYYDYFNAAVDLKTPMLAIFDSHQG
TLRKKRNENYVAFFENNCEWEYNHVINVSSMQRLFPRGIPIDMTIDSYLNFLENTKEEKV
SDVDEEISSNIV

> SSTP_0000307900

MNRSSIFEGISINKEPSKNPTKTVSNKHNNLLLNSDNDYPGKRFPLRIKNFSSNEDDYLY
DLSTNAYEVKISKKDMMEFSERVDRNRLRNSGIILPMEKVTPPEVHAKQVRHNDVRWGDT
ICIANVISPHLIFVKRQTSTYKNFQFTLPDNLEEIEWIDSDEERLDDPRCLNLRDCVGYY
VLAPLIENVYVRARIIDISGDKVYVKVIYIDHGTISWLNRSSLAVMEQDLFKFRWQVRPI
ALYNIYPYSSNKESPKNVLWSHEHIQALKDVLSNHSVLNFTHYNRLKRVYAADIVPVELF
PCENDLTSQYSTSINEILIKKYSHLFYRKEFCNEITSNVLIDISDDCDEKLDIKEMPTWK
LTFPPLEKINKQSNDCIFTREDGIIGFSPDFWTVKKMKDQHYITNSGCVCVFLQAPMDDS
HPLRLGGYLISTDEIEKLREKARNERKIGSGQVQVKRLLSELCHEVRINAYEKIRRYSEQ
YKNFNKNASIKIDDIITEWQNNRPFYIVTERMSRNNELMFFRAEVTGFHEEVNYFLLRIR
YLDFNGTGICCMHEAYKLSKYLADDMPYNIGFSYYDILVKNEAIFGEDKDFIGLVCDVSK
NLSFSEPTIIRLNLINDKEFYPNINKHSNYPLVVDSIFQTDFDVKEMWRIKDLDKDGNFV
MDPVDFDTIIQKYENCFEKKASFLKSAVEFYTRDNFTKFKNPSTEEKDNN

> SVE_1049900

MMNDEVLEKFGFRGNCIENFILGNKYTIPYLTIPQHAYFKVVKVISPSLIFIKFLNDVTG
NLNESINIEYHKKFNIEEKFYYNMENLNNNVCEGFLYRYCLAPINDKEYGRGRIIKEALQ
LTDSKTLNSKKFVKIFFIDTAEESWFSIDSLYELPVEKYLTPWQIAPISLYGIIPSTKKN
INYWNENICKELSSILKDIIFVEVIKKNCGRVITPVQMIVYSDSNRLVGEDIGVQLFCKF
PLEIDYTQYSPSTFVFNSYNKILNNEKKNLFDDNVPFIKMTNNIEEQGNNDSIIFEEKDD
IKIIKCDKIKEKKQYYEIKPPVQTTLGVLSFNSFLNCYKNFNKIMDEANKFCSPQIPNWT
KSLLQKFNYIGPDNKMYVELLPFEETPYGEEIVKNPLELHGALLRYQPEDVVIREDILYN
TLELEYYSERLALQNKLNFYYSQPGNLCPLNQDEVVRDLNKGYCVYGIYFTINDYGILIA
NRVEILAVDMINDAKNNLDENELLDLENMNNEENFNEEDFDIFKTAKIRFLDYGGIVTVS
VKMLSKIHTQFCFLPPFSIQINLIPLTKKIYSIATEENREFILKYYDYFNAAVDLKTPML
AIFDSHQGTLRKKRNENYVAFFENNCEWEYNHVINVSSMQRLFPRGIPIDMTIDSYLNFL
ENTKEDKVSDVDEEISNNIVSKI

C. elegans gfl-1

> SRAE_1000024600

MDYNIQERKKGFKIIKPIIIGNIAKPLDEPITDTSGKSRTHEWTIFVKPYLNEDISKYVK
KVQFKLHESYENNVKVVDSAPFQVCETCWAESEVMIKIFFVDTAEKPVTLYHYVRIREDG
ATFVGSDGTVAAEHYDELIFKEPSALMERCLVEAVKKNELHNNQFRTNFEDIKRIQMDQI
VKARETIRREIDDLKKSIIDGQELLRIKSEELQNILNSKEDEIVTVKQEEVK

> SRAE_2000130500

MENISKKVKEEKKIIKPIVIGNISKPLDKPITDSCGKSRTHEWMIFVKPYLNEDISKYIK
KIQFKLHESYDNNVRVVETPPYQVCETCWAESEVMIKIFFIDSGEKPITLYHYVRIKEDG
ATFVGEDGTIVAEHYDELIFKEPSPLMERSLIEAVKKNEVNNSQFWTNFENSKKIQMEQI
IKARKIIQKEIDDLKKSIIDGQELLRTKSKELQNILNSKEESDTSQGREYR

> SPAL_0000188000

MDISAQERKKGTKIIKPIVIGNTAKLLDEPITDASGKSRTHEWVIYVKPYLNEDISKYIK
KVQFKLHESYENNVKVVESAPFQVRETCWAESEVMVKIFFVDSAEKPVTLYHYVRIREDG
ATFVGTDGLIAAEHYDELVFKEPSPIMERALLDAVKKNDHFNSKFRTNFEETKRIQMDQI
IKAREMIQREIEDLKRSIIDGQELLRLKSEELQSILNSRLEESMIGKQEEVK

> SPAL_0000813300

MEDNRKDIKKDKRIIKPIIIGNISKALDVPITDASGKARTHEWMIFVKPYLNEDISKYVK
KVQFKLHESYENNVIVIENPPYQIHETCWAESEVMIKIFFVDSTEKPITLYHYVRIKEDG
ATFVGDDGTVVAEHYDELIFKEPSKLMERCLMDAIKKNEPNNVQFKTNFEYSRRIQLNQI
AKAREIIQREIEDLKKSIVDGQELLKTKSKELQSILDSRVKQEGNEEESK

> SSTP_0000293400

MDYSIQERKKGTKIIKPIIIGNTAKPLDEPITDSSGKSRTHEWVIFVKPYLNEDISKYVK
KVQFKLHESYENNVKVVESAPFQVCETCWAESEVMIKIFFVDTAEKPVTLYHYVRIREDG
ATFVGADGSVAAEHYDELIFKEPSALMERCLIEAVRKNEPLNNQFRTNFDDIKRLQMDEI
VKAREIIQREIDDLKKSIIDGQEFLRMKSEELQSILNSKEDEVVAGKQEVK

> SSTP_0000808200

MDSSSKKIKESKSIIKPIIIGNISKPLSKPITDTSGKSRTHEWVIFVKPYLNEDISKYVK
KIQFKLHESYDNNVRVVDTPPYQVCETCWAESEVMIKIFFIDSSEKPITLYHYVRIKEDG
ATFVGDDGTIVAEHYDELIFKEPSPLMERSLMEAVKKNELNSNQFWTNFEDSKKTQLEQI
IKARKIIQKEIDDLKKSIIDGQELLKVKSKELQNILDSKAEDNGTP

> SVE_1504600

MDISAQERKKGSRIIKPIVIGNTAKPLDEPILDATGKSRTHEWVIFVKPYLNEDISKYIK
KVQFKLHESYENNVKVVEDAPFQVRETCWAESEVMIKIFFVDSAEKPVTLYHYVRIREDG
ATFVSTDGSIAAEHYDELIFKEPSPYMERCLLDAVKKNNPSNNKFRTNFEETKRIQMDQI
LMARAMIQREIEDLKRSIIDGQELLRLKSEELQSILNSRLEESMIGKQEEVK

C. elegans mes-2

> SRAE_X000042600

MGRQKNFKSPENNIPKKYNYGNNIFAKRPTSLSSSSAQNSIDIENKVYRYLKESDYANVA
DISLCCTVVKVMEQIFDEHEKESLNILRNKNIFKISKETEEKDINKEIHINKTTEWKKMD
LKINIGEKIKCPNMIDDENKVSNCNILVQKFDTIKDNGKTVTPEISKVINVPTVPYKNYI
PNESKCFITTSLNFKTEDKPYYDYVCNAIIKENEESINSKDEENLEDLFPEGVRGTEDDI
FERKFTPEIIMKGVESLTKLFPEKSHDEILQNIVYIYPSIGPLPALKIDLKSYEIEMTKK
TFDVFENFNMCPRCQLLSGCNCNKKKPKGIPHGYIHENKVKLENPRPCSKLCWLLQTNMK
RYTYKENDFPSLTSSKIRELYILFGNKPCLITSDIVEFSKIDIECFKVAKYIEKHLQNTP
CKEYFVLTKDQETPIPYKKFYKNISSYLKSDGTVNNMVIYNPCCHTGPCTKENKCPCAIN
KHICYDSCGCPPNCKTKFTGCHCKAGDCSTTRCPCFILGWECLPMTCDSCSFDGIKKKDC
KSCQNCFIQRNLCKNIDIKQSPIAGNGAFAGESILKGEFIIEYKGEVISNEEAERRGRIC
DAKKSSYLFVLNEDEHIDALNYGNSARFINHSSDNPNCAAKVMIVNGNHRIGLYATKNIS
KGEELLFDYRYTNQHKKGFIEKPNKNGS

> SPAL_0000128500

MGRQKNIKLFEKKSMSKNNNIECEIDKSNHLPLLSSSTQNTIDVESKVHRYLKESEFASV
ADFSFCCAAVKVMEQIFDAHEKESLSILKNKNIFKISQKVDHGNDCEEVHVNKTVEWKKM
DYKLSIGEAIKCFNIIEDENKLGNCSILMQKFDTIKDNGKSIVSEVSKVVNVPTVPYNNF
IPNESKSYITTSENFKTEDKPYYDYVCNAIIKENEASINSKDEEKLEDLFPEGVRGKEDD
IFERKFTPEIIMKGIENLSELFPEKSHDDILQNLVYLYPSIGPLPALKIDLKSYEIEMPK
KTNNVFEIFNMCPRCQLLSGCNCNRPTPKGIPHGYIHESKVKIEDSRPCSKVCCLIQSNM
KRYNFKDNDYTPLVASKIRELYILFGNKPCLITNDIIEFSKAKIECFKVAKYVEKYLQDE
ACKEYFVLTREQETPIPYKRFYKNISTYLKEDGTVNNLVIYNPCCHTGVCSIENNCPCAL
DKHICWDACACPPNCKSKFSGCHCKAGDCSTTRCPCFILGWECLPMTCVSCSFDSVKKKD
GKSCQNSFIQRNLSKNIHIKQSPIAGNGAFAGESILKGEFIIEYKGEVISNEEAERRGRI
CDAKKSSYLFVLNEEEHIDALNYGNSARFINHSSDNPNCAAKVMIVNGNHRIGLYATKNI
SKGDELLFDYRYTNQHKKGFIEKPTNKTAST

> SPAL_0000469700

MSLNGTNSLEINNLISPNKLMKRDSVKENSSSQIFEKSFRNSSNVYDRIKSEKCFRENHS
LFNKCDSIKSMVEDFIKEYDEDQIANKEICITIMKSVRIVDAYEAEVSQSRFDILTPVEI
VNKNDLTRSDSAAVICSLEEINEKIRQGARIPNLNMDHKSSKVEYLTGHDYNVHIVDCDS
EVEVHYDMDDEDNEYDPVNIKNASFEIMKPSNVPPYIQTVINFGTPDDPYLHFSIDAIHE
KSDDGGLFDDENELDIFPDGVRGLKENILDFNITSESIYNIISLTQEKHPNVEIDEIFTN
VHFLFPKLGSLKVLKNVVGDIERQIGKIERKDNNDSKSSFIVNSWCNNCCLFMDCTCEKN
NEGTIPLSYVTKLPYDDSEYKDMCGVDCYKNKSNLNRYPKDISEYSKLEQIRFKQYYTLF
GNRSCHIKDTLITDEGVIAKCYRIKFFLDKFCKDLPTKQYIKMTPAQQTLNTYQTFANVA
RRTILTNESEYGNDDKYSPCKHVGECSEKNNCPCIKSKHACFNMCQCLPSCPSKFTGCNC
KSGNCSTTKCPCVKLGWECVSTYCKNCNCDITVDVPINEMCRNSFLQRGFSKRLDIKEST
IAGFGAFATDLIKKGEFISEYKGEIISQEESERRGRVYDSIKMNYLFKLNQLQQVDAYHY
GNVCRFINHSDTNPNVHAKIIVVSGMQKIALIALRNIDPGEELFFNYNYTKHQTKNFVKN
ITGPQGVKRSHRGPIINSEKIDYLWKKFSSKKR

> SSTP_0000236300

MKMDSLISFNEVITKEFKEENFCDKVFGKRGKNSTNVYDKIKSEKTFKETHLLFNKSDSI
TSRVEDFINEHDEDQIANKDLCITIMKSMRIVDAYEAEVSQSRFDVLTPIEVVNDHEITK
SDSSVVICSLEALNEKINQGAKIPNLNMDYKSSKVEYLTGHDYNVHIIECDSEDELDYVM
NEEDNEYNPVNIKNASFETIKPSNVPPYIQTVINFGTPDDPYLHFSIDAIHEKSDDVGLF
DDENELDIFPDGVRGLKENILDFNITSETIYNIISLTQEKHPNIEIDEIFTNVHFLFPKI
GSLKVLKNVVCDIERQIGKIRNKKNSESKTSFITDSWCNNCCLFMDCTCEKPKDGKIPLS
YVTKLPYDDSEYKDMCGVDCYKNKSNQSKYPKDISEYSKLEQIRFKQYYILFGNRSCHIK
DTLITDEGVIAKCYRIKVFLDKFCKGLPTKQYIKMTPSQQSLNTYQTFANVARKTILINE
SEYGNDDKYSPCKHFGECSERNNCPCIKSKHACFNMCQCLPSCPSKFTGCNCKSGNCSTT
KCPCVKLGWECVSTYCKNCNCDITIDVPINEMCRNSFLQRGFSKRLDIKESTIAGFGAFA
TDLIKKGDFISEYKGEIISQEESERRGRVYDSIKMNYLFKLNQLQQVDAYHYGNACRFIN
HSDTNPNVHAKIIVVSGMQKIALIALRNIDPGEELFFNYNYTKHQTKNFVKNISGPQCVK
RNHKGPAIKAEKIDHLWKKFNSRKK

> SSTP_0001130100

MGRQKNIKSEKKSISQKYHCDSNVFIKTPTTLSSLSVQNNINIEDNVYQYLKESEFANFA
DIPLCCAAIKVMEQIFDEHEKESLNILKNKNIFKISKETEDKDSNKEIHINKTTEWKKMD
FKLNTGEKIKCPNIIDDENKLGNCNVLVQKFDTMKDNGKTISSEVSKVINVPTIPFNNFI
PNKSKCYITTSGNFKTEDKPYYDYVCNAIIKESEESINNKDEEKLEDLFPEGVRGKEDDI
FERKFTPEIIIKGIENLAKIFPEKSHDEILQNLVYIYPSIGPLPALKIDLKSYEFEMTKK
TSNVFENFNMCPRCQLLSGCNCNITTIKGIPHGYIHENKVKLENPRPCSKLCWLLQTNMK
RYTYKEDDFTSLTSSKIQELYILFGNKPCLITSDIIEFSKINIECFKVAKYIEKNLQNTP
CKEYFVLTKDQESPIPYKKFYKNISSYLKKDGTVNNMVIYNPCCHTGPCTKENKCPCAIN
KHICYNACGCPPNCKTKFTGCHCKAGDCSTTRCPCFILGWECLPMTCDYCSFDSIKKKDC
KSCQNCFIQRNLSKNIYIKQSPIAGNGAFAGENILKGEFIIEYKGEVISNEEAERRGRIC
DAKKSSYLFVLNEEEHIDALNYGNSARFINHSSDNPNCAAKVMIVNGNHRIGLYATKNIT
KGDELLFDYRYTNQHKKGFIEKPTKNGSST

> SVE_1976300

MTSFLDKMGRQKNFKLFEKKSMSKNNNIECEIDKSNHLLLPSSSTQNIIDVDSKVHRYLK
ESEFASVADFSFCCAAVKVMEQIFDAHEKESLSILKNKNIFKISQKVDNDNDCEEIHVNK
TVEWKKMDYKLSIGETIKCFNIIEEENKLGNCGILMQKFDTIKDNGKSIVSEVSKVVNVP
TVPYNNFIPNESKCYITTSENFKTEDKPYYDYVCNAIIKENEASINSNDEEKLEDLFPEG
VRGKEDDIFERKFTPEIILKGIENLSKLFPEKSHDDILQNLVYLYPSIGPLPALKIDLKS
YEIEMPKKTNNVFEIFNMCPRCQLLSGCNCNRPTPRGIPHGYIHESKVKIEDSRPCSKVC
CLIQSNMKRYNFKDNDYTPLVASKIRELYILFGNKPCLITNDIIEFSKAKIECFKVAKYV
EKYLQDEACKEYFVLTKEQETPIPYKRFYKNISTYLKEDGTVNNLVIYNPCCHTGVCSIE
NNCPCALDKHICWDACACPPNCKSKFSGCHCKAGDCSTTRCPCFILGWECLPMTCVSCSF
DSVKKKDGKSCQNSFIQRNLSKNIHIKQSPIAGNGAFAGESILKGEFIIEYKGEIISNEE
AERRGRICDAKKSSYLFVLNEEEHIDALNYGNSARFINHSSDNPNCAAKVMIVNGNHRIG
LYATKNISKGDELLFDYRYTNQHKKGFIEKPINKTAST

C. elegans ekl-4

> SPAL_0000213200

MAVAQTDCWSTSQVTPVESLKKTIHLDSIPKKRTKYNREVEQLGIRLEEGIGITSYKIHG
NDYGVNKRITFSKQRCQKWVFKEFTNPARIDGLALKHWIKESQFKKNEPYVFAKLDKHVN
IPTYTDSEYIKYLQEQKWTRQETDHLFDLCRKFDLKWYVIHDRWEIFKTGDGSKKTKSLV
DMKARYYNILNWLNSARDLNVPPIRYDDEHEKKRKKQLTLLMNRTKEQIEEEEELLMELK
KIEARKRERERRAQDLQKLIRSNESNPNSPAINSAALSPLAIIPKKKIQTKNSIATTSRT
IVTTPKILPQIPPISFDFPSIRWPEFKGVGVHARSAEQKLPSSIGTKKTANIDKIFLHLK
LPYVVDSHEDLVNEYNQFRNEITQLHELKSLLLSSEASLQTLSQQFENEGLPMLNIESRF
RASTAVELGIDDYYNVHGAPNDLNEVVKLGYPSSSRKLMGLLDVAAVANSHARKRKSTIP
QGFLANEMKRSRRF

> SSTP_0000048200

MVSTQTEYWSVPQITSVESLKKTMQLDNIPKKRTKYNREVEQLGIRLEEGNGITSYKIHG
NDYGVNKRITFSKQRCQKWVFKEFTNPARTDGLALKHWIKESQFKKNEPYVFAKLDKHVN
IPTYTDIEYTRHLHEQKWTRQETDHLFDLCRRFDLKWYIIHDRWEIFKNGDDKKKTKSLV
DMKARYYNVLNWLNAARDLNVPPIRYDDDHEKKRKKQLTLLMNRTKEQIEEEEELLMELK
KIEARKRERERRAQDLQKLIRSNESNPNSPAINSAALSPLATMPKKKIQTKNSIATTNRT
IATTPKILPQIPPVSFDFPSIRWPEFKGVGVHARSAEQKLPSSIGTKKTANIDKIFLHLK
LPYVVDSHEDLVSQYNQFRDEITQLHELKSLLLSSEASLQTLSQQFENEGLPPLNIESRF
RASTAVELGIDDYYNVFGIPNDPNEVIKLGYPNSSRKLMGLLDVVAITSSHARKRKSTIP
QGFMANEMKRTK*LPLTKDMSSNGEYAVAYASGDTLKNDDCSSSSKVDTTLPLPTYHVNS
SAVRSNKTTPRSRGNITRPYNEESSRPKNVNSCLFCLGSIWDLIIETAGRYSLLSIVLSI
LGILLLVSCIPFTIIILTQSNNPKETEILQTKDISARSMHFYETFLPPAIGVCPEYGFNC
NNDPNEYIGITQRCDGIIHCTDGSDEENCHGCHSGFSCPSKLDPNVVICLRGNKLCDGIK
HCDDGSDEELFCNRRNCTENEFYCESNNSCIKKEYQCDGDPHCPGEEDEVECSSCNNGAV
LCPSTKKCIPSWNICDGTAQCTDKFDEENCSCNKCSGNNRVMCKKSGFCTTKDRVCDGNI
DCPYGEDEEGCPGTCSINERNSTSKSHLVQALTMNDFVKCNDGKNYIRNYACSGLLRQCD
GVCDNGCDTELSFTCKNGACISRSDRCNGVSDCVDGSDEDNCGCDDDTQYKCASDLNSGL
SKCIDKNLLCDGVRDCPQGDDEVNCKECRNPHAIYCPSTSTCYPSIARCDGISHCPDNSD
EMECSCEECSVHPYNMYICSTSKRCFRKESACTPYPICPSPSEDDVNYCLSLLATKTSIFGRNF

> SVE_1730000

MAVAQTDCWSTSQITPVESLKKTIHLDSIPKKRTKYNREVEQLGIRLEEGIGITSYKIHG
NDYGVNKRITFSKQRCQKWVFKEFTNPARIDGLALKHWIKESQFKKNEPYVFAKLDKHVN
IPTYTDSEYIKYLQEQKWTRQETDHLFDLCRKFDLKWYVIHDRWEIFKTGDGNKKTKSLV
DMKARYYNILNWLNSARDLNVPPIRYDDEHEKKRKKQLALLMNRTKEQIEEEEELLMELK
KIEARKRERERRAQDLQKLIRSNESNPNSPAINSAALSPLAIIPKKKIQTKNSIATTSRT
IVTTPKVLPQIPPISFDFPSIRWPEFKGVGVHARSAEQKLPSSIGTKKTANIDKIFLHLK
LPYVVDSHEDLVNEYNQFRNEITQLHELKSLLLSSEASLQTLSQQFENEGLPMLNIESRF
RASTAVELGIDDYYNVHGAPNDLNEVVKLGYPSSSRKLMGLLDVAAVANSHARKRKSTIP
QGFLANEMKRSRRF

C. elegans mes-6

> SRAE_X000134400

```
MAKTRSSNKRQKFSLRILTKSKRQSIKSTFKNSYKNSKICIKEKKQLLEVAKKSELSSET
PEINQILRRYVIKNKKTLNINNSLLKKVKFITKDRFKKLSSSISSVSGKLQYLPQITIEL
EKAKNEAKNIYDGKFFEPGKDELESFVVGYGDKLYIFEIDEKKKGKKVQMRLTISSGVEE
EEYFAICTSFVYIQKNKYPVVVAGGKCKVLQVFLAHNGKHLHNLFGHSGDINEICASPVD
FEIVASVSNDETCRLYNIRHGVNLATFGGPMGHTHNIISLDFSLCGNYIVTCGSDYKVML
WGLKDKNINSQSISTSSKKSNNFEIVFKKLKIKNVFIKNTSLEEKLKIGKEMILDDEKKN
KLTEQIFKRFPLCQNRMLHSTIIDGVRFYDNYIITKDQSKILSMWKFGKHSDDITGNKEI
LKEQRNYSILKHFVLPNCNETWFHKIEIDPSNNILAVASDTGFIYLYDITKQEFYQQPDN
VLTHYFKKSKQRGVRNITFSHDSRYLLSVGDNYTFSVFKLYN
```

> SPAL_0000145200

```
MVGIHPDHPDMKSMTLIENVKSGFLTGITTRLIVQPLDVLKIRFQLQEEPTIGKERGKYR
NINQAIKTIYKEEGITAFWKGHIPAQLLSQIYAVVQFTTYLQFAKCFTEVFKEYNQNKEI
GDFLAGSLSGSLSITASMPFDVIRTRMVAQGKPKVYKNIFDASKKIFLKEGYRGFYKGLS
ASITQTAPFTGLQFLSYNCLANIWNKMISNNNSPMGSVICGGLSGCFSKFLMYPFDVVRH
RMQVLASDRQGFGKATNHIGMINTFKKILYEESILGLYKGLVASILKAGVMAGLSFCSFELFAEYFRKK
```

> SSTP_0001231600

```
MAKTRRSSKRQSVSLKILAKSKMQSIKSTIKESYKKKKICKNDKKKLLEVAKKVQLSNET
PEINQILRRYAIKSKKKLNINNSLLKKIKFITKERFKKLSSPHPSISKKLQYLSQITVEL
EKAKNDAKNIYDAKFFEPGKDELESFVIGYGNKLYIFEIDEKRKGKKIQMRLTISSGVEE
EEYFALCTSFVYIQKNKYPVVVAGGKCKVLQVFLAHNGKHLHNLFGHSGDINEICASPVD
FEIVASVSNDETCRLFNIRHGVNLATFGGPMGHTHNIISLDFSLCGNYIVTCGSDYKVML
WGLKDKTINTQSFCTTSKKNNNNFDLVFKKLKIKNVFIKNTSLEEKLKISKEMFLDDDKK
NKLAEQLFKRFPLCQNRMLHNTIIDGVRFYDNYIITKDQSKILSMWKFGKHSDDITGNKE
ILKEQRNYSILKHFVLPNCNETWFHKIEIDPSNNILAVASDNGFIYLYDITKQEFYQQPD
NVLTHYFKKSKQRGVRNITFSHDSRYLLSVGDNYTFSIFKLYEK
```

> SVE_0902800

```
MAKTRSYKKRKAASLRILAKSKGQTTKSIVKNNIKNAKICKNYKKKLFEAAKNVKLSSES
PEINQILRRYATCTKKALNLNKSLLKKIRYITKDRFKKTLSSTSITSEKLKYIPQLTLEL
EMAKNEVKNIYDAKFFEPGKDELESFVVGYGNKLYIFEIDEKNKRKKIQMRLTISSGVEE
EEYFAICTTFVYIQKCKYPVVVAGGKCKVIQVFLAHNGKHLHNLFGHSGDINEICVSPSN
FEIVASVSNDETCRLFNIRHGVNLATFGGPMGHTHNIISLDFSLCGNYIATCGGDYKVML
WGLKDEVPNSTGTSTISKGNSNFELIFKKLKIKNVYIKNVSLEEKLEISKEMVLDKEKKS
KLSEQLYKRFPLCQNRMLHSTIIDGVRFYDNYIITKDQSKLLSMWKFGKHSDDITGNREV
LKEQRSYSILKHFVLPNSNETWFHKMEIDPSNNVLAVASDNGLIYLYDITKQEFYQQPDN
ILSHYYKKSKQRGVRNVTFSHDSRYLLSVGDNYTISVFKLQN
```

C. elegans rha-1

> SRAE_2000046600

```
MDCKYYSTKKISPIKILNFDVSVNNSKMILNQILSKEGKSAVDFNTEIISNTQPFTFKST
AKVILDSGNIYYGEGIGQSKRISTAECAYVILQKIFSNKEYITDDKKKIIPYPIVDVPLD
EKLYNDIKAFYKWLKINLPDFTIVDNELKESDFINISPPNLFNLEKEFSEFTNNSNYKRN
SRKTAMDKWSPPKADYNCWTNTFICDSLFKGKNLDEVSKILFNIEKRKTEDYNLEAERQK
LPIYKKKKDIVKMIEESQAILIKSSTGSGKSTQVAQFLLKHYIDNGKGAKFNCLISQPRR
LPAISLAKRVAEERYETIGDSVGYCVRFDKLYPRPFGSIVFGTVGTILKKLSSGLKGISH
IIVDEVHERSLETDFLLIILKKMLSIYKELKVILMSATIDTKQFEEYIDNIKVIEIEGNS
FDVMELYLDEFIQHYKLYPSTFISPPGYDENSNLWRFDYFPEKKLISPLAKYITEQIETS
```

NDVPYDIIRIMVEESCNAMFSSNEIGSILIFLPGWNEILLCIEELTNSSSYDIYWLLPLH
SNLPYENQKEVFKPPPKGKIKIIVATNIAESSITVNDVLYVIDSCKQKKQLVKHNTATCF
FEVSWASKDCMDQRKGRAGRLRNGYCYRLISRNLWHLLPQHNEAEIKTAPLDTIILEIKA
LELGDSVDFLKNSMEEIDEKNIKESEEYLQQLSALDKDKNLTYIGKMMQRLPFAPDTAKC
VITATLFNLADSIAIICGYYNSNLSLFKYPYYQNGISDAILFLCGDFISDHVLPLMALKM
DKIKHEDSQDILSIMKKINHDTINYLILVKNQIFNVLNEQFKNTKFYEYGVSNNKESSAQ
MHVILSLLVKSFYPNIAFQTKKRSFIDMDGDKVSLNKTSVLSFDKNNYENRSPFIIYSQK
IISKYTMFKECSVISPLQLLLFGCNNVIYKGGNSIILDGVFEFHINPKFGQMVIYLKVVI
DNLLQSLCAKGNLSEKESAIKYYVRNLVERISTMAYTVNGYWWCPPPPPPPAPKPIPPPP
PPPPPPPTPAPLPPVTVPPTTIPPTTVPPTTVPPTTLEISTTSSETTTISQEITTTTTKI
SQETTTSSETTTISQEITTTPEITTISQETTTSPETTTTSQETTTSPETTTISQETTTSP
ATTTTSQETTTSPETTTISQETTTSPETTTISQETTTSPETTTISQETTTSPETTTISQE
TTTSPETTTISQETTTSPETTTISQETKTYPKTTTIFQETTTSPETTSSEMVQTITTTTK
CPSTNTCSCCHTDCYFSYNGTKMKFVWGLSILEKHTSKDNELIKDNQITGSWIFNGKTWD
YKSLQKESKNSKNMLQLYETLKKIKIYTTDIKYTDKEKSNNTKNNKEKQKGGKNDTKKQK
DDKKKKEEKKDDKNKKEKKNDEQNNKEEPKDDKNKEDKDANESVNSEEYDTTNANCNKKK
DKKKKEVQKNDKKDEKKDDDNKENKNELLNSEEYDTNNDNCNKKDKKKGSKKKSKKKKD

> SPAL_0000809400

MNSGCQYTSSFKILDFDISLDNAKSILNQILSKEGKPAVCFNIDVMNNAQPFIFRATAEV
VLLSGNIYFGQGMGKSKRLATAECAYNILEEISNNNEYQSTDKKNLPKKGNIPQLLVRVT
EDEALYKDVKALYNWLEIELPDFPVVKNELPEPEYKKISPPTFFDFEGVFRETNRQSPDF
RNSRKNYMNIWSPPIPNYDCWKNSMVDDVLFKEKPLDKISQILLSIENRKTPLMKIEESR
KHLPIYDKKNDIIQAVEESQILLIKSSTGSGKSTQIGQFLLKHYIDNMKGAEFNCIITQP
RRLAAINLAKRVAEERYECVGESIGYCVRFEKLYPRPFGSILYATVGTIIKKLSNGLKGI
SHIIVDEVHERSLETDFLLIILKKMLSNCSGIKIILMSATIDTTQFEKYMNGIRVMELHG
KSYEVMELYLDEFIQHYKIYPSLFVPPPGYDVNSNLWDFNYLPNGKLISPLGTYITEQIE
SSDEIPYDIIKMMVEESCKAMISSNEQGSILIFLPGWSEIILCMEELKTSASEDMYWLVP
LHSNLSFDDQKKVFKSPPKDKFKIIVSTNIAESSITVDDVLYVIDSCRQKKQLINHKSAT
CYYEVSYTSKDCMDQRKGRAGRIRKGYCFRLISRSLWHTLPLHTEAEIKTAPLDSTILGI
KALGLGDSVSFLKDSIEQIDERNIIEAEEYLRQLSALDKNKNITYIGRVMERLPFTPETA
KCVLTATLFNVADSIAFICGYYNSNLPLFNNPFKQLEIADAILQLCGDFISDHILPLLAL
KINTAEYKNVNYSLPILKLINKDNMANLYMVKNQIFEVLKNEFQNTDFHEYGVSSNKDSS
AQMHVIMSLLVKSFYPNIAIQSKKRVFIDMEGYKVALNKISVLCLDKNNYEDRSPFIIYS
QKIITKYTMFKECSVISPLQLLLFGYKEVIYKGGNKLIIDDVIIFDIDPKFGQMIIYLKI
IIDSLLQSLCARGFLSEKEQAIKHYIRNLVERVSTMGYTINGRTFPKKDLIGVTKVNFQEIGGMTNWM

> SSTP_0000804700

MNSLMDCKYFPTNNVSSFRVLDFDVSINNSKSILNQILSKEGKPAVEFDVEIISDTQPYT
FKATANVILQSGNTYYGEGLGRSKRISTAICAYVILQKIYNNNEHTTVGKKNLPKKTTIP
SYVIDVPFDEQLYKDIEALYKWLKIDLPNFKIVNKELNQSEVVKISPPNLFSIEKGFTEL
SVNNNFNRNSRKNSMDNWFPPRPDYNCWSDTVISDKLYMGKSLDEISKILLDIERRKKIN
PKIEGERQKIPIYRMKNDIIKAVEESQTLLIKSSTGSGKSTQVAQYLLKHYIDNGRGAEF
NCLVTQPRRLPAISLAKRVAEERYETIGESVGHCIRFDKLDPRPFGSIVFGTVGTTLKKL
SSGLKGISHIIVDEVHERSLETDFLLIILKKMLFKYKELKVILMSATIDTTKFKEYMDGI
KVLELEGNSFEVMELYLDEFIQHYEFYPSSFVLPPGCDDNSNLWRFNYFPDGRLISPLAT
YIAEQVEMSDEIAYDIIRLMVEESCKAMLSSNDIGSILIFLPGWSEIILCMEELTNSPGS
DMYWLLPLHSNLSFENQKKVFEPPPSGRVKIIISTNIAESSITVNDVLYVIDSCKQKKQL
LNHKTATCFFEVSLTSKDCMDQRKGRAGRLRPGYCYRLISRNLWHLLPQHNEAEIKTAPL
DKIILEIKALELGDPVEFLKDSMEEIDQRNIEEAEEYLQQLSALDKGKNLTYIGRIMQRL
PFAPDTAKCVITATLFNVADSIAIICSYYNSNLSLFKYPFDQDELADVILFLCGDFISDH
ILPLMIMKMDSINCKNKQDISAIQKQINYDNINFLCMVKNQIFNVIREEFKDLKIQEYGV
SNNKSSSAQMHVVLSLLVKSFYPNIAIQTKRRSFVDMEGCKVSLNKTSVLAYDKNNYENR
SPFIIYSQKIISKYTMFKECSVISPLQLLLFGCKNVIYKGRNEIILDDTFKFNINPKFGQ
MVIYLKIIIDDLLQSLCAKGNLSEKELAIKHYIRNLVERILTMAYTINGKTFGKKKLIGG
TKVNFKGIGGATDSK

> SVE_1961700

```
MNSGCQYTSSFKILDFDISLDNAKSILNQILSKEGKPAVCFNIDIINNAQPFIFRAKAEV
VLLSGNVYFGLGMGKSKRVATAECAYKILQEISHNNEYQTTDKKNLPKKANIPQLVVRVT
EDEALYNDVKALYNWLEIKLPDFPIVKNELPEPGCKKISPPTFFDFEGVFREPNHYSPDF
RNSRKNYMNIWSPPISNYDCWKNSMVDDVLFKEKSLEKISQILLNIENRKSPLMKIEESR
KHLPIYDKKNDIIQAVEESQILLIKSSTGSGKSTQIGQFLLKHYIDNMKGAEFNCIITQP
RRLAAINLAKRVAEERYECVGESIGYCVRFEKLYPRPFGSILYATVGTIIKKLSNGLKGI
SHIIVDEVHERSLETDFLLIILKKMLSNCSGIKIILMSATIDTTQFEKYMSGIRVMELHG
KSYEVMELYLDEFIQHYKIYPSLFVPPPGYDVNSNLWDFNYLPNGKFISPLGTYITEQIE
SSDEIPYDIIKMMVEESCKAMISSNEQGSILIFLPGWSEIILCMEELKTSSSEDMYWLVP
LHSNLSFDDQRKVFKSPPKDKFKIIVSTNIAESSITVDDVLYVIDSCKQKKQLINHKSAT
CYYEVSYTSKDCMDQRKGRAGRIRKGYCYRLISRSLWHTLPLHTEAEIKTAPLDSTILGI
KALGLGDSVSFLKDSIEQIDERNIIEAEEYLRQLSALDKNKNITYIGKVMERLPFTPETA
KCVLTATLFNVADSIAVICGYYNSNLPLFNNPFKQLEIADAILQLCGDFISDHILPLLAM
KINTAEYKNVNYSLPILKLINKDNMANLYMVKNQIFEVLKNEFQNTDFHEYGVSSNKDSS
AQMHVIMSLLVKSFYPNIAIQSKKRAFIDMEGYKVGLNKISVLSIDKNNYEDRSPFIIYS
QKIITKYTMFKECSVVSPLQLLLFGYKEVIYKGGNKLIIDDIIIFDIDPKFGQMIIYLKV
IIDNLLQSLCARGFLSEKEQAIKYYIRNLVERVSTMGYTINGKTFPKKNLIGVTRVNFQEIGGMTNWM
```

C. elegans ekl-6

> SRAE_2000277100

```
MQDSLKLLDFVTTFQDVKISKNSIIKFDPIHDAFCEGIKKFKEIGCFEKFEKYSIEFREN
ILISNDPRIDFSILLFYLYNNFLQEIKNNNDVTNELCLSIEQEKILSKSLEFFFSVSVFP
CLESGVGIPITKLLKVPVKEWKKYGNLTECKFILKRSIEFLFKLLESNKKIKSIVVDKFL
ALFISANEQLVYYNINDFEENYNELIENTISSIFVFEALFAQYTNKGSPKWYKISCGLKL
SKFLCRKTGLINFLTAIENLTEAKFFENSVCMNEMANLLGACPSTFSIDEYYTNILEQIF
DLLLFSQEWTVKFRLMFGLLVDIIYKKKSEIINKYFVGSILNPWKRLLEKGIQLDQGTDT
GLWSLNIEKSIILLETYLITGKSILRKKLCSNLLKSGYFYFWLNLTSQLEDDSLLKNTFK
NILFGIISDLADNEKCKLFFNLLMKKGQVSCLIDKSNYFILSSFKKSNLLESEKNDKFRY
SGISLKILDSLPIEDDKGSSIQKIFDNVKLLFNNLDIQLKLLTKFLKVINITKFEKTLLV
QEVEDSKEKETRNRFVNIDEVLENNEEKMFSLMYILSNLMEYFINSTDKMKDSNTLINTT
TIVEIIEVLCIIISRYNRKLVDSGYEEDENLKFSIAVLAAIILVAEDNDVIKKSLNELRR
VLRNFIILSEKFEELLSLRDEAIGIIEIISTFLGVDEVDIKANKYQSPEQSTFKEKKRDL
YEECLEDLKDELEAVKGHGLIVIAREIRKKNLQFLKKDRLQLLFDIVPDYVKDHESYVFL
SGISVLAEIAYLQPDPYLFNLIDMFANYKDKDNVEFRGKLGEAIAKVCKQLGSLAPLYFD
RILGIFLKNFKDEDEIIKASSLNALADLICACKGKKYGSVIYELLMGIDFLIKSNDSTPL
VRRSTLHLLRSIIQSTDTQILLGTVIPIDILTKIYRQLKIIYQSDEDDIVKLHAQLILID
INESIKDSINEIESTDLPKIML
```

> SPAL_0001409900

```
MQTSLKLLDFVTTFHDVKVSKNSIIKFDPIDDAFCRGLEKFKETGCIKKFEEYSNAFQEY
ISISQDPRINFSILLLYLYDDFLEEVRNDSNITDDLCLSVEGEKILLKSLEFFISVAIYP
CLEDGVGVPITKLLRVPMKEWKKYNDLNVRKILLHKSIELLFKLLNSNKKIKFIVLEKFL
AIFISGNEQLVHYGIVNFKEDYNEVIENTVNSVSVFKALFAQYTNKDSPKWYRIVCGCKL
SKFLCYKRGLTNFVTAIENLTDIKFFENSVGMNELAKLLGSCPSSVSLEDYYKNILEQLF
DLLLFSQEWTRKFRLMFGSLIDIVFKKEKKIVDKFFVYRVLNPWTILLEKGIQLDHDTNR
GLWSVEIDQSFVLLETYLTTGKGLLRKFLCSEIIEKGFFYLWISLTSQLEDDLPFRKALK
NILFGVIDNLTDDEKCKLFFDILLKRGKISSIVNTSKYFKVDTFKKSNLIESEQSNFHFD
GILLNILDSVPIEDDNSSSIQAIFENMKLLFDDINIQLKLLTKIFKAINLTKFENQSLVQ
EVEDCKEKEKRNRFVNIDEALEASEEETFLLIFVLANVVEHFIKSTEDIKKFDLISTETI
VGIIDVVCIIIRRYNRKLMKNTYEECETLKFGIAVLAAIILVAENNANITKSLEELGKIL
KKFVILSEKFSALSSLRDEAVGIIEIITNFLGIKLEGIIINESPSSERVIYDTKRSDLFE
ECLEDLKDELEAVKGHGLIIIAREIRNKNLQFLKKDRLQLLFSIVPNYVKDNESYVFLSA
ISVLSEIAYIQPDPYLFNLIDMFVNYSDKENIAFRGKLGEAISKVCKLLGYLAPQYFDQI
LNVLLKDFKNEDEIIKASSLNALADLICACKGTKYGSIIHELLTGINFLIKSKDSTPLVR
RSALHLLRSIIQSTDTQLLLGTVIPLDILTKIYCELKVIYKYDEDDVVRLHAQLTIIDIN
EIIKSSIGEIKDNNLSGITL
```

> SSTP_0000768300

MYKSLQILNFVTTFQDIKITKDSIIKFDPIDDAYCEGIKKLKEVGCFDEFEKYSIAFRKD
ILISQDPRVNFGILLLYLYDNFLNEIKNNNDVTDNLCLSIEEDGVGIPISKLLKVPIKEW
KRFDNLTEYKFLAMFVSANEQLIHYKITKFEENYDHVLESTLNSVSVFEAFFAQYTNKYS
PKWYKIACGIKLSKFLCRKTGLINFLTAIENLTDPKFFENSVCMNEVANLLGSCPSNLSK
EKYYRNILKQLLDILLFSRDWIKKLRLMFGLLIDIIYKKRGKIIDEYFVNSILNPWVRLL
DKGIQLNQDSDTGLWSAEIEKSIILLETYLITGKSLLKECLCNKILKSGYFYFWINLASQ
LEDDSLLKNTFKNILFGIISDLTDDEKTKLFFNLLMKKNQVSCLIEKSNYFQLTTFKKCS
LVETEENKKFYFYGISLKILDPIEDDKCSSLQKIFNNVKLLFNNLDIQLKLQTKFLKVIN
LTKVEKKLLVEEIEDTKEKEKRNRFVNIDEVLEDSEDKTFSLMFILSNLMEHFVNDTKSI
EDYNSLSTSTIVEIIDVVCIIINRYNRKLMDSDYEASENLKFGIAICGAIILVAEDNDTI
KNAFNELRKVLENFVIFSEKFKDLLSLREEAIRIIEIIVTFTGIKEDNIKINKYLSSEQN
TCVKKKKDLFEECLEDLKDELEAVRGHGLIVIAREIRKKNLQFLKQDRLQLLFNIVPDYV
KDHESYVFLSGISVLSEIAYLQPDPYLFNLVDMFVNYPDKNNIEFRGKLGEAIAKVCKLL
GCLAPVYFDRIFNVLLKNFKNEDEIIKASSLNALADLIYACKGTKYNSVIYELLMGIDFL
IKSSESTPLVRRSTLHLLRSIIQSSDTQILLGTIIPIDILTNIYRELKYIYQSDEDDIVK
LHAQLILIDINESIKNSINEIGNTKLSNMIL

> SVE_1725500

MQTSLKLLDFVTTFHDVKVSKNSIIKFDPIDDAFCKGLEKFKETGCIKKFKEYSNAFKKD
IAISQDPRINFSILLLYLYDDFLEEVRNDNNVTDDLCLSVEGEKILLKSLEFFISVAIYP
CLEDGVGVPITKLLRVPMKEWKKYNDLIVRKILLHKSMELLFKLLNSNKKIKFIVVEKFL
AIFISGNEQLIHYDNVDFKENYNKILEDTVNSVFVFKALFAQYTNKDSPKWYKIVCGCKL
SKFLCYKRGLANFVTAIENLTDIKFFENSVGMNELAKLLGSCPSSVSLENYYKNILEQLF
DLLLFSLEWTRKFRLIFGSLLDIVYKKNKKIVNEFFVYRVLNPWTVLLEKGILLDHDNNR
GLWSVDIDQSFALLETYLTTGRGLLRKFLCSKIIEKGFFYLWISLTSQLEDDLQLRETLK
NILFGVINNLTDDEKCKLFFDLLLKRGKISSIVDVSKYFKVNTFKKSNLIESEQSNFRFD
GISLNILDSVPIENDKSSCIQAIFDNMKLFFDDINIQLKLLTKIFKAINLTKFENQSLVQ
EVEDCKEREKRNRFVNIDEALETSEEETFSLIFVLANIVEHFIKSTEDMKNFELISTETI
VGIIDVVCIIIRRYNRKLIKNTYEECETLKFGIAVLAAIILVAENNTNISKSLEELGKIM
KKFIILSEKFSVLSSLRDEAVGIIEIITNFLGIKLEGITINESLSSERVVYDNKRSDLFE
ECLDDLKDELEAVKGHGLIIIAREIRNKNLQFLKKDRLQLLFSMIPNYVKDNESYVFLSA
ISVLSEIAYIQPDPYLFNLIDMFVNYSDKDNVAFRGKLGEAISKVCKLLGYLAPQHFDQI
FNALLKDFKNEDEIIKASSLNALADLINACKGTKYGSIIHELLTGINFLIISKDSTPLVR
RSALHLLRSIIQSTDTQLLLGTVIPLDILTKIYRELKVIYKYDEDDVVKLHAQLTIIDIN
EVIKSSIGEIKDNNLSGITL

C. elegans zfp-1

> SRAE_2000197500

MDGNKEQKSDGSPSVASLIPSGCKPKEKEMISGCCVCSEENGWTDNPLIYCDGPNCDVAV
HQGCYGIIDVPEAEWFCSKCTYALRLLYALDKEKIPSWLVNADPSTEQLLNDPKCELCPY
STGALKQTHDKKWSHVICALYIPEVRFGNVHSMEPVILKDVPFDRYSRPCYLCDDYGNKN
SRDVGACMPCNKSGCKKTFHVSCAQKFGLLCEEGGQSKNVKYCGYCRSHIKKAKLDPNIK
VIPAFKWGESPFTVNRDSPASNENGYLDITPSTTPVHKEKKSLHFGNENSSDSSNFDHMH
KLKYSSQQFPTTSSTSGFCTKNDMTVGSKKIVKNHFNTSFSNDSHSHHKVSTGSLKDYES
YNLPKTVKSYDTGKKESQIVVEDRRRQQSDDEYIVERYNKNTLTNISSNSISKANNLGSS
NSVHNYVPSTTPTETSSSASSPKDSIIHLPSNIELTHKKNNSIIPNSVYISPNVTPTQKE
LTVVTSNLEKCISKVGFNGSSLKLPTSALSSPSSASINSDYDTKNQLIIDGTLYPSCDSE
PSPVSYSYQGQTKAALKRRNYDTVFTSGSSDEKKLKIDKNEHLSVRLNSSAISPLINSNG
VKLSRKSKIYKNGSLMNTTMSVTSSSSSSYNGLNNSSKNEKESVEELEDLCKHVVEDTVS
EIYRETIEKRKAAAAAAVMNTGIDLKRQSSATTSPVPQPGNNSDYNGREVKAEFNFPRER
SNSIKRNSISLTNSSSCLPMSTEDKIEASITATISNNMVHESHSSANSIVTSTTSTNQST
STLEDLLEKQFNRGHTFLLSKAPCDVSQLLTLLQELQKENENMSQKETQLQKRLEHLTTV
NNRLKMTLQQPQVSKVTVSTNTISLEKKEESRRTSTPQSNTHNPSTSNSIQPLVRSGTPV
IITNSSSPSTNQQVAIQNPDQLQLSNNGTSSSSVLSPVLSPVPGTPSSTPLSGQAQQQTY
TNSSTPVQQQATNLLSNQNCSSSTSNIVNTAMTPKTSSSITSSLNLPNNTTLSLVTSSST
PSSNFSTTTISTNDELHSQIKNAALNRVIPPVATRPESRTLSNNLQNNNPLSSFNNRNSV
SNVETSISNPLQQLNSNTINSSAQQSLTNLKIAQDIARAAAMATTSLSNNNVTNNLNNTL
SGTGFSKPSSTDNSPRASSTAPFSAAVTPVSNVNLQGLSTNLQSQFLMSRGSATGTPSLP
NTINQLNSINQIDSQMRDLFAAAHLAQLERNNQLQSIPQVQVSSSSQAFSEQQASLLAQL

MLANQLENSAGLQSQLTNILRSQQQFQVNQLMQGYLPQNSVNMNSPNLGQSVFPTGTPPP
SVSLNTSMMSSALAK

> SPAL_0001039400

MDSNKEKKSDGSPSVASLIPSGCKPKEKEMISGCCVCSEENGWTDNPLIYCDGPNCDVAV
HQGCYGIIDVPEAEWFCSKCTYALRLLYTLDKEKIPSWLVNANPSTEQLLSDPKCELCPY
STGALKQTHDKKWTHVICALYIPEVRFGNVHSMEPVILKDVPFDRYSRPCYLCDDYGNKN
SRDVGACMPCNKNGCRKTFHVSCAQKFGLLCEEGGQSKNVKYCGYCRSHIKKAKLDPNIK
VIPAFKWGESPFTVNRDSPASNENGYLDITPSTTPVHKEKKNLHFDSENLSDDINFDHLH
KTKYSSQQFPTTSSSSTKSELSAGSKKLIRNNLSTSSSTNSHSHHKMSSGSLKDYESYNL
PKTIKSYDNEKKESQSFVEDQKRQNCGDEYTIEKYCTSTPTNFSSALISKANNLGSSNSI
HNSIPSTTSTETSSSASSPKDSILHLQPGLELTRKKNNSIITNSVYNSPNVTPTQKELTV
VTNNLDRCIPKVGFNGGSLKLPTSALSSPSSASINSDYDTRNQLIIDGTHYPSCDSEPSP
VPYSYQGQTKAALKRRNYDTVFVSGNSDEKKAKIDKSEHSSLKLNSSAISPLINSNGVKH
TRKSKIYKNGSSMSMTTSYSGINNRGEKESVEELEDLCKHVVEDTVSEIYRETIEKRKAA
AAAAVVNTGIDTKRQSPSTTSPVPQPGNNNDYNNSKEVIKGEFAFPRERSSSVKRNSISL
TTSNSSLPMSTEDKIEASISATISNNMVHENHSITHSIVTSTTSTNQPVTSTLEDLLEKQ
FSRGHSFLLSKAPCDVSQLLTLLQELQKENENMSQKEALLQKRLEHLTTVNNRLKMTLQQ
PQTSKVTVSTNTIPIEKKEENRKTPTPQYNVHIPSTSSNTQLLGGNGTPTTIINSGSPNT
NSQVTIQNSDQLQSSNNGTSSSSVLSPVLSPVPRTPSSTPLSGQTQQQTYNNSTTPIHQL
QQTNNLLINQNSISSTSNITNTAMTPKTSSSITSNLHLPNSSTLSQVTSSSTPSSNSSAT
TISTNDEIHNQIKNAALNRVIPPVATRPESRTLSNNLQNSNPLSSFNNRNSISTVETSIS
NPLQQLNSNTINSSTHQNLANLKIAQDIARAAAMATTSLANSNVTNNLNTTLPGTGFSKP
SSTDNSPRASSTAPFSAAVTPVSNVNLQGLPTNLQSQFLMSRGSASGTPSLPNTISQLNS
LNQIDTQMRDLIAAAHLAQLERNNHLQSVPQVQVSSSSQAFSEQQASLLAQLMLANQLEN
SAGLQSQLTNILRSQQQFQVNQLMQGYLPQNSVNMNSPNLGQSVFPTGTPPPSVSLNTSMMSSALAK

> SSTP_0000000500

MDNNKEQKSDGNPSVASLIPSGCKPKEKEMISGCCVCSEENGWTDNPLIYCDGPNCDVAV
HQGCYGIIDVPEAEWFCSKCTYALRLLYALDKEKIPSWLVNADPSTEELLNDPKCELCPY
STGALKQTHDKKWSHVICALYIPEVRFGNVHSMEPVILKDVPFDRYSRPCYLCDDYGNKN
NRDVGACMPCNKSGCRKTFHVSCAQKFGLLCEEGGQSKNVKYCGYCRSHIKKAKIDPNIK
VIPAFKWGESPFTVNRDSPASNENGYLDITPSTTPVNKEKKSLHYGNEISSDNSNFDHVH
KLKYSSQQFPTTSSSSGFCTKNEITVGSKKIVKNNFNTSFSNNSNSYHRISTGSLKDYES
YNLPKTVKSYDNGKKESHNIINIEDQRRQQSDDEYITSTNILNNSISKANNLGSSNSVHN
YVPSTTPTETSSSASSPKDSIIHLPSNIEISHKKSNSIIPNSVYISPNITPTQKDLQKDL
QKDIHKDFQKDFQKDLQKELSVVTNNLDKCITKVGFNGNSLKLPTSALSSPSSASINSDY
DTKNQLIIDGTLYPDSEPSPIPYSYQGQTKAALKRRNYDTIFTSSNNDEKKLKIDKNETS
SIKLNNNAISSLINSNSNGVKISKKSKIYKNGSLINTTMSVTSSSSSSSYNNGINNKNEK
ESVEELEDLCKHVVEDTVSEIYRETIEKRKAAAAALNDTKRQSSTTTSPIPQPGNNNNDY
NNKEIKAEFTFQRERSNSTKRNSISLITSNNCLPMSTEDKIEASISATISNNMVHENHST
TNSIVTSTSNQTTSTLEDLLEKQFNRGHTFLLSKAPCDVSQLLTLLQELQKENENMSQKE
TQLQKRLEHLTTVNNRLKMTLQQPQAQAHVSKVNVSTNTTTNLTSSTFIDKREECRKTST
PHSNVNTIPNSNNIQSLIRSGTPIIITNNNSPNINPQVTIQNPDQLQLSNNGTSSSSVLS
PVLSPVPGTSSQTTNLLVNQNCSSSNTAMTSKISSSTTITSTSSSNFPQSTTISTNDELH
NQIKNAALNRIIPPVATRPESRTLSNNLQNNNPLSSFNNRNSVTNVETSISNPLQQLNGN
TINSSTQQSLTNLKIAQDIARAAAMATTSLANNNLSNTLNNTLNNTINNTLSGTGFSKPS
STDNSPRASSTAPISNVGNVGNTNNSSNVNLQGLPTNIQSQFLMSRGSATGTPSLPNTIN
QLNSINQIDTQMRDLFAAAHLAQLERNNQLQSIPQVSSSSQAFSEQQASLLAQLMLANQL
ENSAGLQNQLTNILRSQQQFQVNQLMQGYLPQNSVNMNSPNLGQSVFSTGTPPPSVSLNT
IGLTGGIATGKSTVSKYIKRFNIPVIDADVIAREIVKNGTPVYKKLRESFDGSFFDSETG
ELNREKMAEYIFRDAKKRKLLNSITHSAIRKRIIWEIIKTFFKGHQFIILDIPLLFESGM
SMFVQKIVVVSCNNEIQANRLMKRDNITRIEATNKINAQMPLELKKERANFIIDNNGNIE
ETLEMVDVLISRLRKSWIGLFIKLIIGVIFIFILYIIFVVLRMY

> SVE_0549100

MDSNKEKKSDGSPSVASLIPSGCKPKEKEMISGCCVCSEENGWTDNPLIYCDGPNCDVAV
HQGCYGIIDVPEAEWFCSKCTYALRLLYALDKEKIPSWLVNANPSTEQLLSDPKCELCPY
STGALKQTHDKKWTHVICALYIPEVRFGNVHSMEPVILKDVPFDRYSRPCYLCDDYGNRN

SRDVGACMPCNKNGCKKTFHVSCAQKFGLLCEEGGQSKNVKYCGYCRSHIKKAKLDPNIK
VIPAFKWGESPFTVNRDSPASNENGYLDITPSTTPVHKEKKSLHFDSENLSDDINFDHLH
KTKYSSQQFPTTSSTTSNSSTKSELSAGSKKLIKNNLSTSSSTNSHSHHKMSSGSLKDYE
SYNLPKTIKSYDNEKKESQNFVEDRKRQNCSGEYTIEKYCSSTLTNFSTALISKANNLGS
SNSIHNSVPSTTSTETSSSASSPKDSILHLQPGLELTRKKNNSIMTNSVYNSPNVTPTQK
ELTVVTNNLDRCIPKVGFNGGSLKLPTSALSSPSSASINSDYDTRNQLIIDGTHYPSCDS
EPSPVPYSYQGQTKAALKRRNYDTVFISGNSDEKKAKIDKSEHSSLKSNSSAISPLINSN
GVKHTRKSKIYKNGSSMSMTTSYSGINNRGEKESVEELEDLCKHVVEDTVSEIYRETIEK
RKAAAAAAVVNTGIDTKRQSPTTTSPVPQPGNNNDYNNSKEVKGEFAFPRERSNSIKRNS
ISLTTSNSSLPMSTEDKIEASISATISNNMVHENHSITHSIVTSTTSSTNQPVISTLEDL
LEKQFSRGHSFLLSKAPCDVSQLLTLLQELQKENENMSQKEALLQKRLEHLTTVNNRLKM
TLQQPQISKVTVSTNTIPIEKKEESRKTPTPQYNVHIPSTSNNTQSLGGNGTPTTIINGG
SPNINPHVTIQNSDQLQSSNNGTSSSSVLSPVLSPVPRTPSSTPLSGQAQQQTYSNSTTP
MHQLQQTNNLLINQNSISSTSNITNTAMTPKTSSSITSNLHLPNSNTLSQVTSSSTSSSN
SSTTTISANDEIHNKIKNTALNRVIPPVATRPESRTLSNNLQNSNPLSSFNNRNSISTVE
TSISNPLQQLNSNTINSSTQQSLANLKIAQDIARAAAMATTSLANSNVTNNLNTTLPGTG
FSKPSSTDNSPRASSTAPFSAAVTPVSNVSLQGLPTNLQSQFLMSRGSASGTPSLPNTIS
QLNSLNQIDTQMRDLFAAAHLAQLERSSHLQSVPQVQVSSSSQAFSEQQASLLAQLMLAN
QLENSAGLQNQLTNILRSQQQFQVNQLMQGYLPQNSVNMNSPNIGQSVFPTGTPPPSVSLNTSMMSSALAK

C. elegans mut-2

> SSTP_0000186700

MLNIRKSPLIGKGAVNIVSLDQAETVCDIGKKISIINNSQPNTSTNSSPARGFSLSNGSS
SSIGCESSTLLNHHNNDTLQNTMDQNTTSTTTQSITFDVNLINKTFPWIRKEYEPTLQGL
NDELNDLCNYLKPTNNERLVRYKIFNEVRSVLQATFEHCEVLPYGSMASELFLPSSDIDI
VCFMKHYVPDHLNIAAEAFTNSIHLEVIKKIEGATVPIIKLRNNSTQSMIDVSFDTQNVL
PTVDFLLRFKQRNPKLTPLVLILKQLLQDHNLNTNTDGGLSSYAIVLLVIRLLQIFPKND
YCNDNLINDQDTLAHYFLKFIEYYGVSFDYEKNAIRVRDDGAILTKQEMFREMNSMDSLN
HSLLVIEDPIMPHKDVCKNSYRFAAIKNLWKSMFMTFNEIFENGFQNNDNYFGVSKSFFV
KFIHLRRCSPEIRSIIEKANVNFQGEIGPFPILYPNGQIYSFQYNQIYPPPFGLVNQCVP
GMQLPYNSYTIPVGYLPIPMYNSPPNGVSFPHQLPLPNGGTPHLYPTHFVMPQQIYPGTV
IYATNYNNSCYYNNQNNFQTSNPQGQSTVTSEIEEASEDKDSFSNSESEEGKD
