# Supplementary material for: RNAi-mediated knockdown of daf-12 in the model parasitic nematode Strongyloides ratti
Source: PLoS Pathog. 2019 Mar 29;15(3):e1007705. doi: 10.1371/journal.ppat.1007705 (PMC6457571; doi:10.1371/journal.ppat.1007705)
Supplement: S2 Fig — (PDF) [file ppat.1007705.s005.pdf]

Strongyloides ratti – daf-12 – **SRAE_0000032100**

MNLDNQPNGAALLLAAAAMTRSLNAADVTHLFSSNEAKLILEEHARNVHLQEQ
LNIKNQKNQKTKKKNVSKTSYQNNNSIAAVVAHLASNSDTKKDISYDNSTPLN
SNTCSPTVSEPDTMGVEKKDLVSFSPSNNLANDENDILTSNNDTLNVQSSNKW
SKRKINDKVKVDGVFEDNIIGKKNNEMDVSSNRVPHHRIKDRRKVTLEIMRKI
SAEQQMNTPDDPVIHSPNSAASLCQNNTSFNMASLPNSSIAMTNNGSSFNNVF
QPQQGLASPNENNSNQSNCSNSNCSANEGDTRRRQKTCRVCGDHATGYNFNVI
TCESCKAFFRRNALRPKEFKCPYSDDCEINSVSRRFCQKCRLKKCFQVGMKKE
WILNEEQLRRRKNSRLNHMARNNNNNNNNHINPNNMQNGCISGNVNNVNNVQN
LNNSLANGFVVSNGSVGNPGVIPPSMEQLLLNPSSVNSAVLGQQNFVSSNMAR
VIPNQSNLALLSANLGQHILMHNNRRNPGDLNSRNVGIIQQPNSDMMSPETSI
TPNFNSQIVSGNLLSTTSNIIKQESIIKPDISPTVNETNLSGNDGRFNSSPFQ
QTLSGISRPNVSVQPQPQISQISSTSPIGTIVQDNGEPKVTLSLEQYNQLVNA
AKNTTLDTSMIIKQETHTNSPSDLEAVSNVVMVSPTVAGYPTIPSSFEDNFSV
KTERDYTLSEKDLKELDSIRDSFQCMNEPLDNDQQASTLAKKEHNPTDILNVM
DITMRRLVKMAKRLGAFNEISEAGKFSLLKGGMIEMLTIRGVTVFNADKGVWQ
TPVDGHSQISFNMFDKLRPDIKDTQKKGFLHFFNLLHSDVRKNDLAIDIIVLM
VLFDSKREGLVSQQDKETVEKLHRNYESLLHRYLYSIHKDEAEQRFASIPKAL
VALRKVAENAVTLFLGTGNTTEAASLPKEFFATNY

Strongyloides papillosus – daf-12 – **SPAL_0001591300**

MNTPDDPVIHSPNSAASLCQSNTSFNMASLPNSSIAMTNNGSSFNNVFQPQQG
LASPNENSSNQSNSNCSANEGDTRRRQKTCRVCGDHATGYNFNVITCESCKAF
FRRNALRPKEFKCPYSDDCEINSVSRRFCQKCRLKKCFQVGMKKEWILNEEQL
RRRKNSRLNHMARNSNNNNNNHINPSNIQNGCMGGNVNNVNNVQSLNNSLANG
FLVSNGSVGNPGVIPPSMEQLLLNSSSVNGGVLGPQNFVSSNMARVIPSQQNL
AILSANINQHLLMQNNRRNPNDLNPRNIGMIQQSNSGMMSPETSITPNFNSQI
VSNNLLSTTPNIIKQETILKSDISPSINESNMSGNDGRFNSSPFQPTLSGISR
QSVAVQPQPQISQISSTSPIGTIFQDNDGTKVTLSLEQYNQLVNAAKNTSSDT
NMIIKQEAHTNSPSDLEGVSSVVMVSPSVAAYPTITSSFDDTFSLKTERDYTL
SDKDLKELDSIRDSFQCMNEPLDNDQQASTLAKKEHNPTDILNVMDITMRRLV
KMAKRLGAFNEISEAGKFSLLKGGMIEMLTIRGVTVFNADKGVWQTPVDGHSQ
ISFNMFDKLRPDIKDKQKRGFLDFFNLLHSDVRKNDLAIDIIVLMVLFDSKRE
GLVSQQDKETVEKLHRNYESLLHRYLYSIHKEEAEQRFASIPKALVALRKVAE
NAVTLFLGAGNTTEAASLPKEFFATNY

Strongyloides stercoralis – daf-12 – **SSTP_0001172300**

MNLDNQPNGAALLLAAAAMTRSLNAADVTHLFSSTEAKLILEEHARNVQLQEQ
LNIKNQKNQKTKKKNVSKSSYQNNNNSIDAVVAHLASTSNIKKDISFDNSTPL
NSNTCSPAGSEPDAISIVKKDSISFSSLSNNLANDENDVLTSNNDTLSIHKSN
KWLKRKINDTKKVNEKDKVDSIFEDNIIEKKNNDLDGSNNRVPHHRIKDRRKV
TLEIMRKISAEQQMNTPDDPVIHSPNSAASLCQNNTSFNMASLPNSSIAMTNN
GSSFNNVFQPQQGLASPNENNSNQSNCSNSNCSANEGDTRRRQKTCRVCGDHA
TGYNFNVITCESCKAFFRRNALRPKEFKCPYSDDCEINSVSRRFCQKCRLKKC

FQVGMKKEWILNEEQLRRRKNSRLNHMARNNNNNNNNNNNHINPNTIQNGCIS
GNVNNVSNVQNLNNSLANGFLVSNGSVGNPGVMPPSMEQLLLNSSSVNSAVLG
PQNFVSSNMARVIPNQSNLALLSANLGQQILLHNNRRNPNELNSRNVGIIQQP
SSGMMSPETSITPNFNSQIVSGNLLSTTSNIIKQEPIIKSDISPTVNETNLSG
SDGRFNTSPFQQTISGISRPNVAVQPQPQISQISSTSPIGTIVQDNGEPKVTL
SLEQYNQLINAAKNTSLDTNLEAVSNVVMVSPSVPTYPTIPSSFEDTFSLKTE
RDYTLSEKDLKELDSIRDSFQCMNEPLDNDQQASTLAKKEHNPTDILNVMDIT
MRRLVKMAKRLGAFNEISEAGKFSLLKGGMIEMLTIRGVTVFNADKGVWQTPV
DGHSQISFNMFDKLRPDIKDTQKKGFLHFFNLLHSDVRKNDLAIDIIVLMVLF
DSKREGLVSQQDKETVEKLHRNYESLLHRYLYSIHKEEAEQRFASIPKALVAL
RKVAENAVTLFLGAGNTTEAASLPKEFFATNY

Strongyloides venezuelensis – *daf-12* – **SVE_0996600**

MNLDNQPNGAALLLAAAAMTRTLNAADMSHLFSSTEAKLILEEHARNVHLHEQ
LNIKNEKNQKSKKKNVSKSSYQNNNSIAAVVAHLTSSSDIKKDISFDHSTPLN
SNTCSPTVSEPDIMGIIKKDSLNSSPLSNLANDENDILTSNSVTLNVQSSEKW
SKRKMNDSRKSNEKVKVDNDNTFEDNLNEKKNGELDISNNRVPHHRIKDRRKV
TLEIMRKISAEQQMNTPDDPVIHSPNSAASLCQSNTSFNMASLPNSSIAMTNN
GSSFNNVFQPQQGLASPNENSSNQSNSNCSANEGDTRRRQKTCRVCGDHATGY
NFNVITCESCKAFFRRNALRPKEFKCPYSDDCEINSVSRRFCQKCRLKKCFQV
GMKKEWILNEEQLRRRKNSRLNHMARNSNNNNNNHINPSNIQNGCMGGNVNNV
NNVQSLNNSLANGFLVSNGSVGNPGVIPPSMEQLLLNSSSVNGGVLGPQNFVS
SNMARVIPSQQNLAILSANINQHLLMQNNRRNPSDLNPRNIGMIQQSNSGMMS
PETSITPNFNSQIVSNNLLSTTPNIIKQEAILKSDISPSINESNMSGNDGRFN
SSPFQPTLSGISRQSVAVQPQPQISQISSTSPIGTIFQDNDGTKVTLSLEQYN
QLVNAAKNTSSDTNMIIKQEAHTNSPSDLEGVSSVVMVSPSVAAYPTITSSFD
DTFSLKTERDYTLSDKDLKELDSIRDSFQCMNEPLDNDQQASTLAKKEHNPTD
ILNVMDITMRRLVKMAKRLGAFNEISEAGKFSLLKGGMIEMLTIRGVTVFNAD
KGVWQTPVDGHSQISFNMFDKLRPDIKDKQKRGFLDFFNLLHSDVRKNDLAID
IIVLMVLFDSKREGLVSQQDKETVEKLHRNYESLLHRYLYSIHKEEAEQRFAS
IPKALVALRKVAENAVTLFLGAGNTTEAASLPKEFFATNY
